# Rasputin Functions as a Positive Regulator of Orb in *Drosophila* Oogenesis

**DOI:** 10.1371/journal.pone.0072864

**Published:** 2013-09-12

**Authors:** Alexandre Costa, Cecilia Pazman, Kristina S. Sinsimer, Li Chin Wong, Ian McLeod, John Yates, Susan Haynes, Paul Schedl

**Affiliations:** 1 Department of Molecular Biology, Princeton University, Princeton, New Jersey, United States of America; 2 Department of Biochemistry and Molecular Biology, Uniformed Services University of the Health Sciences, Bethesda, Maryland, United States of America; 3 Department of Cell Biology, The Scripps Research Institute, La Jolla, California, United States of America; 4 Institute of Gene Biology, RAS, Moscow, Russia; University of Surrey, United Kingdom

## Abstract

The determination of cell fate and the establishment of polarity axes during *Drosophila* oogenesis depend upon pathways that localize mRNAs within the egg chamber and control their on-site translation. One factor that plays a central role in regulating on-site translation of mRNAs is Orb. Orb is a founding member of the conserved CPEB family of RNA-binding proteins. These proteins bind to target sequences in 3′ UTRs and regulate mRNA translation by modulating poly(A) tail length. In addition to controlling the translation of axis-determining mRNAs like *grk, fs(1)K10*, and *osk*, Orb protein autoregulates its own synthesis by binding to *orb* mRNA and activating its translation. We have previously shown that Rasputin (Rin), the *Drosophila* homologue of Ras-GAP SH3 Binding Protein (G3BP), associates with Orb in a messenger ribonucleoprotein (mRNP) complex. Rin is an evolutionarily conserved RNA-binding protein believed to function as a link between Ras signaling and RNA metabolism. Here we show that Orb and Rin form a complex in the female germline. Characterization of a new *rin* allele shows that *rin* is essential for oogenesis. Co-localization studies suggest that Orb and Rin form a complex in the oocyte at different stages of oogenesis. This is supported by genetic and biochemical analyses showing that *rin* functions as a positive regulator in the *orb* autoregulatory pathway by increasing Orb protein expression. Tandem Mass Spectrometry analysis shows that several canonical stress granule proteins are associated with the Orb-Rin complex suggesting that a conserved mRNP complex regulates localized translation during oogenesis in *Drosophila*.

## Introduction

Translational regulation of maternal mRNAs localized within eggs and embryos directs many key decisions during animal development. One of the mechanisms these cells use to activate the translation of localized mRNAs is cytoplasmic polyadenylation. It is mediated by Cytoplasmic Polyadenylation Element Binding (CPEB) proteins, a family of sequence-specific RNA-binding proteins which bind to the cytoplasmic polyadenylation element (CPE) located in the 3′ untranslated region (3′ UTR) of target mRNAs. CPEBs were initially identified because of their role in RNA localization and translational regulation during gametogenesis in *Drosophila* [1], [2] and *Xenopus* [3] and later shown to be involved in many other biological processes ranging from synaptic plasticity and learning and memory to cell division and cellular senescence [4].

The *Drosophila* genome encodes two CPEB homologues, *orb* (oo18 RNA-binding) and *orb2*. While *orb2* has just recently been described [5]–[7], the function of the *orb* gene has been well characterized in the female germline. *orb* is required at multiple steps during oogenesis, including formation of the 16-cell cyst, oocyte differentiation and the establishment of both dorsoventral (DV) and anteroposterior (AP) axes in the egg and embryo [2], [8], [9]. The establishment of the DV axis in developing eggs is directed by translational activation of localized *gurken* (*grk*) mRNA, which encodes a transforming growth factor α (TGF-α) homologue [10]. Mutations that disrupt the DV axis pathway produce ventralized eggs in which the dorsal respiratory appendages are either missing or fused. *orb* mutations disrupt both localization and translational regulation of two mRNAs in this pathway, *fs(1)K10* and *grk*, and result in ventralized eggs [8]–[11]. *orb* is also required for the proper localization of mRNAs encoding the posterior determinant Oskar (Osk) to the posterior pole of the oocyte and for activating the on-site translation of these mRNAs [8], [12].

In addition to regulating translation of mRNAs needed for egg chamber development and axis formation, *orb* is also required to localize and activate the translation of its own mRNAs in the oocyte [13] through Orb recognition sequences in the *orb* 3′ UTR. This positive autoregulatory loop ensures the proper expression of Orb targets by promoting the accumulation of high levels of Orb protein in subcellular compartments where its activity is required.

We have previously identified four proteins that associate with Orb in an RNase-resistant messenger ribonucleoprotein (mRNP) complex [14]: the *Drosophila* homologue of the Fragile-X Mental Retardation protein (dFMR1/FXR1) [15], [16], Lingerer [17], Caprin (CG18811), and Rasputin (Rin), which is the homologue of the Ras-GAP SH3 domain Binding Protein (G3BP). G3BP was originally identified as a putative effector of Ras signaling through interactions with the SH3 domain of Ras-GTPase activating protein (Ras-GAP) [18], [19]. The G3BPs are evolutionarily conserved RNA binding proteins involved in an array of biological activities ranging from cell-cycle regulation to mRNA metabolism and stress granule assembly [20]. G3BP proteins are overexpressed in human cancers [21], [22] and interact with pathways implicated in cancer, including Ras, NFκB, and the ubiquitin proteasome system [23]–[25]. G3BPs have been shown to function as sequence-specific endoribonucleases [20], [26], [27] and to harbor non-processive DNA and RNA helicase activities *in vitro* [28]. These biochemical activities of G3BPs are thought to be important for repressing the translation of specific mRNAs [29]. Its known targets include β-F1 ATPase, Tau, and c-Myc mRNAs [26], [29], [30]. In addition, G3BP both self-associates into aggregates and interacts with specific protein partners like Caprin-1 and the ubiquitin protease USP10 [25], [31]. These protein-protein interactions are thought to be important in the formation of stress granules. Stress granules (SGs) are storage sites for abortive translation initiation complexes formed in the cytoplasm of cells subjected to environmental stress (reviewed in [32]). mRNAs stored in SGs are directed to either degradation or translation re-initiation depending on the composition of the mRNP complex in which they are packaged. Similar granules are found in mammalian neurons (neuronal granules) and early embryos (polar and germinal granules) where they regulate the localized translation of associated mRNAs. Knockdown of G3BP or its ubiquitin protease partner USP10 blocks the assembly of stress granules, whereas overexpression of G3BP or Caprin promotes it [31], [33].

While mammals express three G3BPs encoded by two genes, *Drosophila* encodes a single G3BP homologue, Rasputin (Rin), which shares 40% identity and 60% similarity with mammalian G3BPs. Genetic studies support a role for Rin in Ras signaling [34]. Males and females homozygous for null mutations in *rin* are viable but sterile, and display defects in photoreceptor recruitment and ommatidial polarity in the eye [34]. Genetic interactions between *rin* and Ras signaling pathway components suggest that Rin functions downstream of Ras, but independently of the MAPK pathway. Moreover, a specific genetic interaction between *rin* and *RhoA* suggests that Rin may provide a link between Ras and Rho signaling [34].

Here we report that Orb and Rin physically associate in an RNase-resistant complex in the female germline. Consistent with the physical association, confocal imaging studies confirm that Orb and Rin partially co-localize in developing egg chambers. To determine the functional significance of this association, we first isolated and characterized a new null allele of *rin* that affects only the *rin* gene. Genetic and biochemical analysis revealed that *rin* functions as a positive regulator in the *orb* autoregulatory pathway and is required for Orb protein expression. Tandem Mass Spectrometry analysis identified eighteen proteins that are associated with both Orb and Rin in *Drosophila* ovaries. Most proteins are components of the translational apparatus or are known translational regulators. Several are also found in cytoplasmic granules involved in RNA localization, stability, and translation in diverse species. The identities of these proteins suggest that they, like Rin, may impact *orb* activity during oogenesis.

## Results

### Orb and Rin associate as part of an RNase-resistant mRNP complex

**Figure 1:**
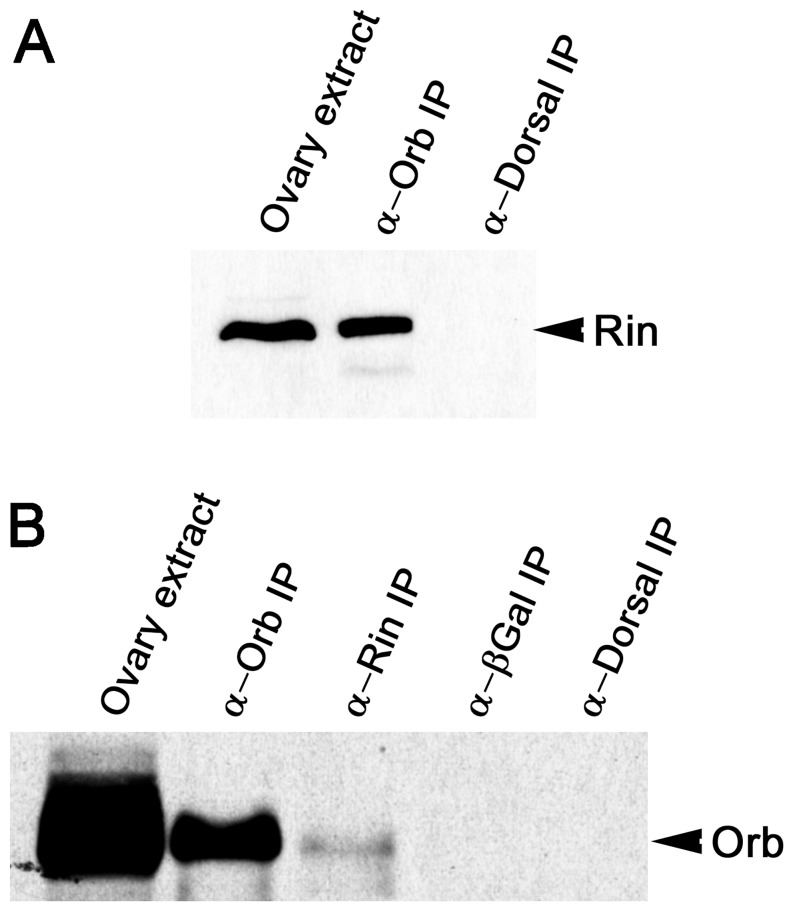
Orb and Rin are components of an RNase-resistant complex. Western blot of proteins immunoprecipitated (IP) with the indicated antibodies and probed on Western blots for Rin (A) and Orb (B). Immunoprecipitations were performed using equal amounts of wild-type ovarian extracts and in the presence of RNase A.

To identify factors involved in Orb protein activity and/or *orb* autoregulation, we searched for proteins that physically associate with Orb *in vivo*. Ovary extracts were immunoprecipitated with anti-Orb or control antibodies and isolated proteins were fractionated by SDS polyacrylamide gel electrophoresis. Bands specific to the Orb immunoprecipitates were then analyzed by mass spectrometry. One of the proteins specifically associated with Orb was Rin [14]. To further confirm this association, ovarian proteins were immunoprecipitated in the presence of RNase-A with Orb antibodies and probed with antibodies against Rin on Western blots (Figure 1A). As expected, Rin co-immunoprecipitates with Orb but not with control antibodies against another fly protein, Dorsal. We further verified the association between Orb and Rin by reverse immunoprecipitation of Orb with anti-Rin antibodies. As shown in Figure 1B and Figure S1A, Orb is detected in the Rin immunoprecipitates but fails to co-immunoprecipitate with antibodies against Dorsal or a heterologous protein (β-Galactosidase).

### Expression of *rin* mRNA

To learn more about *rin* function during oogenesis, we first examined the expression and localization of *rin* mRNA. We found that the pattern of *rin* mRNA accumulation (data not shown) differs in a number of respects from that seen for *orb* [1]. First, *rin* mRNA is detected in both the germline and somatic follicle cells. *orb*, by contrast, is expressed exclusively in the germline. Second, during the previtellogenic stages, *rin* mRNA appears to be distributed more or less uniformly in the nurse cells and oocyte. In contrast, during this same period *orb* mRNA preferentially accumulates at the posterior pole of the oocyte, while there is only little mRNA elsewhere in the egg chamber. Third, after the onset of vitellogenesis, *rin* mRNA expression appears to be upregulated and it accumulates to high levels in the nurse cells but not in the oocyte. At this point in oogenesis, *orb* mRNA is localized along the anterior margin of the oocyte. Although *rin* mRNA is largely absent from the oocyte in stage 9–10 egg chambers, much of it moves into the oocyte during nurse cell transport (“dumping”). This finding would be consistent with our previous results [34], which showed that high levels of presumably maternal *rin* mRNA are present in early embryos.

### Accumulation of Rin protein

**Figure 2:**
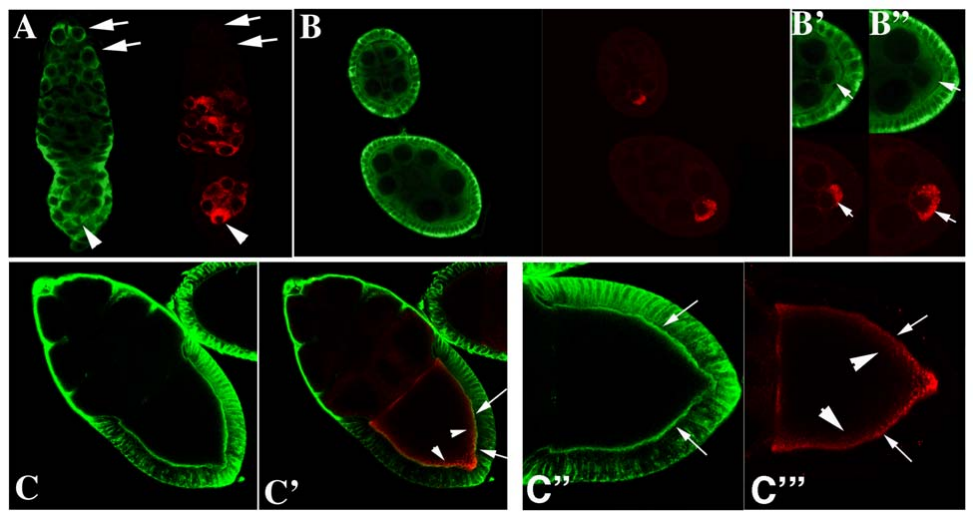
Rin protein expression in ovaries. Confocal analyses of Rin (green) and Orb (red) in the germarium (A), stages 4 and 6 egg chambers (B, B′ and B″), and a stage 10 egg chamber (C, C′, C″ and C‴). Note that Orb is expressed only in the germline and concentrates in the oocyte whereas Rin is expressed both in the germline and surrounding somatic follicle cells. Arrows in A show that Rin is present in stem cells, cystoblasts and 2, 4 and 8 cell cysts while Orb is not. Arrows in B′ and B″ show accumulation of Rin and Orb in the oocyte of stage 4 and 6 cell chambers. Arrows and arrowheads in C′, C″ and C‴ mark overlap between Rin and Orb at the edge of the oocyte cortex in stage 10 egg chambers.

We next examined the pattern of Rin protein accumulation in wild type ovaries using confocal microscopy. Whereas Orb is germline-specific and concentrates in the oocyte (Figure 2, red), Rin is expressed both in the germline and somatic follicle cells (Figure 2, green). In the germline, Rin is present in stem cells and cystoblasts (arrows in Figure 2A, green) at the tip of the germarium as well as in the cysts in region 1 where there is little or no Orb protein (arrows in Figure 2A, red). Detectable levels of Orb are first evident in newly formed 16-cell cysts in region 2 where it accumulates in a subset of cells (Figure 2A, red). In contrast, Rin expression seems diffuse in the germline cysts in region 2 and is more heavily concentrated in the somatic follicle cells (Figure 2A). In region 3, both Orb and Rin are found in all germline cells but appear to be enriched in the oocyte located at posterior pole of the chamber (Figure 2A arrowhead). Stage 1 egg chambers pinch off from the germarium and continue development through 14 morphologically distinct stages which are divided into previtellogenesis (Stages 1–7) and vitellogenesis (Stages 8–14). During previtellogenesis, Orb concentrates in the oocyte with the highest levels at the posterior end (Figure 2B-red; see arrows in enlargements in Figure 2B′ and 2B″-red) while Rin is enriched in the follicle cells, and in the cortical cytoplasm of the nurse cells (Figure 2B) and oocyte (arrows in Figure 2B′ and 2B″-green). During vitellogenesis, there is very little Orb or Rin in the nurse cells, while both proteins are found in the oocyte (Figure 2C and C′ red and green) and Rin is enriched in the follicle cells. Within the oocyte Orb and Rin accumulate preferentially along the oocyte cortex (see arrows in Figure 2C″ and C‴), with Rin being more tightly associated with the cortex than Orb. Although the two proteins appear to overlap along the edge of the oocyte cortex (see arrows in Figure 2C), the Orb protein that is more loosely associated with the cortex (see arrowheads in Figure 2C) does not appear to be in close proximity to Rin. The overlapping yet distinct patterns of Rin and Orb accumulation within the oocyte would be consistent with the co-immunoprecipitation data. Moreover, it suggests the existence of protein complexes that include both Rin and Orb proteins as well as complexes in which only one of the two proteins is present (see below).

### Generation and characterization of *rin^3^*

**Figure 3:**
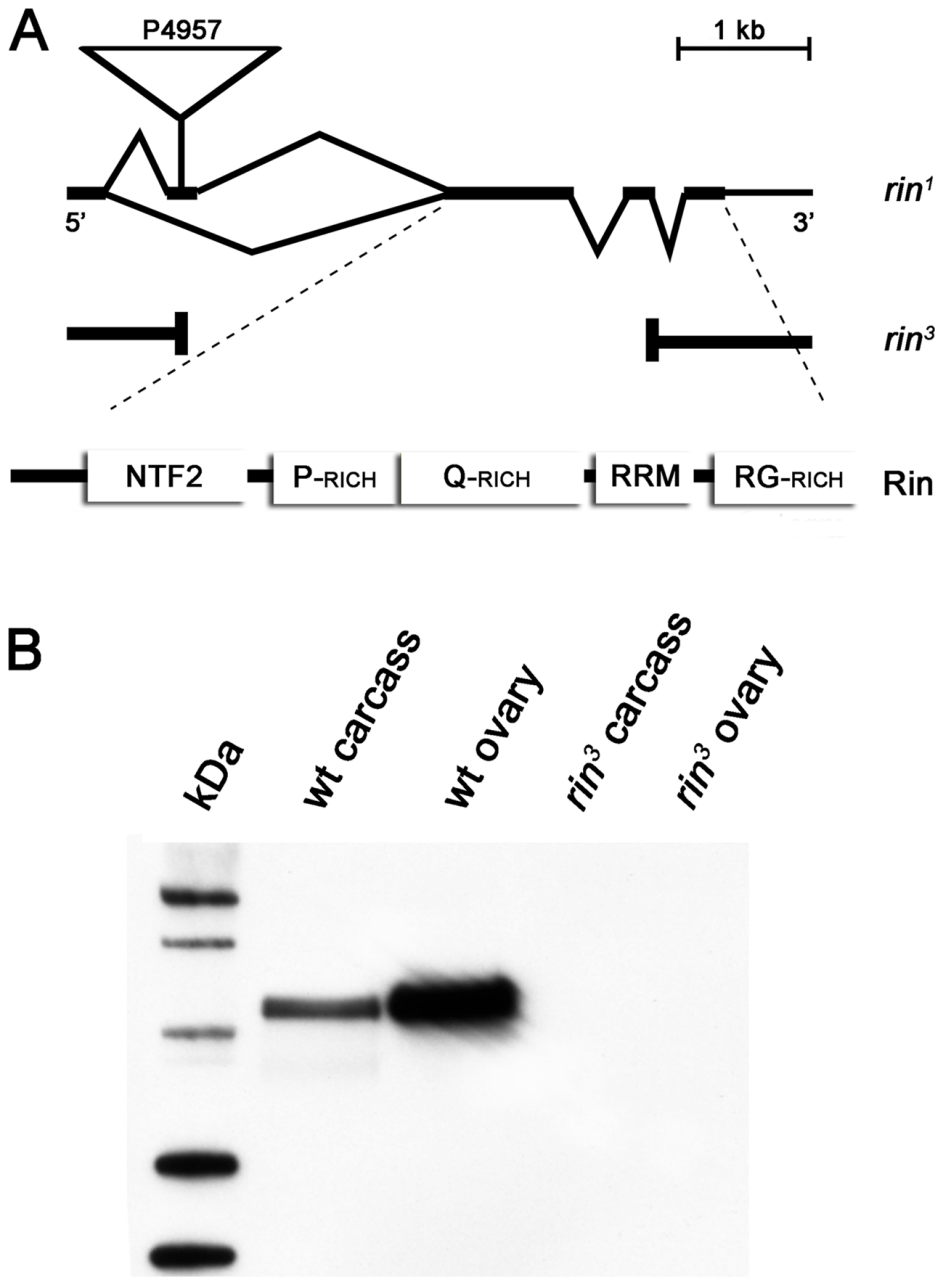
Molecular characterization of the *rin^3^* allele. (A) The 3.3 kb deletion in the *rin^3^* allele was generated by imprecise excision of the P-element P4957 originally isolated from the EMBL lethal collection. The deletion removes DNA encoding the translation start codon, the entire NTF2-like N-terminus, as well as the proline-rich (P-rich) and glutamine-rich (Q-rich) central portions of Rin. The deletion partially affects the RNA Recognition Motif (RRM) at the C-terminus, but leaves the coding region of the arginine/glycine-rich domain (RG-rich) intact. (B) *rin^3^* is a null allele. Western blots of protein extracts prepared from dissected ovaries and ovarectomized wild type (wt) and *rin^3^* females were probed with antibodies against the RRM domain of Rin. Similar results were obtained with antibodies raised against the N-terminal part of Rin (data not shown). Equal amounts of protein were loaded per lane.

To learn more about the function of the Rin protein in *Drosophila* we isolated and characterized a null allele of *rin* that affects only the *rin* gene. Existing *rin* mutations included a weak hypomorphic allele (*rin^1^*) caused by a P-element insertion (P4957) in the 5′UTR of *rin* (Figure 3A) and an imprecise excision allele (*rin^2^*) that deleted multiple genes [34]. To isolate a null allele affecting only *rin*, we generated and screened additional excisions and selected putative excision lines on the basis of female sterility as homozygotes and hemizygotes, as described previously [34]. PCR and Southern analysis of the new *rin^3^* allele revealed that it has a 3.3 kb deletion of most of the coding region (Figure 3A). Homozygous and hemizygous *rin^3^* flies are viable and do not produce any *rin* transcripts (data not shown) or protein as shown by Western analysis of homozygous *rin^3^* flies (Figure 3B and Figure S1B). We also looked at the tissue distribution of Rin by probing Western blots of extracts derived from dissected ovaries and ovarectomized adult females. As shown in Figure 3B, high levels of Rin protein are found in ovaries. This would be consistent with our previous studies, which revealed the presence of appreciable amounts of Rin in early embryos [34] and suggested that *rin* transcripts and protein are maternal contributions to the embryo.

Although *rin* mutants are homozygous and hemizygous viable, *rin^3^* homozygous flies are underrepresented. The reduction in adult viability depended on the strength of the allele (Table S1). Hemizygous *rin^1^*/*Df(3R)urd* flies emerged at a lower than expected frequency compared to their heterozygous siblings. Homozygous and hemizygous null *rin^3^* flies showed a 40% reduction in viability compared to their heterozygous siblings. The reduction in viability was largely rescued by the *Tub-rin* transgene. Additionally, homozygous *rin^3^* flies had a noticeably shorter lifespan than their heterozygous siblings. Analysis of longevity of adult flies revealed a severe reduction in lifespan that correlated with the lack of Rin protein (Figure S2). Homozygous *rin^3^* flies began to die starting 3–4 days after hatching from pupae; after the second and third weeks approximately 35% and 70% of the adult flies were dead, respectively. In contrast, homozygous *rin^1^*, heterozygous *rin^3^*/TM3, and *Tub-rin*; *rin^3^*/*rin^3^* flies had normal life spans.

### *rin* mutants have reduced fertility

To investigate the effects of *rin* mutations on female fertility we measured the hatching rate of larvae from eggs laid by *rin* mutant females. The severity of the allelic combination correlated with the level of reduction in fertility (Table 1). The hatching rate of embryos laid by homozygous and hemizygous females was greatly reduced as compared to wild type females. Furthermore, both homozygous *rin^3^* and hemizygous *rin^3^*/*Df(3R)urd* and *rin^3^*/*Df(3R)l26c* females were completely sterile, but the fertility of *rin^3^* females carrying the *Tub-rin* transgene was close to that of wild type.

**Table 1:** Fertility Assays.

Genotype	% hatched	Eggs hatched/total
*Wild type*	89.8%	1762/1963
*rin^1^*	51.8%	2243/4329
*rin^1^*/*Df(3R)urd*	24.0%	1125/4693
*rin^1^*/*rin^3^*	38.7%	1988/5137
*rin^3^*	0%	0/2223
*rin^3^*/*Df(3R)urd*	0%	0/1504
*rin^Tub-rin^ rin^3^/rin^3^*	86.0%	418/486

*rin^3^* is a homozygous recessive, fully penetrant female sterile mutation. Females of the indicated genotypes were mated with wild-type males. Eggs were collected and allowed to develop for 25 hours at 25°C, and scored for embryo hatching.

### *rin* mutants have a spectrum of oogenesis defects

**Figure 4:**
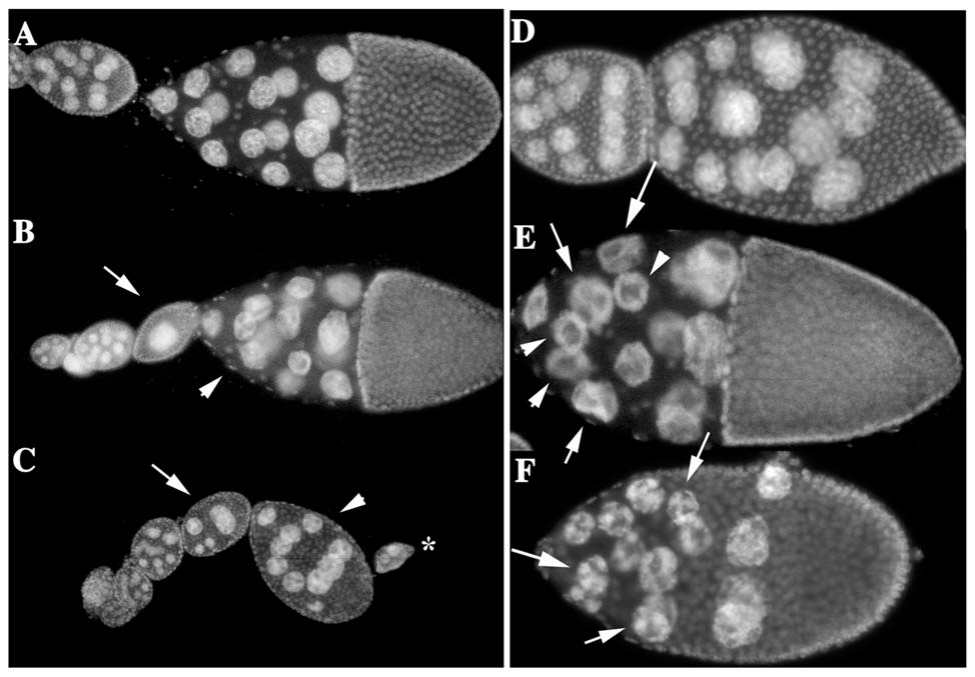
Defects in egg chamber packaging and chromosome morphology. Ovarioles from wild type (A, D) and *rin^3^* (B,C,E,F) females were stained with DAPI. A–C) Wild type chambers always contain 15 nurse cells and 1 oocyte, but *rin^3^* chambers may have fewer nurse cells and sometimes no oocyte. Typically the number of nurse cells/oocyte in adjacent mis-packaged egg chambers adds up to 15 nurse cells and 1 oocyte. In B the arrow indicates a chamber with a single nurse cell while the arrowhead indicates a chamber with fewer than 15 nurse cells. In C there appears to be three related chambers strung together (arrow, arrowhead and asterisk). Each has fewer than the normal complement of nurse cells (and oocyte). About 15% of the *rin* mutant ovarioles had at least one egg chamber with partitioning defects. D–F) In wild type (D) the polytenized nurse cell chromosomes disperse at the onset of vitellogenesis. In *rin^3^* chambers (E, F) the chromosomes fail to disperse. Instead nuclei with discrete chromosomal blobs (arrows) or chromosomes arranged around the periphery (arrowhead) are observed.

We found that *rin* mutants have a range of incompletely penetrant phenotypes that likely together contribute to the female sterility. One of the earliest is a rather unusual defect that affects the proper encapsulation of the germline-derived cells by a single follicular epithelium. In wild type, the egg chamber consists of 15 nurse cells and a single oocyte (Figure 4A). As shown in Figure 4B and C, *rin^3^* ovarioles often have adjacent egg chambers with too few nurse cells. In panel B, the smaller chamber (arrow) has a single nurse cell nucleus, while the larger chamber has 14 nurse cell nuclei. In panel C, the normal complement of 15 nurse cell nuclei and the oocyte appears to be distributed between 3 adjacent incomplete chambers.

A second defect that becomes evident at or before the beginning of vitellogenesis is the abnormal packaging of chromatin in nurse cell nuclei. In wild type, the endoreplicated nurse cell chromosomes have a polytene-like chromosome structure and are organized into 5 domains or blobs until stage 5. At this point, homolog pairing is disrupted and the newly replicated chromosomes disperse throughout the nurse cell nuclei (Figure 4D). In *rin* mutants, the nurse cell chromosomes do not disperse appropriately, and instead of diffuse DNA staining, the chromosomes have a readily discernable structure. In the examples shown in Figure 4E and F, the polytene-like organization of the nurse cell chromosomes persists and discrete chromosomal blobs are still evident within the nurse cell nuclei (arrows). In some cases, the chromosomes appear to line up around the periphery of the nucleus, giving a doughnut-like staining pattern (arrowheads in panel E).

**Figure 5:**
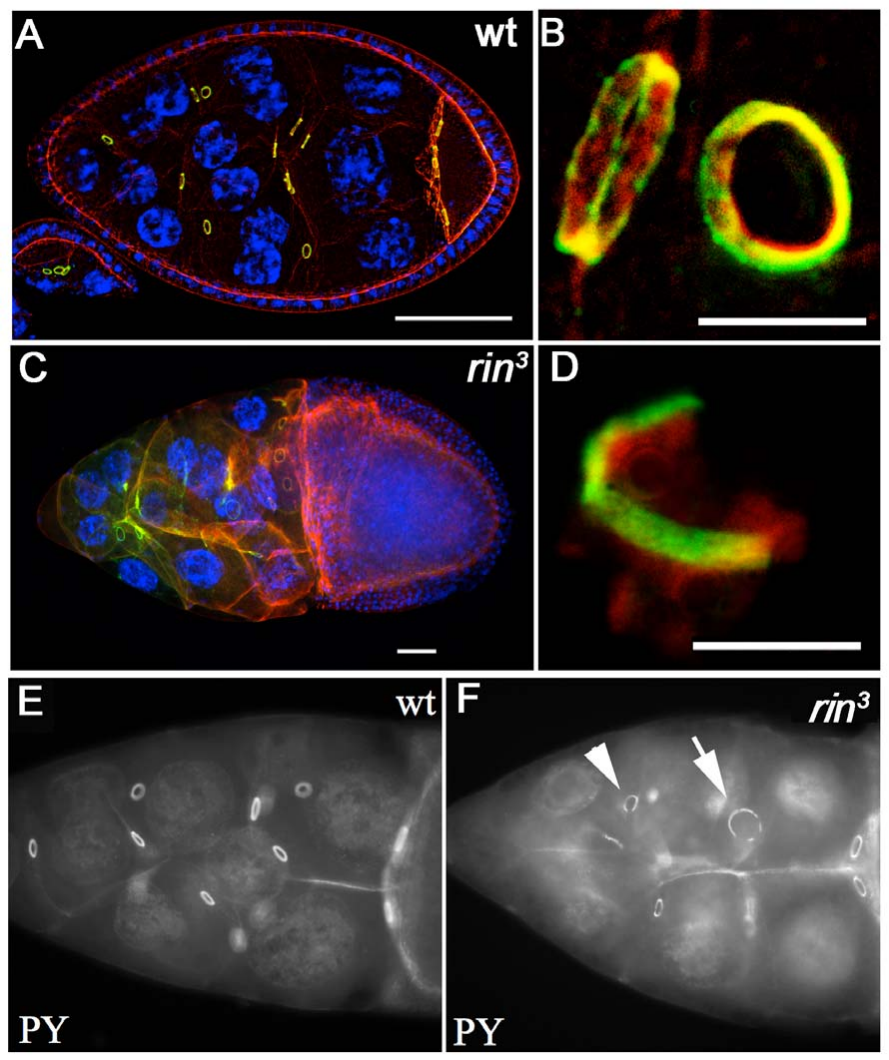
Defects in ring canals. A–D) Wild type (A, B) and *rin^3^* (C, D) ovarioles were probed for actin (red) and phosphotyrosine (green) and stained with DAPI (blue). In wild type (A) each nurse cell has a defined number of ring canals (depending on the number of cell divisions) and the ring canals have a regular circular organization of components. In the *rin^3^* chambers the ring canals often fail to maintain a regular circular structure and may disintegrate (D), possibly resulting in nurse cell fusion (C). Ring canal defects, varying in severity, were observed in most vitellogenic stage *rin* chambers. E, F) Wild type (E) and *rin^3^* chambers probed with phosphotyrosine (PY) antibodies. Note the enlarged (arrow) and fragmented (arrowhead) ring canals in the *rin^3^* chamber.

As might be expected from previous studies which showed genetic interactions with *RhoA* [34], *rin* mutations appear to disrupt the actin cytoskeleton in developing egg chambers. Staining with rhodamine-conjugated phalloidin of *rin^3^* egg chambers reveals defects in the organization of the nurse cell actin cytoskeleton (Figure 5). Actin distribution in nurse cells is abnormal and actin filaments fail to form in stage 10–11 *rin^3^* nurse cells. There are often large actin-free gaps in rin^3^ egg chambers, and subcortical actin is discontinuous. Perhaps the most striking defects are in the ring canals. Ring canals provide channels for transport through the nurse cell complex to the oocyte and are composed of actin plus a number of specialized cytoskeleton proteins including Kelch and Hu li tai shao (Hts). When they are first formed during the mitotic cycles the ring canals are between 0.5 and 1.0 βm in diameter. As oogenesis proceeds, the ring canals grow in size and reach a diameter of about 10 βm by stage 11. As illustrated for actin (red) and phosphotyrosine (green) in Figure 5B, wild type ring canals have a regular circular structure with the different components organized in a sterotypic layering pattern. There are a variety of ring canal defects in *rin^3^* mutant egg chambers that become more prevalent in later stages of oogenesis. Figure 5D shows a ring canal that has fragmented and only a part of the actin-phosphotyrosine ring remains. Actin strings can be seen extending away from this incomplete ring structure. In addition to fragmented or incomplete (arrowhead Figure 5F) ring canals, ring canals that are much larger than normal (arrow Figure 5F) are also observed. As multinucleated nurse cells are often seen in *rin* mutant egg chambers (Figure 5C), it is possible that the nurse cells may fuse following ring canal failure. Perhaps not surprisingly, given the abnormalities in ring canal morphology and the presence of fused nurse cells, one of the terminal phenotypes is dumpless (see Figure S3A, B). As expected from its ability to rescue viability and female fertility, the *Tub-rin* transgene also rescues the defects in partitioning, chromosome structure and ring canals seen in *rin* mutant ovaries (Figure S4A–C).

### *rin* interacts genetically with *orb*

To determine whether the complexes containing Orb and Rin detected in ovaries have functional significance we tested for genetic interactions. For this purpose, we took advantage of the fact that *orb* is weakly haploinsufficient in the *gurken* dorsal-ventral signaling pathway and 5–10% of the eggs laid by females heterozygous for a strong allele like *orb^343^* have ventralized chorions due to defects in the localization and translation of *grk* mRNA [8]. These defects in DV polarity can be further exacerbated by an *hsp83* transgene (*HD19G*) which expresses a hybrid mRNA consisting of the *E. coli* β-galactosidase protein coding sequence fused to the *orb* 3′ UTR [13]. The transgene behaves like a dominant negative because the Orb binding sites in the 3′ UTR of the chimeric mRNA compete with the target sequences in endogenous *orb* mRNAs. This compromises the *orb* positive autoregulatory loop and reduces Orb protein expression. When a single copy of the transgene is introduced into *orb^343^/+* females it increases the frequency of ventralized eggs. *Hd19G orb^343^/+* females lay between 3–30% ventralized eggs depending on the temperature. Loss-of-function mutations in genes that function to downregulate *orb* expression or activity are expected to suppress the DV polarity defects in this assay, while mutations in genes that upregulate *orb* expression or activity are expected to exacerbate the polarity defects.

**Figure 6:**
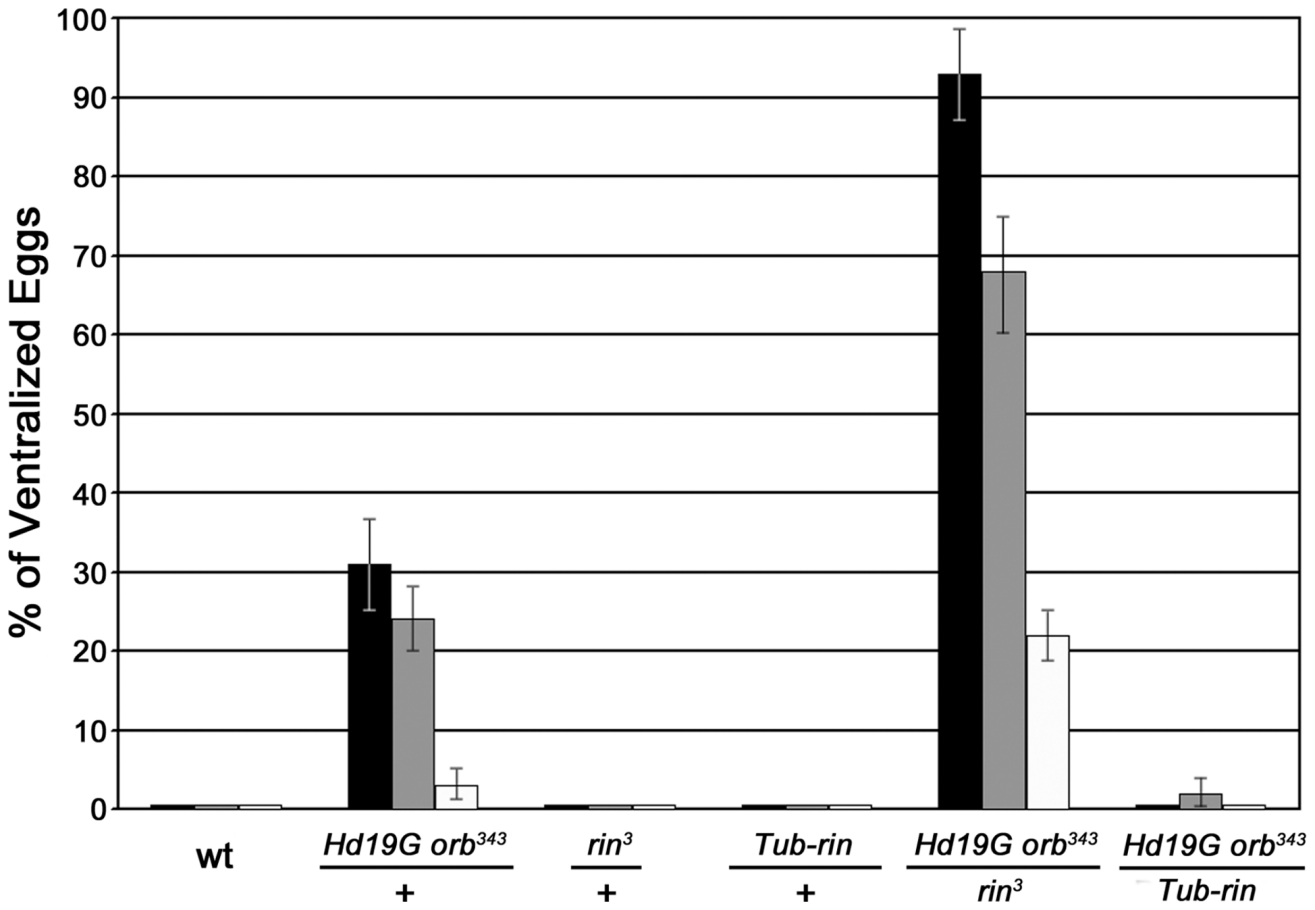
*orb* and *rin* interact genetically. The percentage of dorsal-ventral polarity defects in eggs laid by females with different doses of Orb and Rin is shown. Females of the indicated genotypes were crossed to wild-type males at 18°C (black bars), 25°C (gray bars) and 29°C (white bars). Each of the crosses at the indicated temperature was repeated three or more times and a total of between 1,000 to 2000 eggs were scored. Fused dorsal appendage phenotypes range from fusion at the base to fusion along the entire length of the two appendages. *Hd19G* is a dominant negative transgene carrying sequences of the *orb* 3′UTR bound by endogenous Orb and sufficient to recapitulate the pattern of localization of the endogenous *orb* transcript [13]; *orb^343^*, *orb* null allele [1]; *Tub-rin*, transgene carrying a wild type copy of *rin* under control of the *tubulin* promoter [34].

Because *rin* homologues in mammals repress translation of target mRNAs [29], we anticipated that *rin* mutations would reduce the frequency of ventralized eggs. Instead, precisely the opposite result was observed: the frequency of ventralized eggs is increased 3-fold or more depending on the temperature when *Hd19G orb^343^/+* mothers are also heterozygous for *rin^3^* (Figure 6). For example, at 18°C about 30% of the eggs produced by *Hd19G orb^343^/+* mothers are ventralized, while over 90% are ventralized when *rin* is heterozygous. An even stronger interaction is observed at 29°C. About 3% of the eggs produced by *Hd19G orb^343^/+* mothers are ventralized, while over 20% are ventralized when *Hd19G orb^343^/+* mothers are also heterozygous for *rin*.

**Figure 7:**
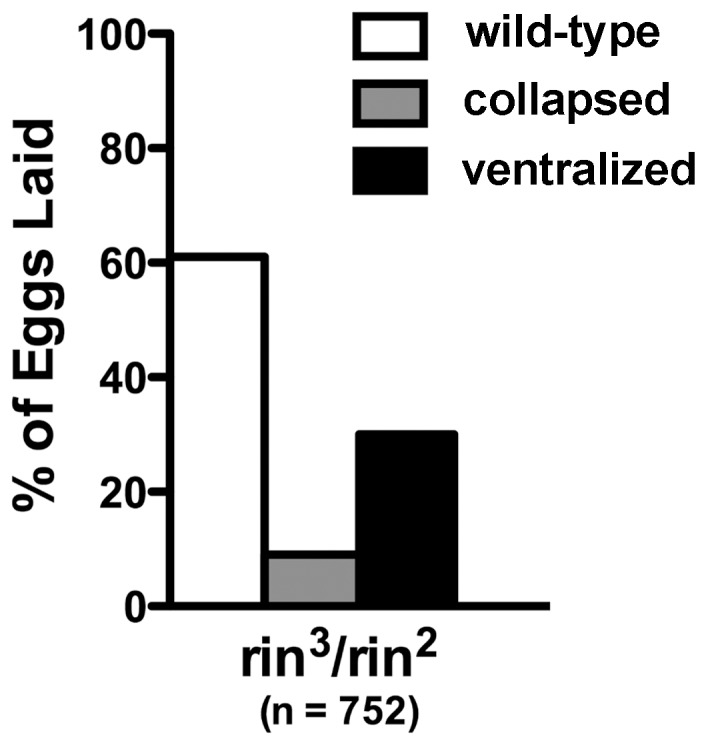
*rin* mothers produce ventralized or collapsed eggs. Egg collections (n = 752) from *rin^3^/rin^2^* mothers were scored for ventralized eggshells and collapsed eggs. No eggs hatched. The frequency of defective eggs of either type in wild type collections is typically less than a percent.

Unlike *orb*, *rin* is not haploinsufficient in the *grk* DV signaling pathway (Figure 6). However, the strong genetic interactions between *orb* and *rin* suggested that *rin* activity is likely to be required in the DV signaling pathway. To test this we examined the eggs produced by *rin^3^/rin^2^* mothers. As shown in Figure 7, about 10% have a collapsed eggshell phenotype. Many of these are likely to be derived from the dumpless chambers that are seen in *rin* mutant ovaries. Approximately 30% of the eggs are ventralized, indicating that like *orb*, *rin* activity is required for normal DV patterning. While the remainder of the eggs has seemingly normal eggshells, cuticle preparations of the embryos produced by *rin^3^/rin^2^* mothers indicate severe defects in embryonic development. For example, many of the embryos are undeveloped or have only scraps of cuticle. Similar embryonic phenotypes have been observed for the *orb* hypomorphic allele, *orb^mel^*.

### *rin* functions as a positive regulator of *orb*

**Figure 8:**
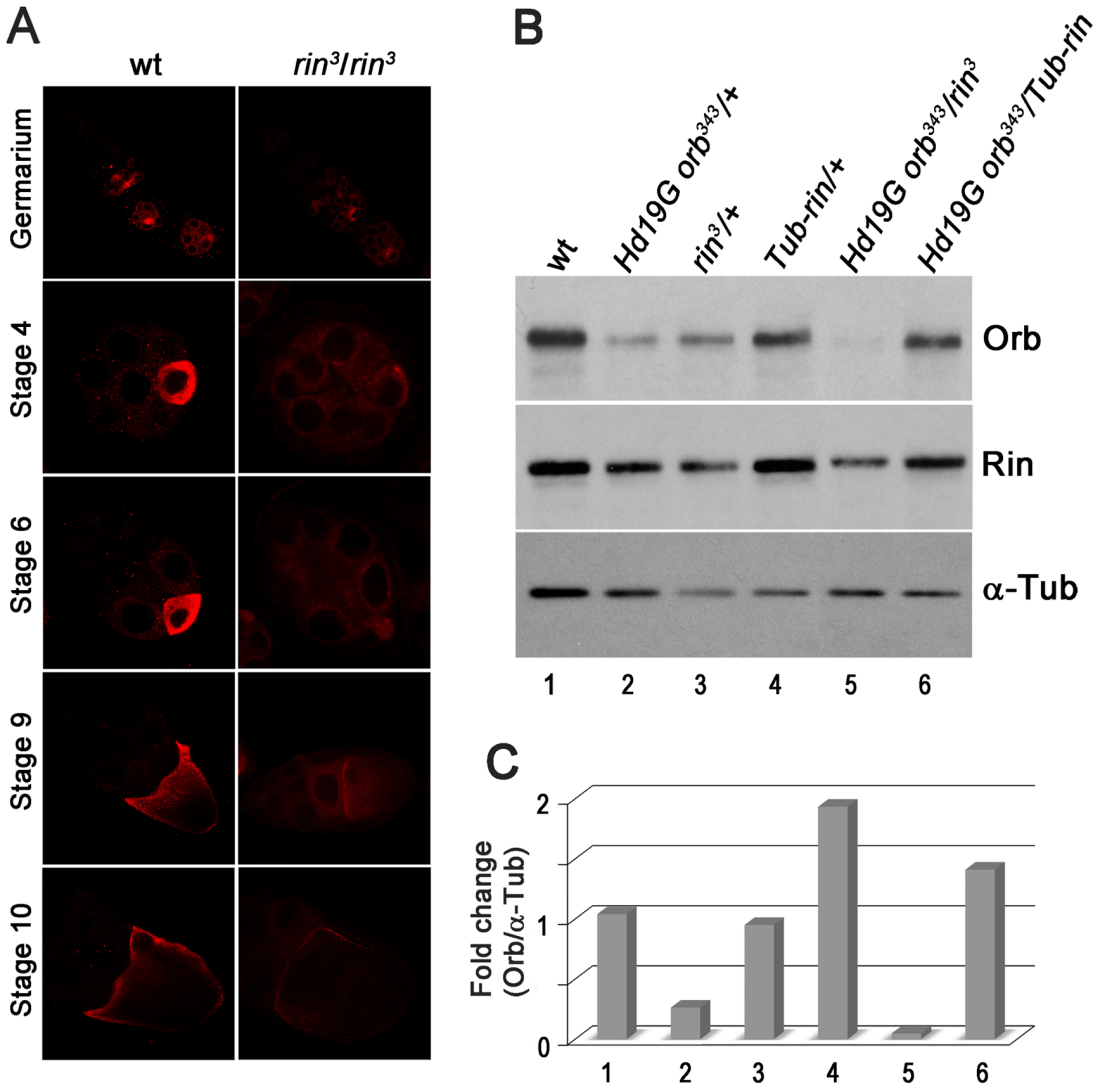
Rin positively regulates Orb expression. (A) Orb expression is downregulated in *rin^3^* ovaries. Confocal analysis of wild type (wt) and homozygous *rin^3^* egg chambers at different stages of oogenesis. Samples were processed in parallel and microscopy was carried out under identical settings. (B) Western blot analysis of ovarian protein extracts derived from the indicated genotypes and probed with antibodies against Orb, Rin, and α-tubulin (control). (C) Quantitation of Orb protein levels shown in (B) using the Quantity One software (Bio-Rad).

One plausible mechanistic connection between the ventralized eggs produced by *rin* mutant mothers and the *orb-rin* genetic interactions is that *rin* functions in *orb* autoregulation, promoting Orb activity and/or expression. Several different approaches were used to explore this possibility. We first investigated Orb protein expression in the absence of *rin* using confocal microscopy. As shown in Figure 8A, Orb protein levels were appreciably reduced in *rin^3^* ovaries throughout all stages of oogenesis. This finding together with the strong *orb-rin* genetic interactions suggests that *rin* might, at least under special circumstances, be a limiting factor in the *orb* autoregulatory loop. One such special circumstance would be when the autoregulatory loop is compromised, as it is in *Hd19G orb^343^/+* ovaries. If *rin* is limiting in this genetic background, then it should be possible to suppress the *Hd19G orb^343^/+* DV defects by increasing *rin* activity using the *Tub-rin* rescue transgene. As shown in Figure 6, the frequency of ventralized eggs is reduced to near background levels at all temperatures when *Hd19G orb^343^/+* mothers have a single copy of the *Tub-rin* transgene.

To further test the relationship between *rin* and *orb*, we examined the effects of different levels of *rin* on Orb protein accumulation in wild type and sensitized *Hd19G orb^343^/+* egg chambers. We first compared the effects of reducing *rin* activity in an otherwise wild type background or in *Hd19G orb^343^/+*. As shown in Figure 8B and C, Orb protein levels are not changed very much if at all by reducing *rin* activity (*rin^3^*/+) in a wild type *orb* background (Figure 8B and C, lane 3). On the other hand, there is a noticeable drop in Orb protein when the *rin* mutation is introduced into *Hd19G orb^343^/+* females (compare Figure 8B and C, lane 5 with lane 2). Consistent with a role for *rin* in upregulating *orb* activity/expression, increasing *rin* activity augments Orb protein levels. Whereas Orb protein levels in the ovaries of *Hd19G orb^343^/+* females are only about 1/4^th^ that of wild type, Orb protein levels in *Hd19G orb^343^/+* females that also carry the *Tub-rin* transgene are even greater than wild type (Figure 8B and C, lane 4). Moreover Orb protein levels can be increased in wild type females by introducing the *Tub*-rin transgene. These results support the conclusion that *rin* functions as a positive regulator in the *orb* autoregulatory pathway and argue that *rin* impacts DV axis specification at least in part by regulating Orb expression/activity. Note that given the range of other phenotypes evident in *rin* mutants, it is quite possible that *rin* may have functions in DV axis specification beyond helping to ensure that there is sufficient Orb protein.

### Identification of additional components of the Rin-Orb mRNP complex

To identify other potential components of the Orb-Rin mRNP complex, we performed Tandem Mass Spectrometry on ovarian proteins co-immunoprecipitated with antibodies against either Orb, Rin or a negative control, Dorsal, in the presence of RNase-A. We identified 22 proteins that associate with Rin only, 132 proteins that immunoprecipitate exclusively with Orb, and 18 proteins that associate with both Orb and Rin (see Table 2 and Table S2). Two of the proteins that are common to both Rin and Orb immunoprecipitates, dFMR1 and Lingerer, were identified previously in Orb immunoprecipitates [14] (Table 2). dFMR1 is a KH-domain RNA binding protein which associates with poly(A) containing mRNAs and functions to negatively regulate translation [35]. It also appears to be a component of the RNAi machinery. In fly ovaries, we found that it negatively regulates *orb* activity [14]. Lig is expressed at high levels in the nervous system and gonads and was initially identified because of defects in mating behavior evident in hypomorphic *lig* mutant males [17]. More recent studies indicate that *lig* functions in the ovary as a negative regulator of *gurken* mRNA translation [36]. The remaining 16 proteins (Table 2) include many known translational factors, namely five 60S and three 40S ribosomal proteins, one elongation factor, and two RNA-binding proteins (Ataxin-2 (Atx-2) and Poly(A) Binding Protein (PABP)). In addition, four known proteins (Poly (ADP-ribose) polymerase (PARP), Heat Shock 70 kDa Protein Cognate 5 (HSC70-5), Rudimentary, and Twenty-four (Twf)) and one novel protein (CG5726) co-immunoprecipitated with both Orb and Rin. Several other potentially relevant proteins are present in Orb, Rin but also Dorsal immunoprecipitates. These include Caprin which is known to be associated with G3BP in other species (and was found previously in Orb but not control immunoprecipitates [14]), multiple ribosomal proteins, and translational regulators like Cup and Lost that have been implicated in DV or AP polarity (Table S3).

**Table 2:** Proteins Present in Orb and Rin but not Dorsal Immunoprecipitates.

	UniProtKB	Orb	Rin
Protein	Accession	Seq ct	Spec ct	% Cov	Seq ct	Spec ct	% Cov
Lingerer (CNS; behavior; Grk signaling) (CG8715)	Q86S05	74	329	41.3%	25	55	23.7%
Drosophila Fragile X Mental Retardation (dFMR1) (CNS: translation factor; Orb regultor) (CG6203)	Q9NFU0	31	65	41.2%	5	9	11.1%
CG5726 (RNAi)	Q7JRH5	30	62	56.1%	17	38	36.8%
Poly (ADP-ribose) polymerase (PARP) (CG40411)	P35875	16	20	22.1%	3	5	5.8%
Elongation factor 1α48D (CG8280)	P08736	12	17	36.1%	2	2	11.4%
Poly(A)-binding protein (PABP) (CG5119)	P21187	8	8	18.8%	10	13	24.1%
twenty-four (circadian translation factor) (CG485)	Q9W4M7	3	3	1.7%	33	72	23.0%
Ataxin-2 (microRNAs,) (CG5166)	Q8SWR8	2	3	3.8%	9	31	20.4%
Rudimentary (pyrimidine biosynthesis (CG18572)	P05990	3	3	3.6%	2	2	2.9%
Heat shock 70 kDa protein cognate 5 (CG8542)	P29845	2	2	4.1%	5	8	14.8%
40S ribosomal protein S2 (String of pearls)	P31009	7	17	30.0%	4	10	19.1%
40S ribosomal protein S14 (CG1524)	P14130	6	7	37.1%	3	7	28.5%
40S ribosomal protein S24 (CG3751)	Q9W229	2	3	20.6%	2	3	20.6%
CG13096 (Ribosome component)	Q9VLK2	5	9	6.9%	2	2	6.0%
60S ribosomal protein L10 (CG17521)	O61231	3	6	19.3%	2	5	13.8%
60S ribosomal protein L11 (CG7726)	P46222	3	3	12.5%	2	2	16.8%
60S ribosomal protein L13A (CG6459)	Q9VNE9	4	8	17.1%	2	3	12.7%

Tandem Mass Spectrometry identification of proteins that co-immunoprecipitate separately with both Orb and Rin. Sequence count (Seq ct) is the number of unique peptides identified for each protein; Spectral Count (Spec ct) represents the total number of times all of the unique peptides were identified for the protein and provides an approximate measure of the protein's abundance in the sample. % Coverage (% Cov) is the percentage of the protein covered by the unique peptides.

With the exception of Rudimentary all of the proteins found associated with both Rin and Orb function in translation, translational regulation and/or RNA metabolism. Moreover, several of the factors besides Rin (Atx-2, dFMR1, PABP, and Caprin) have been previously implicated in translational repression and stress granule formation [32]. Our current and previous studies show that some proteins associated with Orb can either promote or repress its expression or activity. dFMR1 negatively regulates *orb* [14], whereas both *rin* and PABP function as positive regulators of *orb* expression and/or activity (this paper; [37]). To explore the functional significance of other proteins associated with both Orb and Rin, we tested several for genetic interactions with *orb* in the sensitized *Hd19G orb^343^/+* background. As shown in Figure S5, reducing *lig* activity greatly increases the frequency of ventralized eggs produced by females transheterozygous for *lig* and *Hd19G orb^343^*, similar to what was observed between *Hd19G orb^343^* and *rin*. In contrast, reducing *parp* activity suppresses the polarity defects induced by *Hd19G orb^343^*, as was observed for *dfmr1* [14]. As for *atx-2*, the allele we tested shows at most only a very modest interaction with *orb* (data not shown).

## Discussion

Previous studies have shown that *orb* autoregulation promotes localized accumulation of Orb protein in subcellular compartments where its activity is required [13]. To investigate the mechanisms underlying *orb* autoregulation we sought to identify proteins that physically associate with Orb and thus potentially help regulate its expression and/or activity. We have previously shown that the *Drosophila* Fragile-X protein (dFMR1) is found in complexes with Orb in *Drosophila* ovaries and functions to negatively regulate *orb* accumulation and activity [14]. Here we show that Rin, the *Drosophila* G3BP homologue, is also associated with Orb in ovaries. However, in contrast to dFMR1, Rin functions as a positive regulatory factor, helping to promote Orb accumulation and activity.

Our results show that Rin associates with Orb as part of an RNase-resistant complex. The RNase-resistance aspect of this interaction suggests that their association is mediated by protein-protein interactions rather than, or in addition to, binding to the same mRNA species. While it is possible that Orb and Rin interact directly with each other, an equally plausible scenario is that their association is mediated by one or more proteins found in both Orb and Rin immunoprecipitates. For example, mammalian G3BP has been shown to interact directly with Caprin-1, and the fly Caprin protein is found in both Rin and Orb immunoprecipitates. Two other findings would also seem to argue in favor of an indirect, rather than a direct interaction. First the overlap between Rin and Orb, especially in vitellogenic chambers is quite limited. Second, only a small subset of the proteins associated with Rin or Orb are common to both. It is also possible that the initial association between Orb and Rin could depend upon binding to the same target mRNAs and their subsequent interaction could depend upon a short stretch of RNA that is hidden in the complex and protected from RNase activity. In this case, the limited co-localization observed in egg chambers would imply that only a subset of their mRNA targets are in common.

GB3Ps in mammals are thought to have two functions. The first is repressing the translation of target mRNAs by mechanisms that depend upon their helicase and RNase activities, while the second is in the assembly of stress granules under conditions of environmental stress such as heat shock or drug treatment. For this reason, we anticipated that *rin*, like *dfmr1*, would function to negatively regulate *orb* activity and/or expression. However, we observed exactly the opposite result. Instead of suppressing the DV polarity defects in eggs from *Hd19G orb^343^/+* females, reducing *rin* activity increases the frequency of DV defects. Conversely, DV polarity defects are suppressed by providing excess *rin* activity. While it is clear from the phenotypic effects seen in mutants that *rin* has multiple functions in oogenesis (and these will be independent of *orb*), the most plausible explanation for the effects of decreasing and increasing *rin* activity on DV polarity in *Hd19G orb^343^/+* females is that they arise because of changes in the expression/accumulation of Orb protein. Consistent with this explanation, we find that we can change the level of Orb protein by manipulating *rin* activity. If *orb* is wild type, reducing the *rin* dose by half has little if any effect on Orb protein accumulation. However, when *orb* activity is compromised by the *Hd19G orb^343^* combination, heterozygosity for *rin* results in Orb protein levels that are less than 5% that of wild type. Conversely, increasing *rin* activity in a wild type background elevates Orb protein levels almost two-fold over wild type, while in background compromised by the *Hd19G orb^343^* combination adding extra *rin* restores Orb protein levels to that of wild type. Taken together, these findings argue that *rin* functions as a positive regulator of *orb*. At this point it is not clear how *rin* might control the accumulation of Orb protein. Since Rin and Orb are associated with each other, the simplest model is that Rin helps activate the translation of *orb* mRNA and thus functions as a co-factor in *orb* autoregulation. The substantial reduction in Orb protein levels evident in *rin^3^* ovaries is consistent with this idea. This view would also be supported by the DV polarity defects evident in eggs laid by *rin* mutant females. However, other less direct models (e.g., *rin* represses the translation of some factor that inhibits *orb* mRNA translation or *rin* is required to stabilize Orb protein) can't be excluded at this time. Likewise, *rin* may have other targets besides *orb* in the establishment of DV polarity.

Potentially arguing in favor of a role for Rin in the translation of *orb* mRNA and/or in the activity of Orb protein is the fact that mutations in genes encoding several of the other proteins found in both Orb and Rin immunoprecipitates also show genetic interactions with *orb*. Thus, *dfmr1* and *parp* suppress the DV polarity defects in eggs laid by *Hd19G orb^343^/+* females, while *pabp* and *lig* enhance the polarity defects. We have shown previously that *dfmr1* also exerts its effects, at least in part, by altering the expression of Orb protein [14]. Further connecting the effects of *rin* and *dfmr1* to translation, we also found that Orb, dFMR1 and Rin fractionate with polysomes in sucrose gradients (data not shown). If the proteins detected in Orb and Rin immunoprecipitates are part of the same complex rather than different complexes, then the gene dose effects we have observed would suggest that they act coordinately to regulate Orb expression and that the relative balance between positive (e.g., Rin) and negative (e.g., dFMR1) factors in the egg chamber helps set the level of Orb accumulation. Further studies will be required to understand precisely how *rin* influences Orb protein accumulation and how some of the other factors associated with Rin and Orb like dFMR1, Lig and PABP function in this process.

Although our studies implicate *rin* as a positive regulator of *orb*, this is clearly not the only role for *rin* in the ovary. Instead, the phenotypic effects of *rin* mutations point to a potentially diverse array of functions, not only in gem cells but also in the surrounding somatic follicle cells. For example, the fragmentation of ring canals, the failure to properly disperse the endoreplicated nurse cell chromosomes, and the dumpless phenotype are not observed in *orb* mutants. Moreover, ring canal and chromatin dispersal defects suggest that *rin* has functions in nurse cells, which is a compartment that has only little Orb protein. Likewise, the encapsulation defects could be of somatic origin where *rin*, but not *orb* is expressed. Further studies will be required to identify the *rin* regulatory targets in these and potentially other processes.

## Materials and Methods

### Fly stocks and genetic analysis

Wild type *w^1118^* and *orb^343^* have been described previously [1] as have the *rin^1^ and rin^2^* mutant stocks and *Tub-rin* rescue construct [34]. The *HD19G* transgene containing DNA sequences from the *orb* 3′UTR has been described in [38].

3–5 day old females of various genotypes were mated to *w^1118^* males in cages at 18°C, 25°C or 29°C. Beginning on the second day, eggs were collected for seven days and at least 200 eggs/day were scored for a ventralized phenotype as described in [13].

### In situ hybridization

Whole-mount in-situ hybridization was performed as described [14] on wild type ovaries. DIG-labeled *rin* cDNA was used as a probe and was labeled according to the manufacturer's instructions (Roche).

### Immunostaining

Ovaries were dissected in ice-cold 1× PBS and ovarioles microdissected with needles. Ovarioles were fixed with 4% paraformaldehyde in 1× PBS for 20 minutes at room temperature followed by three washes in 1× PBS. Ovarioles were rinsed once in PBST (0.1% Triton X-100 in 1× PBS) and blocked in PBSTTB (0.1% Triton X-100, 0.05% Tween 20, 10% BSA in 1× PBS) for 1–2 hours at room temperature. Labeling was performed with anti-Orb antibodies (6H4 obtained from the Developmental Studies Hybridoma Bank) at 1∶30 and anti-Rin antibodies [34] at 1∶1000 in 1× PBSTTB overnight at 4°C. After several washes in 1× PBSTT, ovarioles were incubated for 2 hours in 1× PBSTTB with Alexa Fluor 568 goat anti-mouse IgG_2a_ (Molecular Probes, Inc.) against Orb 6H4 antibodies, and Alexa Fluor 647 goat anti-rabbit (Molecular Probes, Inc.) against Rin antibodies. After several washes in 1× PBSTT, ovarioles were mounted in 15–30% glycerol in 1× PBS and visualized by confocal microscopy. Microscopy was performed with an inverted Zeiss LSM510 confocal microscope.

### Immunoprecipitation and western analysis

Ovaries of well-fed 1- to 4-day old wild-type females were hand-dissected in ice-cold 1× PBS, frozen in dry ice and stored at −80°C. Ovary extracts were prepared by homogenizing 100 ovaries in 200 µl of ice-cold IP buffer (20 mM Hepes, pH 7.5, 150 mM NaCl, 2.5 mM MgCl_2_, 250 mM sucrose, 0.05% Tergitol, 0.5% Triton X-100, 1 µg/ml pepstatin A, 10 µg/ml aprotinin, 1 µg/ml leupeptin, 1 mM dithiothreitol, 1 mM PMSF, 1 mM NaF, 40 µM NaVO_3_, 40 µM Na_3_VO_4_) supplemented with 500 µg of RNase A. Homogenates were cleared twice by centrifugation at 750 *g* for 5 minutes at 4°C and supernatants were transferred to a fresh microfuge tube. Supernatants were diluted to 500 µl with ice-cold IP buffer and mixed with 60 µl of a 50% slurry of Protein G Plus/Protein A-agarose beads (Calbiochem) to which antibodies have been crosslinked at 2 mg/ml. After overnight rotation at 4°C, beads were precipitated by centrifugation at 100 *g* for 30 seconds and washed 4 times with 100 volumes of ice-cold IP buffer without MgCl_2_.

For western blotting, 5 µl of washed beads (Figure 1) or 20 µg of total protein (Figure 8B) were electrophoresed through an 11% SDS-polyacrylamide gel, transferred onto Immobilon-P PVDF membranes (Millipore), and probed with anti-Orb antibodies (6H4 and 4H8 obtained from the Developmental Studies Hybridoma Bank) at 1∶30 or anti-α-tubulin antibodies (Sigma) at 1∶2500 followed by secondary peroxidase-conjugated goat anti-mouse antibodies (Jackson ImmunoResearch Laboratories) at 1∶2000. Membranes were also probed with anti-Rin antibodies [34] at 1∶3000 followed by secondary peroxidase-conjugated goat anti-rabbit antibodies (Jackson ImmunoResearch Laboratories) at 1∶2000. Proteins were detected by chemiluminescence according to manufacturer's instructions (ECL, Amersham Pharmacia Biotech).

For the western blots shown in Figure 3B, ovaries or female carcasses were homogenized in RIPA buffer and the extract was quantitated using the Bio-Rad protein assay (Bio-Rad Laboratories) to ensure equal amounts of sample per lane. After separation on 4–12% polyacrylamide gels, proteins were transferred to ECL-nitrocellulose (Amersham Pharmacia Biotech), and probed with anti-Rin antibodies as described above.

### Protein Identification by Tandem Mass Spectrometry

MS/MS spectra were analyzed using the following software analysis protocol. Poor quality spectra were removed from the dataset using an automated spectral quality assessment algorithm [39]. MS/MS spectra remaining after filtering were searched with the SEQUEST™ algorithm [40] against the NCBI, RefSeq Drosophila, 6/2004. All searches were parallelized and performed on a Beowulf computer cluster consisting of 100 1.2 GHz Athlon CPUs. No enzyme specificity was considered for any search. SEQUEST results were assembled and filtered using the DTASelect (version 1.9) program [41].

## Supporting Information

Figure S1 **A) Rin Immunoprecipates.** Ovary extracts were immunoprecipitated with b-Gal or Rin antibodies. In the top blot, the immunoprecipitate (IP) was probed with Orb antibody. In the bottom blot the immunoprecipitate was probed with Rin antibody. **B) Westerns of** ***rin^3^*** **mutant ovaries.** Western blots of ovary extracts from wild type (WT) or *rin^3^* mutant ovaries were probed as indicated.
(TIF)

Figure S2 **Effects of** ***rin*** **mutations on lifespan.** Newly emerged adult flies of each genotype were collected, placed in fresh vials with normal yeast cornmeal media (10 flies per vial and 10 vials of each genotype), and monitored for survival every 3 days for a period of 40 days at 25°C. The graph shows the average of two experiments. Heterozygotes were indistinguishable from wild type.
(TIF)

Figure S3 **A subset of the** ***rin^3^*** **chambers have the dumpless phenotype.** Wild type (A) and *rin^3^* (B) egg chambers. As illustrated in B) a subset (5–10%) of the late stage *rin* ^3^ chambers are dumpless. Arrow marks the dorsal appendages.
(TIF)

Figure S4 **Rescue of** ***rin^3^*** **oogenesis defects by the** ***Tub-rin*** **transgene.** The *Tub-rin* transgene not only rescues fertility, but also fully rescues the partitioning, nuclear structure and ring canal defects of *rin^3^* (*Tub-rin*; *rin^3^/rin^3^* females). A) DAPI staining showing rescue of the nuclear chromosomal phenotype. B) Actin staining shows rescue of the partitioning defects. C) Phosphotyrosine antibody shows rescue of the ring canal defects.
(TIF)

Figure S5 **Genetic interactions between** ***orb*** **and genes encoding proteins common to Orb and Rin immunoprecipitations.** The percentage of dorsal-ventral polarity defects in eggs laid at 25^p^ C by females *trans*-heterozygous for *Hd19G orb^343^* (*orb*) and genes (*lig*, *parp*, and *atx-2*) encoding proteins detected in both Orb and Rin immunoprecipitates is shown. Fused dorsal appendage phenotypes range from fusion at the base to fusion along the entire length of the two appendages. *Hd19G* is a dominant negative transgene carrying sequences of the *orb* 3′UTR bound by endogenous Orb and sufficient to recapitulate the pattern of localization of the endogenous *orb* transcript [13]; *orb^343^*, *orb* null allele [2]. *Hd19G orb^343^/atx-2* results are from a different experiment and the frequency of DV polarity defects in the control *Hd19G orb^343^/+* females is less that seen in other control experiments. Even so the effects of *atx-2* are modest.
(TIF)

Table S1 **Viability of flies carrying** ***rin*** **mutations.** Percent of viability is calculated as the number of observed/number of expected *rin* mutant flies (n). The observed number of *rin/+* siblings was used to calculate the number of expected *rin* mutant flies. *Df(3R)urd* deletes *rin* and adjacent loci.
(DOC)

Table S2 **Proteins detected in Dorsal, Orb and Rin immunoprecipitates from ovary extracts.** At least two peptides were detected for all proteins on the list.
(DOC)

Table S3 **Potentially relevant proteins present in Orb, Rin and also Dorsal Immunoprecipitates.** In addition to ribosomal proteins, a number of these proteins have been found associated with Rin homologs in other species or have been implicated in translation regulation.
(DOC)
