# Supplementary figures and images for: Rasputin Functions as a Positive Regulator of Orb in Drosophila Oogenesis
Source: PLoS One. 2013 Sep 12;8(9):e72864. doi: 10.1371/journal.pone.0072864 (PMC3771913; doi:10.1371/journal.pone.0072864)

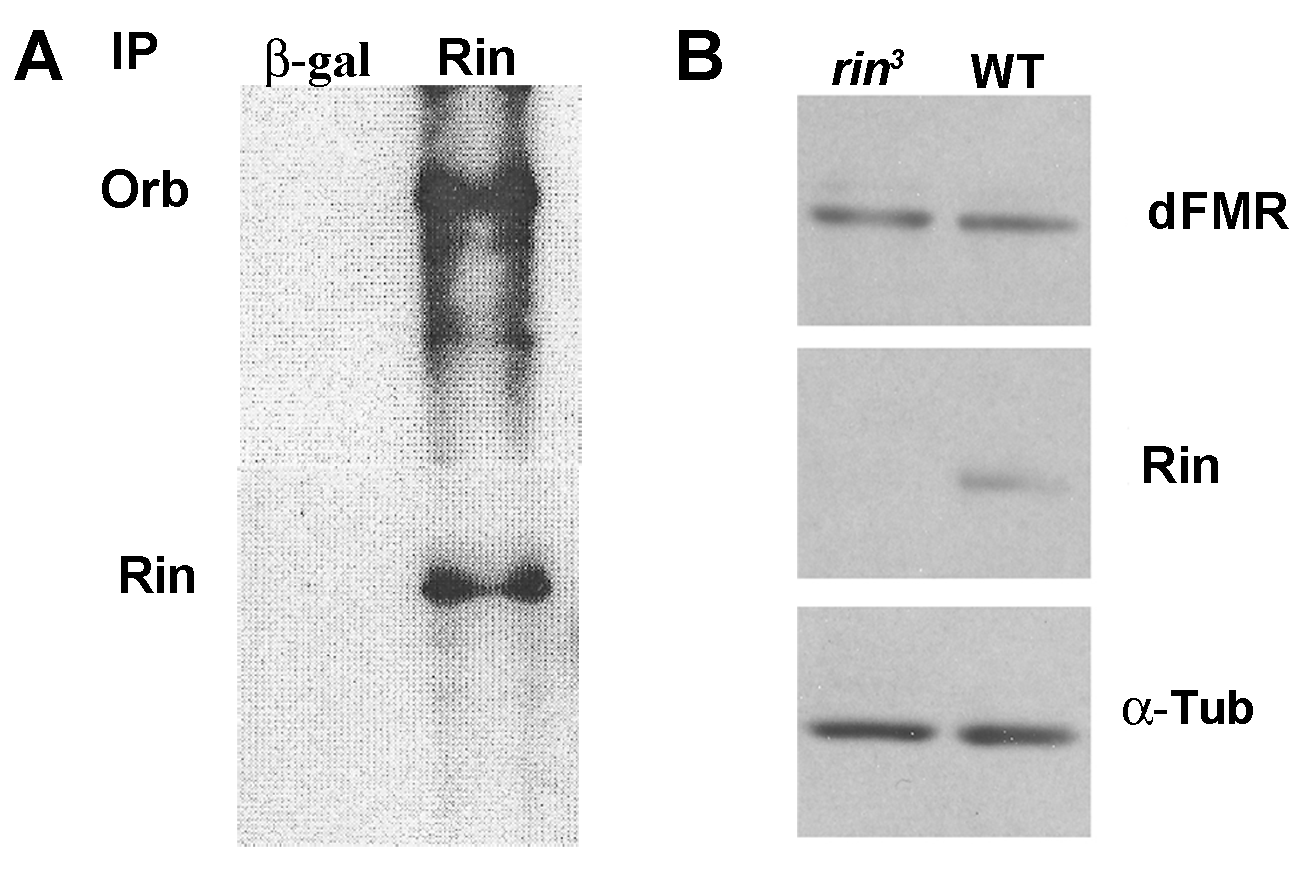

Supplement: Figure S1 — A) Rin Immunoprecipates. Ovary extracts were immunoprecipitated with b-Gal or Rin antibodies. In the top blot, the immunoprecipitate (IP) was probed with Orb antibody. In the bottom blot the immunoprecipitate was probed with Rin antibody. B) Westerns of rin3 mutant ovaries. Western blots of ovary extracts from wild type (WT) or rin3 mutant ovaries were probed as indicated. (TIF) [file pone.0072864.s001.tif]

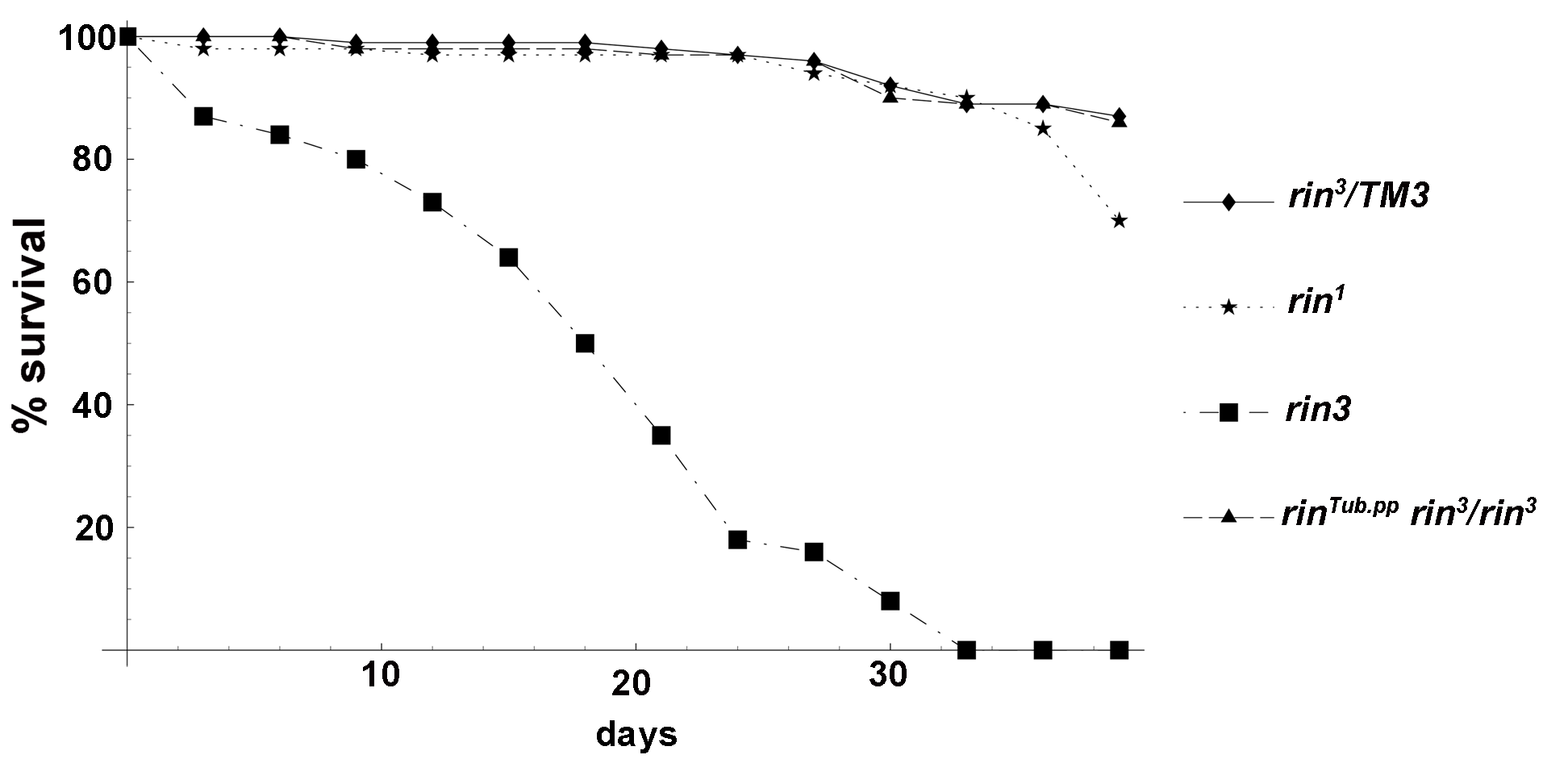

Supplement: Figure S2 — Effects of rin mutations on lifespan. Newly emerged adult flies of each genotype were collected, placed in fresh vials with normal yeast cornmeal media (10 flies per vial and 10 vials of each genotype), and monitored for survival every 3 days for a period of 40 days at 25°C. The graph shows the average of two experiments. Heterozygotes were indistinguishable from wild type. (TIF) [file pone.0072864.s002.tif]

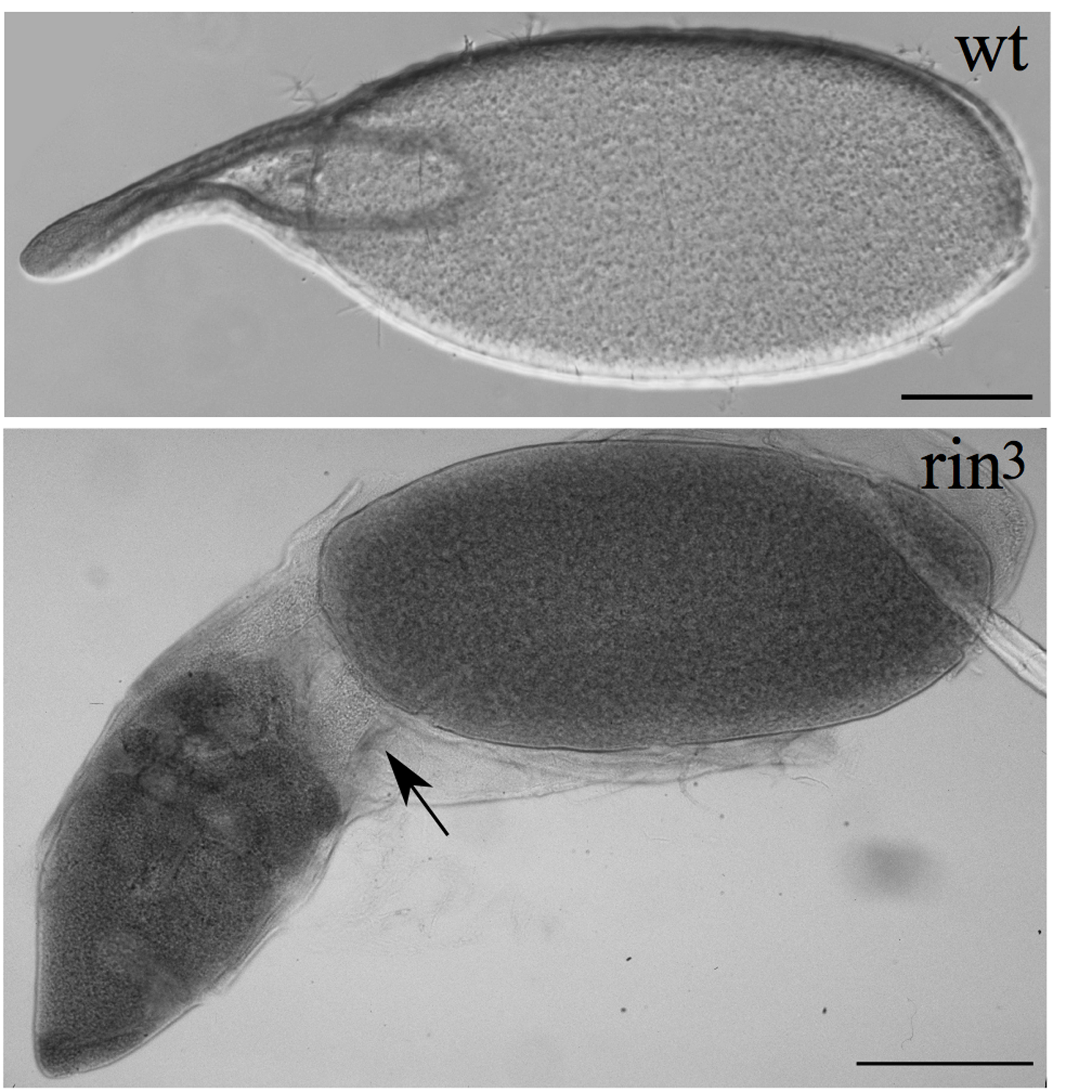

Supplement: Figure S3 — A subset of the rin3 chambers have the dumpless phenotype. Wild type (A) and rin3 (B) egg chambers. As illustrated in B) a subset (5–10%) of the late stage rin 3 chambers are dumpless. Arrow marks the dorsal appendages. (TIF) [file pone.0072864.s003.tif]

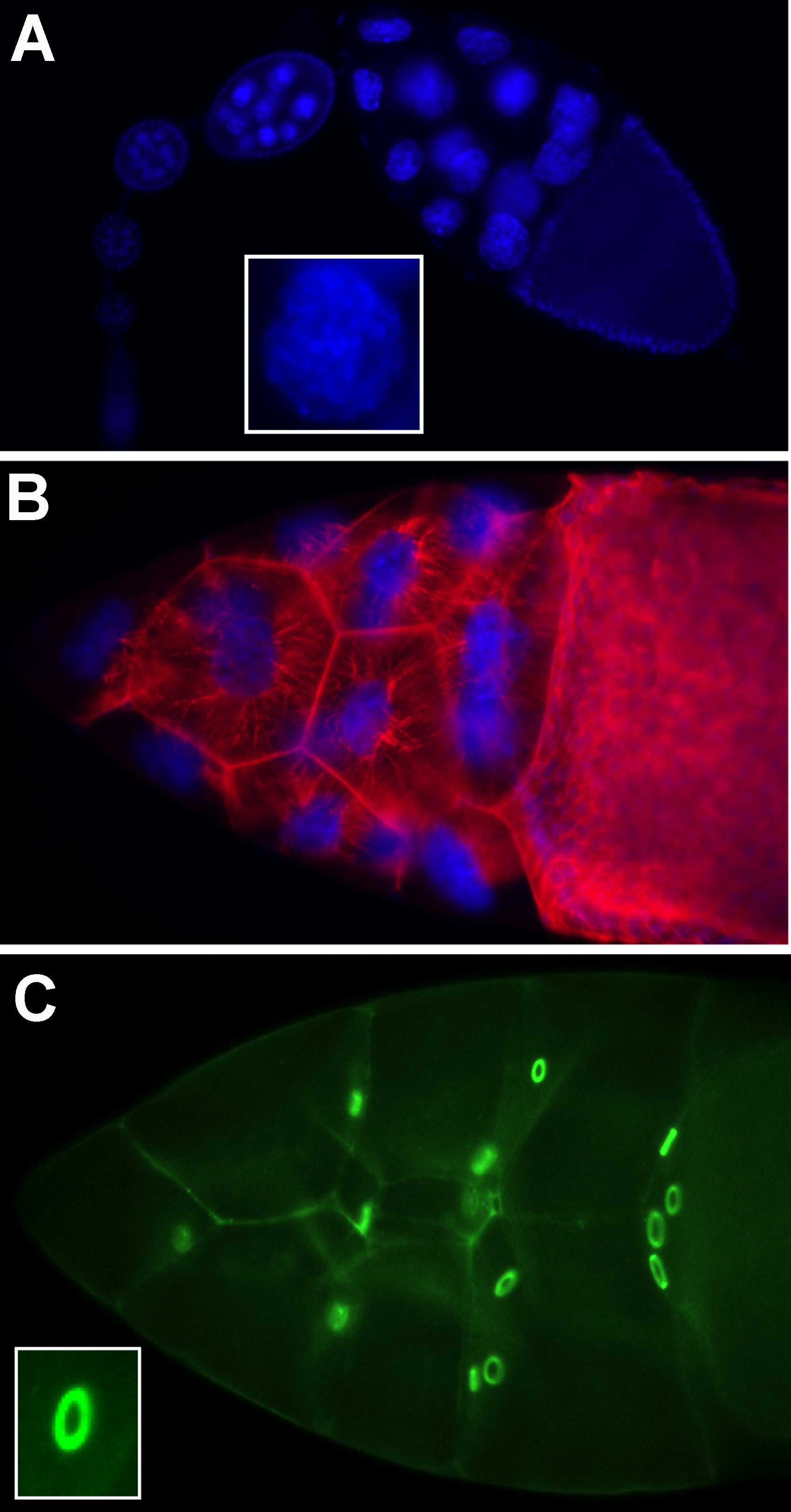

Supplement: Figure S4 — Rescue of rin3 oogenesis defects by the Tub-rin transgene. The Tub-rin transgene not only rescues fertility, but also fully rescues the partitioning, nuclear structure and ring canal defects of rin3 (Tub-rin; rin3/rin3 females). A) DAPI staining showing rescue of the nuclear chromosomal phenotype. B) Actin staining shows rescue of the partitioning defects. C) Phosphotyrosine antibody shows rescue of the ring canal defects. (TIF) [file pone.0072864.s004.tif]

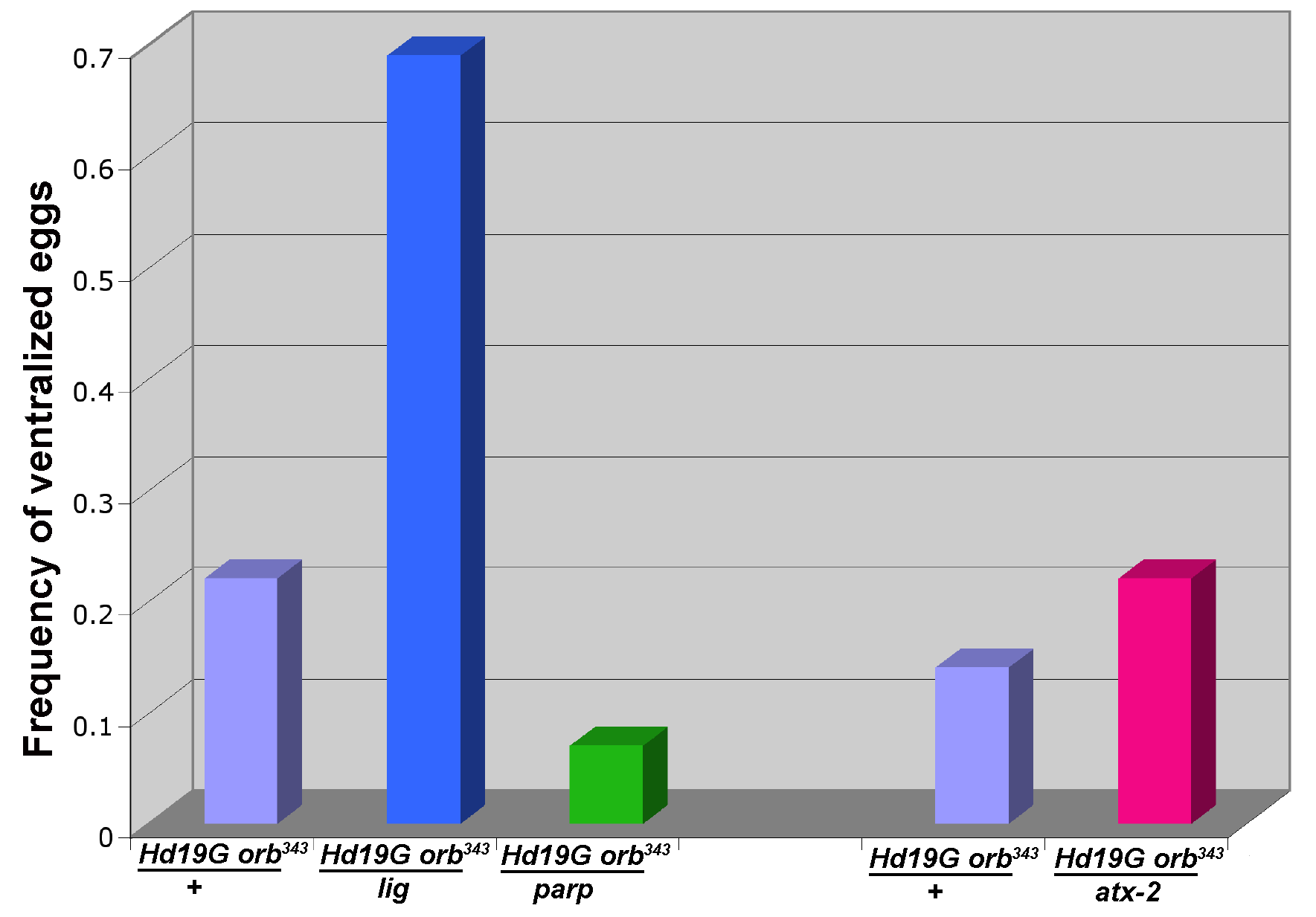

Supplement: Figure S5 — Genetic interactions between orb and genes encoding proteins common to Orb and Rin immunoprecipitations. The percentage of dorsal-ventral polarity defects in eggs laid at 25p C by females trans-heterozygous for Hd19G orb343 (orb) and genes (lig, parp, and atx-2) encoding proteins detected in both Orb and Rin immunoprecipitates is shown. Fused dorsal appendage phenotypes range from fusion at the base to fusion along the entire length of the two appendages. Hd19G is a dominant negative transgene carrying sequences of the orb 3′UTR bound by endogenous Orb and sufficient to recapitulate the pattern of localization of the endogenous orb transcript [13]; orb343, orb null allele [2]. Hd19G orb343/atx-2 results are from a different experiment and the frequency of DV polarity defects in the control Hd19G orb343/+ females is less that seen in other control experiments. Even so the effects of atx-2 are modest. (TIF) [file pone.0072864.s005.tif]
