# Supplementary material for: Rasputin Functions as a Positive Regulator of Orb in Drosophila Oogenesis
Source: PLoS One. 2013 Sep 12;8(9):e72864. doi: 10.1371/journal.pone.0072864 (PMC3771913; doi:10.1371/journal.pone.0072864)
Supplement: Table S1 — Viability of flies carrying rin mutations. Percent of viability is calculated as the number of observed/number of expected rin mutant flies (n). The observed number of rin/+ siblings was used to calculate the number of expected rin mutant flies. Df(3R)urd deletes rin and adjacent loci. (DOC) [file pone.0072864.s006.doc]

| Genotype % Viability n |
| --- |

*rin^1^*/*Df(3R)urd* 68 2266 / 3314

*rin^1^*/*rin^3^* 76 834 / 1102

*rin^3^*/*rin^3^* 62 734 / 1180

*rin^3^*/*Df(3R)urd*  60 713 / 1188

*rin^Tub-rin^ rin^3^/rin^3^* 89 463 / 520

**Table S1.** **Viability of flies carrying *rin* mutations.**
