# Supplementary material for: Rasputin Functions as a Positive Regulator of Orb in Drosophila Oogenesis
Source: PLoS One. 2013 Sep 12;8(9):e72864. doi: 10.1371/journal.pone.0072864 (PMC3771913; doi:10.1371/journal.pone.0072864)
Supplement: Table S2 — Proteins detected in Dorsal, Orb and Rin immunoprecipitates from ovary extracts. At least two peptides were detected for all proteins on the list. (DOC) [file pone.0072864.s007.doc]

**Table S2. Proteins detected in Dorsal, Orb and Rin immunoprecipitates from ovary extracts.**

| ***Locus*** | ***dorsal*** | ***orb*** | ***rin*** | ***Total*** | ***Description*** |
| --- | --- | --- | --- | --- | --- |
| 7LES_DROME |  |  | [0.3](http://molbio.princeton.edu/exchange/Attach/rin-x.html#7LES_DROME) | [0.3](http://gemini.scripps.edu/cgi-bin/SeqCov?/wfs/bfd/3/ian/fly/DrosMelano7227.FASTAC.txt&7LES_DROME&112+7&113+5&114+3*) | (P13368) Sevenless protein (EC 2.7.1.112) |
| CH6C_DROME |  |  | [13.0](http://molbio.princeton.edu/exchange/Attach/rin-x.html#CH6C_DROME) | [13.0](http://gemini.scripps.edu/cgi-bin/SeqCov?/wfs/bfd/3/ian/fly/DrosMelano7227.FASTAC.txt&CH6C_DROME&38+21^97+25^221+29&38+21*) | (Q9VMN5) Probable 60 kDa heat shock protein homolog 2, mitochondrial precursor (Hsp60) (60 kDa chaperonin) (CPN60) (Heat shock protein 60) (HSP-60) |
| CYA1_DROME  Q9VR90  Q9VNT7  INL3_DROME |  |  | [0.3](http://molbio.princeton.edu/exchange/Attach/rin-x.html#CYA1_DROME) | [0.3](http://gemini.scripps.edu/cgi-bin/SeqCov?/wfs/bfd/3/ian/fly/DrosMelano7227.FASTAC.txt&CYA1_DROME&712+6&713+4*)  [1.7](http://gemini.scripps.edu/cgi-bin/SeqCov?/wfs/bfd/3/ian/fly/DrosMelano7227.FASTAC.txt&Q9VR90&344+6&345+4*)  [1.1](http://gemini.scripps.edu/cgi-bin/SeqCov?/wfs/bfd/3/ian/fly/DrosMelano7227.FASTAC.txt&Q9VNT7&349+6&350+4*)  [4.8](http://gemini.scripps.edu/cgi-bin/SeqCov?/wfs/bfd/3/ian/fly/DrosMelano7227.FASTAC.txt&INL3_DROME&17+6&18+4*) | (P32870) Ca(2+)/calmodulin-responsive adenylate cyclase (EC 4.6.1.1) (ATP pyrophosphate-lyase) (Rutabaga protein)  (Q9VR90) CG1467 protein  (Q9VNT7) CG12377 protein  (Q9VT52) Probable insulin-like peptide 3 precursor |
| G3P1_DROME  G3P2_DROME |  |  | [11.4](http://molbio.princeton.edu/exchange/Attach/rin-x.html#G3P1_DROME) | [11.4](http://gemini.scripps.edu/cgi-bin/SeqCov?/wfs/bfd/3/ian/fly/DrosMelano7227.FASTAC.txt&G3P1_DROME&160+24^307+14*)  [11.4](http://gemini.scripps.edu/cgi-bin/SeqCov?/wfs/bfd/3/ian/fly/DrosMelano7227.FASTAC.txt&G3P2_DROME&160+24^307+14*) | (P07486) Glyceraldehyde 3-phosphate dehydrogenase I (EC 1.2.1.12) (GAPDH I)  (P07487) Glyceraldehyde 3-phosphate dehydrogenase II (EC 1.2.1.12) (GAPDH II) |
| NU4M_DROME |  |  | [2.2](http://molbio.princeton.edu/exchange/Attach/rin-x.html#NU4M_DROME) | [2.2](http://gemini.scripps.edu/cgi-bin/SeqCov?/wfs/bfd/3/ian/fly/DrosMelano7227.FASTAC.txt&NU4M_DROME&93+10&97+6&98+4*) | (P18931) NADH-ubiquinone oxidoreductase chain 4 (EC 1.6.5.3) |
| O85F_DROME |  |  | [1.5](http://molbio.princeton.edu/exchange/Attach/rin-x.html#O85F_DROME) | [1.5](http://gemini.scripps.edu/cgi-bin/SeqCov?/wfs/bfd/3/ian/fly/DrosMelano7227.FASTAC.txt&O85F_DROME&347+6&348+4*) | (Q9VHE6) Putative odorant receptor 85f |
| PYR1_DROME |  |  | [1.8](http://molbio.princeton.edu/exchange/Attach/rin-x.html#PYR1_DROME) | [1.8](http://gemini.scripps.edu/cgi-bin/SeqCov?/wfs/bfd/3/ian/fly/DrosMelano7227.FASTAC.txt&PYR1_DROME&232+29^1332+11*) | (P05990) CAD protein (Rudimentary protein) [Includes: Glutamine-dependent carbamoyl-phosphate synthase (EC 6.3.5.5); Aspartate carbamoyltransferase (EC 2.1.3.2); Dihydroorotase (EC 3.5.2.3)] |
| Q7PLN5 |  |  | [12.6](http://molbio.princeton.edu/exchange/Attach/rin-x.html#Q7PLN5) | [12.6](http://gemini.scripps.edu/cgi-bin/SeqCov?/wfs/bfd/3/ian/fly/DrosMelano7227.FASTAC.txt&Q7PLN5&46+15^98+18^121+36*) | (Q7PLN5) CG40084-PC.3 |
| Q8IRB9  Q9VZI0  Q9I7T2  Q8IRC1  Q8IRC0 |  |  | [16.1](http://molbio.princeton.edu/exchange/Attach/rin-x.html#Q8IRB9) | [16.1](http://gemini.scripps.edu/cgi-bin/SeqCov?/wfs/bfd/3/ian/fly/DrosMelano7227.FASTAC.txt&Q8IRB9&134+13^168+14^355+19^384+16^424+17^494+35^574+25^871+18&574+25*)  [18.6](http://gemini.scripps.edu/cgi-bin/SeqCov?/wfs/bfd/3/ian/fly/DrosMelano7227.FASTAC.txt&Q9VZI0&134+13^168+14^223+19^252+16^292+17^362+35^442+25^739+18&442+25*)  [17.8](http://gemini.scripps.edu/cgi-bin/SeqCov?/wfs/bfd/3/ian/fly/DrosMelano7227.FASTAC.txt&Q9I7T2&170+13^204+14^259+19^288+16^328+17^398+35^478+25^775+18&478+25*)  [19.0](http://gemini.scripps.edu/cgi-bin/SeqCov?/wfs/bfd/3/ian/fly/DrosMelano7227.FASTAC.txt&Q8IRC1&116+13^150+14^205+19^234+16^274+17^344+35^424+25^721+18&424+25*)  [17.6](http://gemini.scripps.edu/cgi-bin/SeqCov?/wfs/bfd/3/ian/fly/DrosMelano7227.FASTAC.txt&Q8IRC0&134+13^168+14^270+19^299+16^339+17^409+35^489+25^786+18&489+25*) | (Q8IRB9) CG14998-PD  (Q9VZI0) CG14998 protein  (Q9I7T2) CG14998 protein  (Q8IRC1) CG14998-PC  (Q8IRC0) CG14998-PE |
| Q9TW27  Q9W5S7  Q9U4F5 |  |  | [15.1](http://molbio.princeton.edu/exchange/Attach/rin-x.html#Q9TW27) | [15.1](http://gemini.scripps.edu/cgi-bin/SeqCov?/wfs/bfd/3/ian/fly/DrosMelano7227.FASTAC.txt&Q9TW27&108+31^289+31*)  [15.2](http://gemini.scripps.edu/cgi-bin/SeqCov?/wfs/bfd/3/ian/fly/DrosMelano7227.FASTAC.txt&Q9W5S7&107+31^288+31*)  [15.7](http://gemini.scripps.edu/cgi-bin/SeqCov?/wfs/bfd/3/ian/fly/DrosMelano7227.FASTAC.txt&Q9U4F5&93+31^274+31*) | (Q9TW27) DISCO-interacting protein (CG17686-PD) (DsRBD-containing protein dip1-c)  (Q9W5S7) CG17686 protein (CG17686-PB)  (Q9U4F5) DISCO-interacting protein (CG17686-PC) (DsRBD-containing protein dip1-b) |
| Q9V4G9 |  |  | [4.1](http://molbio.princeton.edu/exchange/Attach/rin-x.html#Q9V4G9) | [4.1](http://gemini.scripps.edu/cgi-bin/SeqCov?/wfs/bfd/3/ian/fly/DrosMelano7227.FASTAC.txt&Q9V4G9&77+9&78+5&81+1*) | (Q9V4G9) CG12840 protein (Tetraspanin 42El) (GH14950p) |
| Q9V7Y9  Q9VV49 |  |  | [1.8](http://molbio.princeton.edu/exchange/Attach/rin-x.html#Q9V7Y9) | [1.8](http://gemini.scripps.edu/cgi-bin/SeqCov?/wfs/bfd/3/ian/fly/DrosMelano7227.FASTAC.txt&Q9V7Y9&4+6&5+4*)  [1.6](http://gemini.scripps.edu/cgi-bin/SeqCov?/wfs/bfd/3/ian/fly/DrosMelano7227.FASTAC.txt&Q9VV49&28+6&29+4*) | (Q9V7Y9) CG10956 protein  (Q9VV49) CG4753 protein (RE72803p) |
| Q9V8M3 |  |  | [10.3](http://molbio.princeton.edu/exchange/Attach/rin-x.html#Q9V8M3) | [10.3](http://gemini.scripps.edu/cgi-bin/SeqCov?/wfs/bfd/3/ian/fly/DrosMelano7227.FASTAC.txt&Q9V8M3&18+15^204+18*) | (Q9V8M3) CG15092 protein (LD47410p) |
| Q9VAS8 |  |  | [2.1](http://molbio.princeton.edu/exchange/Attach/rin-x.html#Q9VAS8) | [2.1](http://gemini.scripps.edu/cgi-bin/SeqCov?/wfs/bfd/3/ian/fly/DrosMelano7227.FASTAC.txt&Q9VAS8&335+27^390+18*) | (Q9VAS8) CG31048-PA |
| Q9VHJ8 |  |  | [7.4](http://molbio.princeton.edu/exchange/Attach/rin-x.html#Q9VHJ8) | [7.4](http://gemini.scripps.edu/cgi-bin/SeqCov?/wfs/bfd/3/ian/fly/DrosMelano7227.FASTAC.txt&Q9VHJ8&236+24^421+13*) | (Q9VHJ8) CG11963-PA |
| Q9VHL2 |  |  | [13.4](http://molbio.princeton.edu/exchange/Attach/rin-x.html#Q9VHL2) | [13.4](http://gemini.scripps.edu/cgi-bin/SeqCov?/wfs/bfd/3/ian/fly/DrosMelano7227.FASTAC.txt&Q9VHL2&10+27^199+18^265+12^447+16*) | (Q9VHL2) CG8351 protein (LD47396p) |
| Q9VKH9 |  |  | [7.1](http://molbio.princeton.edu/exchange/Attach/rin-x.html#Q9VKH9) | [7.1](http://gemini.scripps.edu/cgi-bin/SeqCov?/wfs/bfd/3/ian/fly/DrosMelano7227.FASTAC.txt&Q9VKH9&33+30^544+29^637+18^691+16^1447+15^1610+12^1646+17*) | (Q9VKH9) Cana protein |
| Q9VXQ1 |  |  | [2.7](http://molbio.princeton.edu/exchange/Attach/rin-x.html#Q9VXQ1) | [2.7](http://gemini.scripps.edu/cgi-bin/SeqCov?/wfs/bfd/3/ian/fly/DrosMelano7227.FASTAC.txt&Q9VXQ1&4+11&5+9*) | (Q9VXQ1) CG8260 protein |
| Q9VXQ5 |  |  | [12.9](http://molbio.princeton.edu/exchange/Attach/rin-x.html#Q9VXQ5) | [12.9](http://gemini.scripps.edu/cgi-bin/SeqCov?/wfs/bfd/3/ian/fly/DrosMelano7227.FASTAC.txt&Q9VXQ5&16+13^160+25^437+31*) | (Q9VXQ5) CG8231 protein (GH13725P) |
| Q9VYR0 |  |  | [18.9](http://molbio.princeton.edu/exchange/Attach/rin-x.html#Q9VYR0) | [18.9](http://gemini.scripps.edu/cgi-bin/SeqCov?/wfs/bfd/3/ian/fly/DrosMelano7227.FASTAC.txt&Q9VYR0&54+23^156+18*) | (Q9VYR0) CG15735 protein (LD05893p) |
| Q9XZ14 |  |  | [1.1](http://molbio.princeton.edu/exchange/Attach/rin-x.html#Q9XZ14) | [1.1](http://gemini.scripps.edu/cgi-bin/SeqCov?/wfs/bfd/3/ian/fly/DrosMelano7227.FASTAC.txt&Q9XZ14&139+10&141+5*) | (Q9XZ14) BCDNA:LD21405 protein |
| TCPA_DROME |  |  | [17.2](http://molbio.princeton.edu/exchange/Attach/rin-x.html#TCPA_DROME) | [17.2](http://gemini.scripps.edu/cgi-bin/SeqCov?/wfs/bfd/3/ian/fly/DrosMelano7227.FASTAC.txt&TCPA_DROME&47+20^77+29^163+21^411+26*) | (P12613) T-complex protein 1, alpha subunit (TCP-1-alpha) (CCT-alpha) |
| **22** |  |  | **X** |  |  |

| ***Locus*** | ***dorsal*** | ***orb*** | ***rin*** | ***Total*** | ***Description*** |
| --- | --- | --- | --- | --- | --- |
| 143E_DROME |  | [11.8](http://molbio.princeton.edu/exchange/Attach/orb-x.html#143E_DROME) |  | [11.8](http://gemini.scripps.edu/cgi-bin/SeqCov?/wfs/bfd/3/ian/fly/DrosMelano7227.FASTAC.txt&143E_DROME&95+12^197+19*) | (P92177) 14-3-3 protein epsilon (Suppressor of Ras1 3-9) |
| AAF48000  Q9VZ30 |  | [1.0](http://molbio.princeton.edu/exchange/Attach/orb-x.html#AAF48000) |  | [1.0](http://gemini.scripps.edu/cgi-bin/SeqCov?/wfs/bfd/3/ian/fly/DrosMelano7227.FASTAC.txt&AAF48000&1372+13^2765+24*)  [1.0](http://gemini.scripps.edu/cgi-bin/SeqCov?/wfs/bfd/3/ian/fly/DrosMelano7227.FASTAC.txt&Q9VZ30&1369+13^2762+24*) | (AAF48000) CG11122-PA  (Q9VZ30) CG11122 protein |
| AAF51926  RM62_DROME |  | [25.4](http://molbio.princeton.edu/exchange/Attach/orb-x.html#AAF51926) |  | [25.4](http://gemini.scripps.edu/cgi-bin/SeqCov?/wfs/bfd/3/ian/fly/DrosMelano7227.FASTAC.txt&AAF51926&123+10^162+24^191+53^274+13^377+15^460+17^510+15&191+20&195+16*)  [20.4](http://gemini.scripps.edu/cgi-bin/SeqCov?/wfs/bfd/3/ian/fly/DrosMelano7227.FASTAC.txt&RM62_DROME&264+10^303+24^332+53^415+13^518+15^601+17^651+15&332+20&336+16*) | (AAF51926) CG10279-PC  (P19109) Putative ATP-dependent RNA helicase P62 |
| AAF54180  Q9VHW9  AAG22133 |  | [2.8](http://molbio.princeton.edu/exchange/Attach/orb-x.html#AAF54180) |  | [2.8](http://gemini.scripps.edu/cgi-bin/SeqCov?/wfs/bfd/3/ian/fly/DrosMelano7227.FASTAC.txt&AAF54180&22+10^163+14^1424+11^1660+12^1958+12*)  [2.8](http://gemini.scripps.edu/cgi-bin/SeqCov?/wfs/bfd/3/ian/fly/DrosMelano7227.FASTAC.txt&Q9VHW9&22+10^163+14^1424+11^1660+12^1958+12*)  [2.7](http://gemini.scripps.edu/cgi-bin/SeqCov?/wfs/bfd/3/ian/fly/DrosMelano7227.FASTAC.txt&AAG22133&70+10^211+14^1472+11^1708+12^2006+12*) | (AAF54180) CG2747-PB  (Q9VHW9) CG2747 protein  (AAG22133) CG2747-PA |
| AAF54303 |  | [3.1](http://molbio.princeton.edu/exchange/Attach/orb-x.html#AAF54303) |  | [3.1](http://gemini.scripps.edu/cgi-bin/SeqCov?/wfs/bfd/3/ian/fly/DrosMelano7227.FASTAC.txt&AAF54303&80+25^370+11*) | (AAF54303) CG31349-PE |
| AAF54520 |  | [11.8](http://molbio.princeton.edu/exchange/Attach/orb-x.html#AAF54520) |  | [11.8](http://gemini.scripps.edu/cgi-bin/SeqCov?/wfs/bfd/3/ian/fly/DrosMelano7227.FASTAC.txt&AAF54520&89+12^207+14^450+16^1085+19^1434+22^1840+19^2114+10^2346+53^2634+22^2821+26^2863+35^3005+13^3195+22^3237+21^3263+37^3311+12^3380+36^3633+19^3680+16^3717+26^3782+30^4064+41^4157+17^4257+21^4624+16&2385+14^3680+16*) | (AAF54520) CG6303-PA |
| AAF55259  Q9VF03 |  | [25.2](http://molbio.princeton.edu/exchange/Attach/orb-x.html#AAF55259) |  | [25.2](http://gemini.scripps.edu/cgi-bin/SeqCov?/wfs/bfd/3/ian/fly/DrosMelano7227.FASTAC.txt&AAF55259&51+17^124+34^171+28^280+13^501+13^515+11^550+25^611+22^685+11^723+19^749+23^782+25^889+18^938+23^974+12^993+11&51+17^125+21^172+27^558+17^611+22^723+19^749+23^938+23*)  [24.8](http://gemini.scripps.edu/cgi-bin/SeqCov?/wfs/bfd/3/ian/fly/DrosMelano7227.FASTAC.txt&Q9VF03&70+17^143+34^190+28^299+13^520+13^534+11^569+25^630+22^704+11^742+19^768+23^801+25^908+18^957+23^993+12^1012+11&70+17^144+21^191+27^577+17^630+22^742+19^768+23^957+23*) | (AAF55259) CG18740-PA  (Q9VF03) MOIRA protein (Brahma associated protein 155 kDa) (Brahma-associated putative chromatin-remodeling factor) |
| AAM68752 |  | [30.8](http://molbio.princeton.edu/exchange/Attach/orb-x.html#AAM68752) |  | [30.8](http://gemini.scripps.edu/cgi-bin/SeqCov?/wfs/bfd/3/ian/fly/DrosMelano7227.FASTAC.txt&AAM68752&4+9^33+11^98+20*) | (AAM68752) CG12324-PA |
| AAN12148 |  | [13.3](http://molbio.princeton.edu/exchange/Attach/orb-x.html#AAN12148) |  | [13.3](http://gemini.scripps.edu/cgi-bin/SeqCov?/wfs/bfd/3/ian/fly/DrosMelano7227.FASTAC.txt&AAN12148&456+29^747+16^771+16^1008+30^1161+31^1225+30^1282+29^1335+29^1792+16^1839+19^1953+12^1966+15^2001+26^2203+14^2384+35&1238+17^1966+15*) | (AAN12148) CG9936-PC |
| AAS64599  Q9V4F7  AAS64601  AAS64600 |  | [0.5](http://molbio.princeton.edu/exchange/Attach/orb-x.html#AAS64599) |  | [0.5](http://gemini.scripps.edu/cgi-bin/SeqCov?/wfs/bfd/3/ian/fly/DrosMelano7227.FASTAC.txt&AAS64599&4515+23^7696+25*)  [0.5](http://gemini.scripps.edu/cgi-bin/SeqCov?/wfs/bfd/3/ian/fly/DrosMelano7227.FASTAC.txt&Q9V4F7&4528+23^7709+25*)  [0.6](http://gemini.scripps.edu/cgi-bin/SeqCov?/wfs/bfd/3/ian/fly/DrosMelano7227.FASTAC.txt&AAS64601&4232+23^7413+25*)  [0.6](http://gemini.scripps.edu/cgi-bin/SeqCov?/wfs/bfd/3/ian/fly/DrosMelano7227.FASTAC.txt&AAS64600&4233+23^7414+25*) | (AAS64599) CG32019-PD  (Q9V4F7) CG32019-PA  (AAS64601) CG32019-PE  (AAS64600) CG32019-PC |
| AAS64933  Q9W093 |  | [5.0](http://molbio.princeton.edu/exchange/Attach/orb-x.html#AAS64933) |  | [5.0](http://gemini.scripps.edu/cgi-bin/SeqCov?/wfs/bfd/3/ian/fly/DrosMelano7227.FASTAC.txt&AAS64933&83+35^803+21*)  [4.3](http://gemini.scripps.edu/cgi-bin/SeqCov?/wfs/bfd/3/ian/fly/DrosMelano7227.FASTAC.txt&Q9W093&83+35^997+21*) | (AAS64933) CG13921-PB  (Q9W093) CG13921-PA |
| AAS64957  Q9VZQ3  AAS64958 |  | [0.8](http://molbio.princeton.edu/exchange/Attach/orb-x.html#AAS64957) |  | [0.8](http://gemini.scripps.edu/cgi-bin/SeqCov?/wfs/bfd/3/ian/fly/DrosMelano7227.FASTAC.txt&AAS64957&1075+19^1666+16*)  [0.9](http://gemini.scripps.edu/cgi-bin/SeqCov?/wfs/bfd/3/ian/fly/DrosMelano7227.FASTAC.txt&Q9VZQ3&1075+19^1666+16*)  [0.8](http://gemini.scripps.edu/cgi-bin/SeqCov?/wfs/bfd/3/ian/fly/DrosMelano7227.FASTAC.txt&AAS64958&1075+19^1666+16*) | (AAS64957) CG12008-PC  (Q9VZQ3) KST protein (Beta-H spectrin)  (AAS64958) CG12008-PB |
| AAS64963  ENC_DROME |  | [2.2](http://molbio.princeton.edu/exchange/Attach/orb-x.html#AAS64963) |  | [2.2](http://gemini.scripps.edu/cgi-bin/SeqCov?/wfs/bfd/3/ian/fly/DrosMelano7227.FASTAC.txt&AAS64963&644+23^1765+17*)  [2.2](http://gemini.scripps.edu/cgi-bin/SeqCov?/wfs/bfd/3/ian/fly/DrosMelano7227.FASTAC.txt&ENC_DROME&639+23^1760+17*) | (AAS64963) CG10847-PA  (Q8MSX1) Encore protein |
| AAS65129  OSKA_DROME |  | [25.4](http://molbio.princeton.edu/exchange/Attach/orb-x.html#AAS65129) |  | [25.4](http://gemini.scripps.edu/cgi-bin/SeqCov?/wfs/bfd/3/ian/fly/DrosMelano7227.FASTAC.txt&AAS65129&24+17^87+12^101+16^179+44^353+12^375+18*)  [19.6](http://gemini.scripps.edu/cgi-bin/SeqCov?/wfs/bfd/3/ian/fly/DrosMelano7227.FASTAC.txt&OSKA_DROME&162+17^225+12^239+16^317+44^491+12^513+18*) | (AAS65129) CG10901-PC  (P25158) Maternal effect protein oskar |
| AAS65166  OSA_DROME |  | [16.8](http://molbio.princeton.edu/exchange/Attach/orb-x.html#AAS65166) |  | [16.8](http://gemini.scripps.edu/cgi-bin/SeqCov?/wfs/bfd/3/ian/fly/DrosMelano7227.FASTAC.txt&AAS65166&706+61^920+60^1082+54^1240+65^1345+29^1637+10^1815+13^1869+10^2035+10^2069+10^2089+17^2142+34^2229+14^2251+42&748+19^961+19^1098+12^1268+37^2069+10&748+19*)  [15.8](http://gemini.scripps.edu/cgi-bin/SeqCov?/wfs/bfd/3/ian/fly/DrosMelano7227.FASTAC.txt&OSA_DROME&706+61^920+60^1082+54^1400+65^1505+29^1797+10^1975+13^2029+10^2195+10^2229+10^2249+17^2302+34^2389+14^2411+42&748+19^961+19^1098+12^1428+37^2229+10&748+19*) | (AAS65166) CG7467-PC  (Q8IN94) Trithorax group protein OSA (Eyelid protein) |
| AAS65273  DC13_DROME  DC12_DROME  DC11_DROME |  | [1.4](http://molbio.princeton.edu/exchange/Attach/orb-x.html#AAS65273) |  | [1.4](http://gemini.scripps.edu/cgi-bin/SeqCov?/wfs/bfd/3/ian/fly/DrosMelano7227.FASTAC.txt&AAS65273&288+14^320+11*)  [1.6](http://gemini.scripps.edu/cgi-bin/SeqCov?/wfs/bfd/3/ian/fly/DrosMelano7227.FASTAC.txt&DC13_DROME&288+14^320+11*)  [2.6](http://gemini.scripps.edu/cgi-bin/SeqCov?/wfs/bfd/3/ian/fly/DrosMelano7227.FASTAC.txt&DC12_DROME&288+14^320+11*)  [2.1](http://gemini.scripps.edu/cgi-bin/SeqCov?/wfs/bfd/3/ian/fly/DrosMelano7227.FASTAC.txt&DC11_DROME&288+14^320+11*) | (AAS65273) CG2175-PD  (P18171) Defective chorion-1 protein, FC177 isoform precursor  (P18170) Defective chorion-1 protein, F106 isoform precursor  (P18169) Defective chorion-1 protein, FC125 isoform precursor |
| AAS65312  Q9VYZ5  Q9VYZ4  DLG1_DROME |  | [5.1](http://molbio.princeton.edu/exchange/Attach/orb-x.html#AAS65312) |  | [5.1](http://gemini.scripps.edu/cgi-bin/SeqCov?/wfs/bfd/3/ian/fly/DrosMelano7227.FASTAC.txt&AAS65312&69+23^315+27*)  [5.2](http://gemini.scripps.edu/cgi-bin/SeqCov?/wfs/bfd/3/ian/fly/DrosMelano7227.FASTAC.txt&Q9VYZ5&77+23^323+27*)  [5.2](http://gemini.scripps.edu/cgi-bin/SeqCov?/wfs/bfd/3/ian/fly/DrosMelano7227.FASTAC.txt&Q9VYZ4&69+23^315+27*)  [5.2](http://gemini.scripps.edu/cgi-bin/SeqCov?/wfs/bfd/3/ian/fly/DrosMelano7227.FASTAC.txt&DLG1_DROME&69+23^315+27*) | (AAS65312) CG1725-PG  (Q9VYZ5) CG1725 protein  (Q9VYZ4) CG1725 protein  (P31007) Discs large-1 tumor suppressor protein |
| ACDM_DROME |  | [9.5](http://molbio.princeton.edu/exchange/Attach/orb-x.html#ACDM_DROME) |  | [9.5](http://gemini.scripps.edu/cgi-bin/SeqCov?/wfs/bfd/3/ian/fly/DrosMelano7227.FASTAC.txt&ACDM_DROME&145+25^276+15&276+15*) | (Q9VSA3) Probable acyl-CoA dehydrogenase, medium-chain specific, mitochondrial precursor (EC 1.3.99.3) (MCAD) |
| BRM_DROME |  | [13.6](http://molbio.princeton.edu/exchange/Attach/orb-x.html#BRM_DROME) |  | [13.6](http://gemini.scripps.edu/cgi-bin/SeqCov?/wfs/bfd/3/ian/fly/DrosMelano7227.FASTAC.txt&BRM_DROME&335+44^513+13^591+20^821+34^912+14^954+19^1064+32^1295+9^1323+13^1591+25&513+13^826+18^1064+14^1591+25*) | (P25439) Homeotic gene regulator (Brahma protein) |
| CH36_DROME |  | [15.7](http://molbio.princeton.edu/exchange/Attach/orb-x.html#CH36_DROME) |  | [15.7](http://gemini.scripps.edu/cgi-bin/SeqCov?/wfs/bfd/3/ian/fly/DrosMelano7227.FASTAC.txt&CH36_DROME&126+13^189+14^222+18*) | (P07182) Chorion protein S36 |
| CYPH_DROME |  | [18.1](http://molbio.princeton.edu/exchange/Attach/orb-x.html#CYPH_DROME) |  | [18.1](http://gemini.scripps.edu/cgi-bin/SeqCov?/wfs/bfd/3/ian/fly/DrosMelano7227.FASTAC.txt&CYPH_DROME&69+14^155+27&155+27*) | (P25007) Peptidyl-prolyl cis-trans isomerase (EC 5.2.1.8) (PPIase) (Rotamase) (Cyclophilin) (Cyclosporin A-binding protein) |
| DNJ1_DROME |  | [4.8](http://molbio.princeton.edu/exchange/Attach/orb-x.html#DNJ1_DROME) |  | [4.8](http://gemini.scripps.edu/cgi-bin/SeqCov?/wfs/bfd/3/ian/fly/DrosMelano7227.FASTAC.txt&DNJ1_DROME&274+16&274+16*) | (Q24133) DnaJ protein homolog 1 (DROJ1) |
| EF11_DROME |  | [25.1](http://molbio.princeton.edu/exchange/Attach/orb-x.html#EF11_DROME) |  | [25.1](http://gemini.scripps.edu/cgi-bin/SeqCov?/wfs/bfd/3/ian/fly/DrosMelano7227.FASTAC.txt&EF11_DROME&85+12^135+12^256+35^323+8^336+36^396+13&336+32*) | (P08736) Elongation factor 1-alpha (EF-1-alpha) (50 kDa female-specific protein) |
| EF12_DROME |  | [17.1](http://molbio.princeton.edu/exchange/Attach/orb-x.html#EF12_DROME) |  | [17.1](http://gemini.scripps.edu/cgi-bin/SeqCov?/wfs/bfd/3/ian/fly/DrosMelano7227.FASTAC.txt&EF12_DROME&85+12^135+12^256+11^323+8^336+36&336+32*) | (P05303) Elongation factor 1-alpha (EF-1-alpha) |
| GBLP_DROME |  | [24.2](http://molbio.princeton.edu/exchange/Attach/orb-x.html#GBLP_DROME) |  | [24.2](http://gemini.scripps.edu/cgi-bin/SeqCov?/wfs/bfd/3/ian/fly/DrosMelano7227.FASTAC.txt&GBLP_DROME&9+18^157+17^187+27^267+15&161+13^187+27*) | (O18640) Guanine nucleotide-binding protein beta subunit-like protein (Receptor of activated protein kinase C homolog) |
| GYS_DROME |  | [2.1](http://molbio.princeton.edu/exchange/Attach/orb-x.html#GYS_DROME) |  | [2.1](http://gemini.scripps.edu/cgi-bin/SeqCov?/wfs/bfd/3/ian/fly/DrosMelano7227.FASTAC.txt&GYS_DROME&318+15&318+15*) | (Q9VFC8) Putative glycogen [starch] synthase (EC 2.4.1.11) |
| HS26_DROME |  | [19.7](http://molbio.princeton.edu/exchange/Attach/orb-x.html#HS26_DROME) |  | [19.7](http://gemini.scripps.edu/cgi-bin/SeqCov?/wfs/bfd/3/ian/fly/DrosMelano7227.FASTAC.txt&HS26_DROME&18+13^74+13^175+15&18+13*) | (P02517) Heat shock protein 26 |
| HS27_DROME |  | [16.4](http://molbio.princeton.edu/exchange/Attach/orb-x.html#HS27_DROME) |  | [16.4](http://gemini.scripps.edu/cgi-bin/SeqCov?/wfs/bfd/3/ian/fly/DrosMelano7227.FASTAC.txt&HS27_DROME&142+20^176+15*) | (P02518) Heat shock protein 27 |
| HS68_DROME |  | [5.7](http://molbio.princeton.edu/exchange/Attach/orb-x.html#HS68_DROME) |  | [5.7](http://gemini.scripps.edu/cgi-bin/SeqCov?/wfs/bfd/3/ian/fly/DrosMelano7227.FASTAC.txt&HS68_DROME&113+11^144+9^169+16&173+12&177+8*) | (O97125) Heat shock protein 68 |
| HS70_DROME  HS7B_DROME  HS74_DROME  HS71_DROME |  | [5.6](http://molbio.princeton.edu/exchange/Attach/orb-x.html#HS70_DROME) |  | [5.6](http://gemini.scripps.edu/cgi-bin/SeqCov?/wfs/bfd/3/ian/fly/DrosMelano7227.FASTAC.txt&HS70_DROME&113+11^144+9^169+16&173+12&177+8*)  [5.7](http://gemini.scripps.edu/cgi-bin/SeqCov?/wfs/bfd/3/ian/fly/DrosMelano7227.FASTAC.txt&HS7B_DROME&116+11^147+9^172+16&176+12&180+8*)  [5.6](http://gemini.scripps.edu/cgi-bin/SeqCov?/wfs/bfd/3/ian/fly/DrosMelano7227.FASTAC.txt&HS74_DROME&113+11^144+9^169+16&173+12&177+8*)  [5.6](http://gemini.scripps.edu/cgi-bin/SeqCov?/wfs/bfd/3/ian/fly/DrosMelano7227.FASTAC.txt&HS71_DROME&113+11^144+9^169+16&173+12&177+8*) | (P82910) Major heat shock 70 kDa protein Aa (Heat shock protein 70Aa) (HSP70-87A7)  (P11146) Heat shock 70 kDa protein cognate 2 (Heat shock 70 kDa protein 87D)  (Q9VG58) Major heat shock 70 kDa protein Bbb (Heat shock protein 70Bbb) (HSP70-87C1)  (P02825) Major heat shock 70 kDa protein Ab (Heat shock protein 70Ab) (HSP70-87A7) |
| IF32_DROME |  | [4.9](http://molbio.princeton.edu/exchange/Attach/orb-x.html#IF32_DROME) |  | [4.9](http://gemini.scripps.edu/cgi-bin/SeqCov?/wfs/bfd/3/ian/fly/DrosMelano7227.FASTAC.txt&IF32_DROME&285+16&285+16*) | (O02195) Eukaryotic translation initiation factor 3 subunit 2 (eIF-3 beta) (eIF3i) (TRIP-1 homolog) |
| IF4A_DROME |  | [8.9](http://molbio.princeton.edu/exchange/Attach/orb-x.html#IF4A_DROME) |  | [8.9](http://gemini.scripps.edu/cgi-bin/SeqCov?/wfs/bfd/3/ian/fly/DrosMelano7227.FASTAC.txt&IF4A_DROME&144+15^383+21*) | (Q02748) Eukaryotic initiation factor 4A (eIF4A) (eIF-4A) |
| IF4E_DROME |  | [27.0](http://molbio.princeton.edu/exchange/Attach/orb-x.html#IF4E_DROME) |  | [27.0](http://gemini.scripps.edu/cgi-bin/SeqCov?/wfs/bfd/3/ian/fly/DrosMelano7227.FASTAC.txt&IF4E_DROME&20+24^49+26^205+20&20+24*) | (P48598) Eukaryotic translation initiation factor 4E (eIF4E) (eIF-4E) (mRNA cap-binding protein) (eIF-4F 25 kDa subunit) |
| IP3R_DROME |  | [2.3](http://molbio.princeton.edu/exchange/Attach/orb-x.html#IP3R_DROME) |  | [2.3](http://gemini.scripps.edu/cgi-bin/SeqCov?/wfs/bfd/3/ian/fly/DrosMelano7227.FASTAC.txt&IP3R_DROME&91+31^2366+34*) | (P29993) Inositol 1,4,5-trisphosphate receptor (InsP3 receptor) (InsP3R) |
| KC2A_DROME |  | [17.9](http://molbio.princeton.edu/exchange/Attach/orb-x.html#KC2A_DROME) |  | [17.9](http://gemini.scripps.edu/cgi-bin/SeqCov?/wfs/bfd/3/ian/fly/DrosMelano7227.FASTAC.txt&KC2A_DROME&105+15^140+13^170+19^245+13&170+19*) | (P08181) Casein kinase II, alpha chain (EC 2.7.1.37) (CK II alpha subunit) |
| NEP1_DROME |  | [16.3](http://molbio.princeton.edu/exchange/Attach/orb-x.html#NEP1_DROME) |  | [16.3](http://gemini.scripps.edu/cgi-bin/SeqCov?/wfs/bfd/3/ian/fly/DrosMelano7227.FASTAC.txt&NEP1_DROME&57+18^133+11^161+12*) | (Q9W4J5) Probable ribosome biogenesis protein NEP1 |
| NONA_DROME  Q9VXH1  Q8IR16 |  | [17.9](http://molbio.princeton.edu/exchange/Attach/orb-x.html#NONA_DROME) |  | [17.9](http://gemini.scripps.edu/cgi-bin/SeqCov?/wfs/bfd/3/ian/fly/DrosMelano7227.FASTAC.txt&NONA_DROME&53+33^236+31^304+16^324+14^451+17^486+14&240+27&240+27*)  [17.9](http://gemini.scripps.edu/cgi-bin/SeqCov?/wfs/bfd/3/ian/fly/DrosMelano7227.FASTAC.txt&Q9VXH1&53+33^236+31^304+16^324+14^451+17^486+14&240+27&240+27*)  [16.8](http://gemini.scripps.edu/cgi-bin/SeqCov?/wfs/bfd/3/ian/fly/DrosMelano7227.FASTAC.txt&Q8IR16&53+33^236+31^304+16^324+14^451+17^486+14&240+27&240+27*) | (Q04047) No-on-transient A protein  (Q9VXH1) CG4211 protein (RE58280p)  (Q8IR16) CG4211-PB |
| O62526 |  | [3.9](http://molbio.princeton.edu/exchange/Attach/orb-x.html#O62526) |  | [3.9](http://gemini.scripps.edu/cgi-bin/SeqCov?/wfs/bfd/3/ian/fly/DrosMelano7227.FASTAC.txt&O62526&91+12&93+10&93+10*) | (O62526) ANT2 protein |
| OTU_DROME |  | [5.7](http://molbio.princeton.edu/exchange/Attach/orb-x.html#OTU_DROME) |  | [5.7](http://gemini.scripps.edu/cgi-bin/SeqCov?/wfs/bfd/3/ian/fly/DrosMelano7227.FASTAC.txt&OTU_DROME&381+22^642+27*) | (P10383) Ovarian tumor locus protein |
| PIT_DROME |  | [5.3](http://molbio.princeton.edu/exchange/Attach/orb-x.html#PIT_DROME) |  | [5.3](http://gemini.scripps.edu/cgi-bin/SeqCov?/wfs/bfd/3/ian/fly/DrosMelano7227.FASTAC.txt&PIT_DROME&164+17^448+19*) | (Q9VD51) Probable ATP-dependent helicase pitchoune |
| Q24090 |  | [46.8](http://molbio.princeton.edu/exchange/Attach/orb-x.html#Q24090) |  | [46.8](http://gemini.scripps.edu/cgi-bin/SeqCov?/wfs/bfd/3/ian/fly/DrosMelano7227.FASTAC.txt&Q24090&59+33^112+26^147+21^193+54^310+39&59+19*) | (Q24090) SNR1 protein (GH08712p) |
| Q26459  Q9VRM5  Q8IQ63 |  | [17.3](http://molbio.princeton.edu/exchange/Attach/orb-x.html#Q26459) |  | [17.3](http://gemini.scripps.edu/cgi-bin/SeqCov?/wfs/bfd/3/ian/fly/DrosMelano7227.FASTAC.txt&Q26459&72+27^103+25^161+16^202+15^277+13^354+9*)  [17.0](http://gemini.scripps.edu/cgi-bin/SeqCov?/wfs/bfd/3/ian/fly/DrosMelano7227.FASTAC.txt&Q9VRM5&81+27^112+25^170+16^211+15^286+13^363+9*)  [16.8](http://gemini.scripps.edu/cgi-bin/SeqCov?/wfs/bfd/3/ian/fly/DrosMelano7227.FASTAC.txt&Q8IQ63&89+27^120+25^178+16^219+15^294+13^371+9*) | (Q26459) MSR-110 protein (LD44960p) (CG10596-PB)  (Q9VRM5) MSR-110 protein  (Q8IQ63) CG10596-PC |
| Q8I0G5  Q9V9W9 |  | [3.2](http://molbio.princeton.edu/exchange/Attach/orb-x.html#Q8I0G5) |  | [3.2](http://gemini.scripps.edu/cgi-bin/SeqCov?/wfs/bfd/3/ian/fly/DrosMelano7227.FASTAC.txt&Q8I0G5&625+15^659+13*)  [3.2](http://gemini.scripps.edu/cgi-bin/SeqCov?/wfs/bfd/3/ian/fly/DrosMelano7227.FASTAC.txt&Q9V9W9&621+15^655+13*) | (Q8I0G5) CG1528-PB (RE37840p)  (Q9V9W9) GAMMACOP protein |
| Q8IMZ2 |  | [30.8](http://molbio.princeton.edu/exchange/Attach/orb-x.html#Q8IMZ2) |  | [30.8](http://gemini.scripps.edu/cgi-bin/SeqCov?/wfs/bfd/3/ian/fly/DrosMelano7227.FASTAC.txt&Q8IMZ2&193+19^222+26^276+17^307+16^343+26^441+13^464+22^512+33&228+14^307+16&312+11*) | (Q8IMZ2) CG10868-PC |
| Q8IMZ3 |  | [23.2](http://molbio.princeton.edu/exchange/Attach/orb-x.html#Q8IMZ3) |  | [23.2](http://gemini.scripps.edu/cgi-bin/SeqCov?/wfs/bfd/3/ian/fly/DrosMelano7227.FASTAC.txt&Q8IMZ3&232+18^254+22^547+19^576+26^630+17^661+16^697+26^795+13^818+22^866+33&582+14^661+16&666+11*) | (Q8IMZ3) CG10868-PB |
| Q8INH9  Q9VG05 |  | [2.4](http://molbio.princeton.edu/exchange/Attach/orb-x.html#Q8INH9) |  | [2.4](http://gemini.scripps.edu/cgi-bin/SeqCov?/wfs/bfd/3/ian/fly/DrosMelano7227.FASTAC.txt&Q8INH9&610+20^1411+19^2008+18*)  [2.3](http://gemini.scripps.edu/cgi-bin/SeqCov?/wfs/bfd/3/ian/fly/DrosMelano7227.FASTAC.txt&Q9VG05&610+20^1411+19^2008+18*) | (Q8INH9) CG7518-PB  (Q9VG05) CG7518 protein |
| Q8INW9 |  | [28.1](http://molbio.princeton.edu/exchange/Attach/orb-x.html#Q8INW9) |  | [28.1](http://gemini.scripps.edu/cgi-bin/SeqCov?/wfs/bfd/3/ian/fly/DrosMelano7227.FASTAC.txt&Q8INW9&22+13^159+34^253+41^335+30^510+17^542+24&542+16&543+15&543+15*) | (Q8INW9) CG15825-PA |
| Q8IPB9  Q9VKX1 |  | [5.2](http://molbio.princeton.edu/exchange/Attach/orb-x.html#Q8IPB9) |  | [5.2](http://gemini.scripps.edu/cgi-bin/SeqCov?/wfs/bfd/3/ian/fly/DrosMelano7227.FASTAC.txt&Q8IPB9&621+27^688+27*)  [5.1](http://gemini.scripps.edu/cgi-bin/SeqCov?/wfs/bfd/3/ian/fly/DrosMelano7227.FASTAC.txt&Q9VKX1&625+27^692+27*) | (Q8IPB9) CG31716-PG  (Q9VKX1) CG31716 protein (RE04975p) |
| Q8IQJ0 |  | [14.4](http://molbio.princeton.edu/exchange/Attach/orb-x.html#Q8IQJ0) |  | [14.4](http://gemini.scripps.edu/cgi-bin/SeqCov?/wfs/bfd/3/ian/fly/DrosMelano7227.FASTAC.txt&Q8IQJ0&32+16&34+14&35+13*) | (Q8IQJ0) CG11271-PC |
| Q8IQV9  Q9VWB8 |  | [0.9](http://molbio.princeton.edu/exchange/Attach/orb-x.html#Q8IQV9) |  | [0.9](http://gemini.scripps.edu/cgi-bin/SeqCov?/wfs/bfd/3/ian/fly/DrosMelano7227.FASTAC.txt&Q8IQV9&1249+18&1249+18*)  [0.9](http://gemini.scripps.edu/cgi-bin/SeqCov?/wfs/bfd/3/ian/fly/DrosMelano7227.FASTAC.txt&Q9VWB8&1226+18&1226+18*) | (Q8IQV9) CG11943-PB  (Q9VWB8) CG11943 protein |
| Q8IRI9 |  | [17.4](http://molbio.princeton.edu/exchange/Attach/orb-x.html#Q8IRI9) |  | [17.4](http://gemini.scripps.edu/cgi-bin/SeqCov?/wfs/bfd/3/ian/fly/DrosMelano7227.FASTAC.txt&Q8IRI9&116+25^434+32^505+11^574+11^662+32^972+25^1004+12^1029+43^1203+40&128+13^446+20&128+13*) | (Q8IRI9) CG12031-PB |
| Q8IRM8  Q9W392 |  | [6.4](http://molbio.princeton.edu/exchange/Attach/orb-x.html#Q8IRM8) |  | [6.4](http://gemini.scripps.edu/cgi-bin/SeqCov?/wfs/bfd/3/ian/fly/DrosMelano7227.FASTAC.txt&Q8IRM8&119+34*)  [6.4](http://gemini.scripps.edu/cgi-bin/SeqCov?/wfs/bfd/3/ian/fly/DrosMelano7227.FASTAC.txt&Q9W392&121+34*) | (Q8IRM8) CG7033-PB  (Q9W392) CG7033 protein (SD02216p) |
| Q8MKM0  Q9V4M6  Q8MKM1 |  | [4.4](http://molbio.princeton.edu/exchange/Attach/orb-x.html#Q8MKM0) |  | [4.4](http://gemini.scripps.edu/cgi-bin/SeqCov?/wfs/bfd/3/ian/fly/DrosMelano7227.FASTAC.txt&Q8MKM0&65+27^330+15^471+11*)  [3.0](http://gemini.scripps.edu/cgi-bin/SeqCov?/wfs/bfd/3/ian/fly/DrosMelano7227.FASTAC.txt&Q9V4M6&65+27^330+15^471+11*)  [2.9](http://gemini.scripps.edu/cgi-bin/SeqCov?/wfs/bfd/3/ian/fly/DrosMelano7227.FASTAC.txt&Q8MKM1&65+27^330+15^471+11*) | (Q8MKM0) CG2146-PB  (Q9V4M6) Didum protein  (Q8MKM1) CG2146-PC |
| Q8MKX9  Q9V5L1 |  | [12.4](http://molbio.princeton.edu/exchange/Attach/orb-x.html#Q8MKX9) |  | [12.4](http://gemini.scripps.edu/cgi-bin/SeqCov?/wfs/bfd/3/ian/fly/DrosMelano7227.FASTAC.txt&Q8MKX9&203+24^262+34^561+16&561+16*)  [7.7](http://gemini.scripps.edu/cgi-bin/SeqCov?/wfs/bfd/3/ian/fly/DrosMelano7227.FASTAC.txt&Q9V5L1&565+24^624+34^923+16&923+16*) | (Q8MKX9) CG11763-PC  (Q9V5L1) CG11763 protein |
| Q8ML06  Q9V679 |  | [18.8](http://molbio.princeton.edu/exchange/Attach/orb-x.html#Q8ML06) |  | [18.8](http://gemini.scripps.edu/cgi-bin/SeqCov?/wfs/bfd/3/ian/fly/DrosMelano7227.FASTAC.txt&Q8ML06&104+29&104+11*)  [18.7](http://gemini.scripps.edu/cgi-bin/SeqCov?/wfs/bfd/3/ian/fly/DrosMelano7227.FASTAC.txt&Q9V679&105+29&105+11*) | (Q8ML06) CG8857-PB  (Q9V679) CG8857 protein |
| Q8ML98  Q8ML99 |  | [14.0](http://molbio.princeton.edu/exchange/Attach/orb-x.html#Q8ML98) |  | [14.0](http://gemini.scripps.edu/cgi-bin/SeqCov?/wfs/bfd/3/ian/fly/DrosMelano7227.FASTAC.txt&Q8ML98&142+21^399+32^575+19^595+13*)  [11.1](http://gemini.scripps.edu/cgi-bin/SeqCov?/wfs/bfd/3/ian/fly/DrosMelano7227.FASTAC.txt&Q8ML99&299+21^556+32^732+19^752+13*) | (Q8ML98) CG8174-PC  (Q8ML99) CG8174-PA |
| Q8MMB3  Q9V8R3 |  | [16.8](http://molbio.princeton.edu/exchange/Attach/orb-x.html#Q8MMB3) |  | [16.8](http://gemini.scripps.edu/cgi-bin/SeqCov?/wfs/bfd/3/ian/fly/DrosMelano7227.FASTAC.txt&Q8MMB3&198+14^215+12^248+12^293+35^655+26^729+40&296+32*)  [20.3](http://gemini.scripps.edu/cgi-bin/SeqCov?/wfs/bfd/3/ian/fly/DrosMelano7227.FASTAC.txt&Q9V8R3&53+14^70+12^103+12^148+35^510+26^584+40&151+32*) | (Q8MMB3) CG15112-PB  (Q9V8R3) CG15112 protein |
| Q95WY3 |  | [5.0](http://molbio.princeton.edu/exchange/Attach/orb-x.html#Q95WY3) |  | [5.0](http://gemini.scripps.edu/cgi-bin/SeqCov?/wfs/bfd/3/ian/fly/DrosMelano7227.FASTAC.txt&Q95WY3&223+12^309+13*) | (Q95WY3) Nucleolar KKE/D repeat protein (CG13849-PA) |
| Q960X9  Q9VW20 |  | [8.7](http://molbio.princeton.edu/exchange/Attach/orb-x.html#Q960X9) |  | [8.7](http://gemini.scripps.edu/cgi-bin/SeqCov?/wfs/bfd/3/ian/fly/DrosMelano7227.FASTAC.txt&Q960X9&173+25^367+12^447+35*)  [7.2](http://gemini.scripps.edu/cgi-bin/SeqCov?/wfs/bfd/3/ian/fly/DrosMelano7227.FASTAC.txt&Q9VW20&347+25^541+12^621+35*) | (Q960X9) LD30525p (EC 3.4.21.-) (Mitochondrial Lon protease homolog) (CG8798-PB)  (Q9VW20) CG8798 protein (EC 3.4.21.-) (Mitochondrial Lon protease homolog) |
| Q9V378 |  | [3.8](http://molbio.princeton.edu/exchange/Attach/orb-x.html#Q9V378) |  | [3.8](http://gemini.scripps.edu/cgi-bin/SeqCov?/wfs/bfd/3/ian/fly/DrosMelano7227.FASTAC.txt&Q9V378&419+11^543+19*) | (Q9V378) BCDNA:GM05306 protein (LD15481p) |
| Q9V3A8 |  | [42.8](http://molbio.princeton.edu/exchange/Attach/orb-x.html#Q9V3A8) |  | [42.8](http://gemini.scripps.edu/cgi-bin/SeqCov?/wfs/bfd/3/ian/fly/DrosMelano7227.FASTAC.txt&Q9V3A8&39+32^107+14^150+33^195+28^295+23^321+14^357+44^413+31&39+32*) | (Q9V3A8) CG6822 protein (LD43551P) |
| Q9V3G1 |  | [3.9](http://molbio.princeton.edu/exchange/Attach/orb-x.html#Q9V3G1) |  | [3.9](http://gemini.scripps.edu/cgi-bin/SeqCov?/wfs/bfd/3/ian/fly/DrosMelano7227.FASTAC.txt&Q9V3G1&55+10&55+10*) | (Q9V3G1) CG1263 protein (RE37829p) |
| Q9V3J4 |  | [7.6](http://molbio.princeton.edu/exchange/Attach/orb-x.html#Q9V3J4) |  | [7.6](http://gemini.scripps.edu/cgi-bin/SeqCov?/wfs/bfd/3/ian/fly/DrosMelano7227.FASTAC.txt&Q9V3J4&146+27&151+22*) | (Q9V3J4) BCDNA:LD03471 protein |
| Q9V3W7 |  | [15.3](http://molbio.princeton.edu/exchange/Attach/orb-x.html#Q9V3W7) |  | [15.3](http://gemini.scripps.edu/cgi-bin/SeqCov?/wfs/bfd/3/ian/fly/DrosMelano7227.FASTAC.txt&Q9V3W7&43+13^117+16^149+10*) | (Q9V3W7) CG6987 protein (SR family splicing factor) (LD40489P) |
| Q9V496 |  | [1.0](http://molbio.princeton.edu/exchange/Attach/orb-x.html#Q9V496) |  | [1.0](http://gemini.scripps.edu/cgi-bin/SeqCov?/wfs/bfd/3/ian/fly/DrosMelano7227.FASTAC.txt&Q9V496&808+20^3219+13*) | (Q9V496) CG11064 protein |
| Q9V4Z5 |  | [5.0](http://molbio.princeton.edu/exchange/Attach/orb-x.html#Q9V4Z5) |  | [5.0](http://gemini.scripps.edu/cgi-bin/SeqCov?/wfs/bfd/3/ian/fly/DrosMelano7227.FASTAC.txt&Q9V4Z5&428+11^462+20*) | (Q9V4Z5) CG30349 protein |
| Q9V814 |  | [27.8](http://molbio.princeton.edu/exchange/Attach/orb-x.html#Q9V814) |  | [27.8](http://gemini.scripps.edu/cgi-bin/SeqCov?/wfs/bfd/3/ian/fly/DrosMelano7227.FASTAC.txt&Q9V814&37+22^73+12^95+12^141+18^170+30^283+24&283+24*) | (Q9V814) CG6546 protein (LD29458p) |
| Q9V8K6 |  | [7.2](http://molbio.princeton.edu/exchange/Attach/orb-x.html#Q9V8K6) |  | [7.2](http://gemini.scripps.edu/cgi-bin/SeqCov?/wfs/bfd/3/ian/fly/DrosMelano7227.FASTAC.txt&Q9V8K6&38+19^300+13^353+14^383+19^569+27*) | (Q9V8K6) CG30122 protein |
| Q9V9G5 |  | [7.9](http://molbio.princeton.edu/exchange/Attach/orb-x.html#Q9V9G5) |  | [7.9](http://gemini.scripps.edu/cgi-bin/SeqCov?/wfs/bfd/3/ian/fly/DrosMelano7227.FASTAC.txt&Q9V9G5&499+35^669+32*) | (Q9V9G5) CG8426 protein (LD13864p) |
| Q9V9W2  Q9V9W3 |  | [6.6](http://molbio.princeton.edu/exchange/Attach/orb-x.html#Q9V9W2) |  | [6.6](http://gemini.scripps.edu/cgi-bin/SeqCov?/wfs/bfd/3/ian/fly/DrosMelano7227.FASTAC.txt&Q9V9W2&225+16&225+16*)  [6.1](http://gemini.scripps.edu/cgi-bin/SeqCov?/wfs/bfd/3/ian/fly/DrosMelano7227.FASTAC.txt&Q9V9W3&244+16&244+16*) | (Q9V9W2) CG11522 protein  (Q9V9W3) CG11522 protein (RE08669p) |
| Q9VC28 |  | [10.6](http://molbio.princeton.edu/exchange/Attach/orb-x.html#Q9VC28) |  | [10.6](http://gemini.scripps.edu/cgi-bin/SeqCov?/wfs/bfd/3/ian/fly/DrosMelano7227.FASTAC.txt&Q9VC28&97+19&97+19*) | (Q9VC28) CG7006 protein (GM12126p) |
| Q9VCA8 |  | [7.3](http://molbio.princeton.edu/exchange/Attach/orb-x.html#Q9VCA8) |  | [7.3](http://gemini.scripps.edu/cgi-bin/SeqCov?/wfs/bfd/3/ian/fly/DrosMelano7227.FASTAC.txt&Q9VCA8&253+21^703+17^808+20^1388+23^1558+13^1822+28^1942+16^2345+16^2410+21^2823+19^2919+25^3294+17^3743+30^3877+27&253+21^1388+23^2345+16*) | (Q9VCA8) CG33106-PA (CG33106-PB) |
| Q9VCN7 |  | [26.7](http://molbio.princeton.edu/exchange/Attach/orb-x.html#Q9VCN7) |  | [26.7](http://gemini.scripps.edu/cgi-bin/SeqCov?/wfs/bfd/3/ian/fly/DrosMelano7227.FASTAC.txt&Q9VCN7&2+32^234+18^256+22^549+19^578+26^632+17^663+16^699+26^797+13^820+22^868+33&21+13^584+14^663+16&668+11*) | (Q9VCN7) ORB protein |
| Q9VDR1 |  | [12.9](http://molbio.princeton.edu/exchange/Attach/orb-x.html#Q9VDR1) |  | [12.9](http://gemini.scripps.edu/cgi-bin/SeqCov?/wfs/bfd/3/ian/fly/DrosMelano7227.FASTAC.txt&Q9VDR1&67+21^137+20^173+10^543+14^579+12^594+17^645+17&579+12*) | (Q9VDR1) CG12254 protein (LD07688p) |
| Q9VEC1 |  | [14.2](http://molbio.princeton.edu/exchange/Attach/orb-x.html#Q9VEC1) |  | [14.2](http://gemini.scripps.edu/cgi-bin/SeqCov?/wfs/bfd/3/ian/fly/DrosMelano7227.FASTAC.txt&Q9VEC1&30+11^61+26^311+19^416+18^534+17*) | (Q9VEC1) CG7957 protein (Mediator transcriptional cofactor TRAP80) (Thyroid hormone receptor-associated protein TRAP80) (SD10038p) |
| Q9VFV9 |  | [7.7](http://molbio.princeton.edu/exchange/Attach/orb-x.html#Q9VFV9) |  | [7.7](http://gemini.scripps.edu/cgi-bin/SeqCov?/wfs/bfd/3/ian/fly/DrosMelano7227.FASTAC.txt&Q9VFV9&47+14^168+17*) | (Q9VFV9) CG8863 protein (GM13664p) |
| Q9VH01 |  | [11.0](http://molbio.princeton.edu/exchange/Attach/orb-x.html#Q9VH01) |  | [11.0](http://gemini.scripps.edu/cgi-bin/SeqCov?/wfs/bfd/3/ian/fly/DrosMelano7227.FASTAC.txt&Q9VH01&89+12^207+14^450+16^1085+19^1534+22^1940+19^2169+10^2401+53^2665+22^2852+26^3026+13^3216+22^3258+21^3284+37^3332+12^3401+36^3654+19^3701+16^3738+26^3803+30^4074+41^4185+17^4285+21^4652+16&2440+14^3701+16*) | (Q9VH01) CG6303 protein (EC 6.3.2.19) (Ubiquitin-conjugating enzyme E2) (Ubiquitin-protein ligase) (Ubiquitin carrier protein) |
| Q9VHI5 |  | [10.6](http://molbio.princeton.edu/exchange/Attach/orb-x.html#Q9VHI5) |  | [10.6](http://gemini.scripps.edu/cgi-bin/SeqCov?/wfs/bfd/3/ian/fly/DrosMelano7227.FASTAC.txt&Q9VHI5&550+36^591+32*) | (Q9VHI5) CG11984-PA |
| Q9VI82 |  | [2.8](http://molbio.princeton.edu/exchange/Attach/orb-x.html#Q9VI82) |  | [2.8](http://gemini.scripps.edu/cgi-bin/SeqCov?/wfs/bfd/3/ian/fly/DrosMelano7227.FASTAC.txt&Q9VI82&726+23&726+23*) | (Q9VI82) CG1234 protein (LD35257P) |
| Q9VIX5  Q9VIX6 |  | [16.0](http://molbio.princeton.edu/exchange/Attach/orb-x.html#Q9VIX5) |  | [16.0](http://gemini.scripps.edu/cgi-bin/SeqCov?/wfs/bfd/3/ian/fly/DrosMelano7227.FASTAC.txt&Q9VIX5&184+42*)  [14.6](http://gemini.scripps.edu/cgi-bin/SeqCov?/wfs/bfd/3/ian/fly/DrosMelano7227.FASTAC.txt&Q9VIX6&209+42*) | (Q9VIX5) CG17549 protein  (Q9VIX6) GH19142p (CG17549-PA) |
| Q9VIX7 |  | [27.6](http://molbio.princeton.edu/exchange/Attach/orb-x.html#Q9VIX7) |  | [27.6](http://gemini.scripps.edu/cgi-bin/SeqCov?/wfs/bfd/3/ian/fly/DrosMelano7227.FASTAC.txt&Q9VIX7&34+13^171+34^265+41^347+30^522+17^554+24&554+16&555+15&555+15*) | (Q9VIX7) CG15825 protein (HL01053p) |
| Q9VJY6 |  | [18.7](http://molbio.princeton.edu/exchange/Attach/orb-x.html#Q9VJY6) |  | [18.7](http://gemini.scripps.edu/cgi-bin/SeqCov?/wfs/bfd/3/ian/fly/DrosMelano7227.FASTAC.txt&Q9VJY6&28+7^48+9^81+13*) | (Q9VJY6) CG9282 protein (RE30690p) |
| Q9VK58 |  | [3.4](http://molbio.princeton.edu/exchange/Attach/orb-x.html#Q9VK58) |  | [3.4](http://gemini.scripps.edu/cgi-bin/SeqCov?/wfs/bfd/3/ian/fly/DrosMelano7227.FASTAC.txt&Q9VK58&660+28^1060+9*) | (Q9VK58) CG5792 protein |
| Q9VK59 |  | [6.5](http://molbio.princeton.edu/exchange/Attach/orb-x.html#Q9VK59) |  | [6.5](http://gemini.scripps.edu/cgi-bin/SeqCov?/wfs/bfd/3/ian/fly/DrosMelano7227.FASTAC.txt&Q9VK59&356+35^426+27*) | (Q9VK59) CG5787 protein (LD23647p) |
| Q9VKG8 |  | [6.1](http://molbio.princeton.edu/exchange/Attach/orb-x.html#Q9VKG8) |  | [6.1](http://gemini.scripps.edu/cgi-bin/SeqCov?/wfs/bfd/3/ian/fly/DrosMelano7227.FASTAC.txt&Q9VKG8&809+31^971+17^1024+42^1375+27*) | (Q9VKG8) CG6509 protein (LD32687p) |
| Q9VNG0 |  | [10.9](http://molbio.princeton.edu/exchange/Attach/orb-x.html#Q9VNG0) |  | [10.9](http://gemini.scripps.edu/cgi-bin/SeqCov?/wfs/bfd/3/ian/fly/DrosMelano7227.FASTAC.txt&Q9VNG0&106+18^151+14*) | (Q9VNG0) CG1245 protein (RE51713p) |
| Q9VP05 |  | [10.0](http://molbio.princeton.edu/exchange/Attach/orb-x.html#Q9VP05) |  | [10.0](http://gemini.scripps.edu/cgi-bin/SeqCov?/wfs/bfd/3/ian/fly/DrosMelano7227.FASTAC.txt&Q9VP05&322+13^393+19^527+59^616+26^739+13^809+18&739+13*) | (Q9VP05) CG7162 protein (Thyroid hormone receptor-associated protein TRAP220) |
| Q9VS38 |  | [13.6](http://molbio.princeton.edu/exchange/Attach/orb-x.html#Q9VS38) |  | [13.6](http://gemini.scripps.edu/cgi-bin/SeqCov?/wfs/bfd/3/ian/fly/DrosMelano7227.FASTAC.txt&Q9VS38&33+16^131+19*) | (Q9VS38) CG8609 protein (LD46084P) |
| Q9VSF2 |  | [5.7](http://molbio.princeton.edu/exchange/Attach/orb-x.html#Q9VSF2) |  | [5.7](http://gemini.scripps.edu/cgi-bin/SeqCov?/wfs/bfd/3/ian/fly/DrosMelano7227.FASTAC.txt&Q9VSF2&328+35^418+11^706+11*) | (Q9VSF2) CG7999 protein (Thyroid hormone receptor-associated protein TRAP100) |
| Q9VUE5 |  | [3.7](http://molbio.princeton.edu/exchange/Attach/orb-x.html#Q9VUE5) |  | [3.7](http://gemini.scripps.edu/cgi-bin/SeqCov?/wfs/bfd/3/ian/fly/DrosMelano7227.FASTAC.txt&Q9VUE5&384+16^486+22*) | (Q9VUE5) STWL protein (LD17962p) |
| Q9VUQ5  Q9VUQ6 |  | [18.2](http://molbio.princeton.edu/exchange/Attach/orb-x.html#Q9VUQ5) |  | [18.2](http://gemini.scripps.edu/cgi-bin/SeqCov?/wfs/bfd/3/ian/fly/DrosMelano7227.FASTAC.txt&Q9VUQ5&45+28^388+43^460+14^535+16^585+15^679+10^694+14^751+18^784+12^873+10^884+14^1134+28&410+21^585+15^694+14*)  [18.3](http://gemini.scripps.edu/cgi-bin/SeqCov?/wfs/bfd/3/ian/fly/DrosMelano7227.FASTAC.txt&Q9VUQ6&42+28^385+43^457+14^532+16^582+15^676+10^691+14^748+18^781+12^870+10^881+14^1131+28&407+21^582+15^691+14*) | (Q9VUQ5) CG7439 protein  (Q9VUQ6) CG7439 protein |
| Q9VVI2 |  | [36.0](http://molbio.princeton.edu/exchange/Attach/orb-x.html#Q9VVI2) |  | [36.0](http://gemini.scripps.edu/cgi-bin/SeqCov?/wfs/bfd/3/ian/fly/DrosMelano7227.FASTAC.txt&Q9VVI2&30+11^64+59^235+50^357+18^388+16^455+14^503+32^575+19^620+15^645+11&64+29^388+16^455+14^503+17^582+12&390+14*) | (Q9VVI2) CG6311 protein |
| Q9VVL6 |  | [10.7](http://molbio.princeton.edu/exchange/Attach/orb-x.html#Q9VVL6) |  | [10.7](http://gemini.scripps.edu/cgi-bin/SeqCov?/wfs/bfd/3/ian/fly/DrosMelano7227.FASTAC.txt&Q9VVL6&43+14^173+22*) | (Q9VVL6) CG5546 protein (LD41395p) |
| Q9VW47 |  | [7.4](http://molbio.princeton.edu/exchange/Attach/orb-x.html#Q9VW47) |  | [7.4](http://gemini.scripps.edu/cgi-bin/SeqCov?/wfs/bfd/3/ian/fly/DrosMelano7227.FASTAC.txt&Q9VW47&40+25^408+16^480+25^809+17^1219+11^1437+17^1820+11^1899+20^2415+46*) | (Q9VW47) CG8491 protein (Transcriptional coactivator kohtalo) |
| Q9VXR3 |  | [0.9](http://molbio.princeton.edu/exchange/Attach/orb-x.html#Q9VXR3) |  | [0.9](http://gemini.scripps.edu/cgi-bin/SeqCov?/wfs/bfd/3/ian/fly/DrosMelano7227.FASTAC.txt&Q9VXR3&2115+26^2890+20*) | (Q9VXR3) CG8184 protein |
| Q9VYS4 |  | [6.6](http://molbio.princeton.edu/exchange/Attach/orb-x.html#Q9VYS4) |  | [6.6](http://gemini.scripps.edu/cgi-bin/SeqCov?/wfs/bfd/3/ian/fly/DrosMelano7227.FASTAC.txt&Q9VYS4&271+21^391+21^652+19^849+29&391+21*) | (Q9VYS4) CG15737 protein |
| Q9VZF4 |  | [3.5](http://molbio.princeton.edu/exchange/Attach/orb-x.html#Q9VZF4) |  | [3.5](http://gemini.scripps.edu/cgi-bin/SeqCov?/wfs/bfd/3/ian/fly/DrosMelano7227.FASTAC.txt&Q9VZF4&456+16^800+30*) | (Q9VZF4) LD21322p (LD30271p) (CG15010-PA) (CG15010-PB) |
| Q9VZN0 |  | [4.5](http://molbio.princeton.edu/exchange/Attach/orb-x.html#Q9VZN0) |  | [4.5](http://gemini.scripps.edu/cgi-bin/SeqCov?/wfs/bfd/3/ian/fly/DrosMelano7227.FASTAC.txt&Q9VZN0&232+28^586+17*) | (Q9VZN0) CG32264 protein |
| Q9W0A8 |  | [5.4](http://molbio.princeton.edu/exchange/Attach/orb-x.html#Q9W0A8) |  | [5.4](http://gemini.scripps.edu/cgi-bin/SeqCov?/wfs/bfd/3/ian/fly/DrosMelano7227.FASTAC.txt&Q9W0A8&210+15&210+15&211+14*) | (Q9W0A8) RPL23A protein |
| Q9W0P8 |  | [16.2](http://molbio.princeton.edu/exchange/Attach/orb-x.html#Q9W0P8) |  | [16.2](http://gemini.scripps.edu/cgi-bin/SeqCov?/wfs/bfd/3/ian/fly/DrosMelano7227.FASTAC.txt&Q9W0P8&135+20^338+25^656+32^727+11^796+11^884+32^1194+25^1226+12^1251+43^1425+40&350+13^668+20&350+13*) | (Q9W0P8) CG12031-PA |
| Q9W0S7 |  | [4.0](http://molbio.princeton.edu/exchange/Attach/orb-x.html#Q9W0S7) |  | [4.0](http://gemini.scripps.edu/cgi-bin/SeqCov?/wfs/bfd/3/ian/fly/DrosMelano7227.FASTAC.txt&Q9W0S7&180+19^734+18*) | (Q9W0S7) CG7008 protein (LD20211p) |
| Q9W105 |  | [12.7](http://molbio.princeton.edu/exchange/Attach/orb-x.html#Q9W105) |  | [12.7](http://gemini.scripps.edu/cgi-bin/SeqCov?/wfs/bfd/3/ian/fly/DrosMelano7227.FASTAC.txt&Q9W105&92+22^131+16^185+11*) | (Q9W105) CG16910 protein |
| Q9W1G7 |  | [7.8](http://molbio.princeton.edu/exchange/Attach/orb-x.html#Q9W1G7) |  | [7.8](http://gemini.scripps.edu/cgi-bin/SeqCov?/wfs/bfd/3/ian/fly/DrosMelano7227.FASTAC.txt&Q9W1G7&145+11^213+18*) | (Q9W1G7) NAP1 protein (LD21576p) |
| Q9W1H5 |  | [37.4](http://molbio.princeton.edu/exchange/Attach/orb-x.html#Q9W1H5) |  | [37.4](http://gemini.scripps.edu/cgi-bin/SeqCov?/wfs/bfd/3/ian/fly/DrosMelano7227.FASTAC.txt&Q9W1H5&23+48^102+24^147+15^177+14^207+11^221+14^247+13&23+23^104+12^221+14*) | (Q9W1H5) CG11183 protein |
| Q9W1H8 |  | [6.8](http://molbio.princeton.edu/exchange/Attach/orb-x.html#Q9W1H8) |  | [6.8](http://gemini.scripps.edu/cgi-bin/SeqCov?/wfs/bfd/3/ian/fly/DrosMelano7227.FASTAC.txt&Q9W1H8&72+9^330+23*) | (Q9W1H8) Thiolase protein (GH13256p) |
| Q9W1X7 |  | [6.9](http://molbio.princeton.edu/exchange/Attach/orb-x.html#Q9W1X7) |  | [6.9](http://gemini.scripps.edu/cgi-bin/SeqCov?/wfs/bfd/3/ian/fly/DrosMelano7227.FASTAC.txt&Q9W1X7&40+17^252+18^279+11^445+15^655+16^1295+9^1323+14&1323+14*) | (Q9W1X7) Trap150-beta protein |
| Q9W255 |  | [11.1](http://molbio.princeton.edu/exchange/Attach/orb-x.html#Q9W255) |  | [11.1](http://gemini.scripps.edu/cgi-bin/SeqCov?/wfs/bfd/3/ian/fly/DrosMelano7227.FASTAC.txt&Q9W255&20+30^96+14&21+29*) | (Q9W255) QRK58E-1 protein (GH05812p) |
| Q9W278 |  | [10.8](http://molbio.princeton.edu/exchange/Attach/orb-x.html#Q9W278) |  | [10.8](http://gemini.scripps.edu/cgi-bin/SeqCov?/wfs/bfd/3/ian/fly/DrosMelano7227.FASTAC.txt&Q9W278&207+27^338+32^511+11^569+18*) | (Q9W278) CG5465 protein |
| Q9W2U7 |  | [6.3](http://molbio.princeton.edu/exchange/Attach/orb-x.html#Q9W2U7) |  | [6.3](http://gemini.scripps.edu/cgi-bin/SeqCov?/wfs/bfd/3/ian/fly/DrosMelano7227.FASTAC.txt&Q9W2U7&189+42^352+33^810+13^1282+15^1452+14^1828+28*) | (Q9W2U7) CG17255 protein |
| Q9W3G1 |  | [4.8](http://molbio.princeton.edu/exchange/Attach/orb-x.html#Q9W3G1) |  | [4.8](http://gemini.scripps.edu/cgi-bin/SeqCov?/wfs/bfd/3/ian/fly/DrosMelano7227.FASTAC.txt&Q9W3G1&127+44*) | (Q9W3G1) CG10555 protein |
| Q9W3L1 |  | [4.4](http://molbio.princeton.edu/exchange/Attach/orb-x.html#Q9W3L1) |  | [4.4](http://gemini.scripps.edu/cgi-bin/SeqCov?/wfs/bfd/3/ian/fly/DrosMelano7227.FASTAC.txt&Q9W3L1&84+16^368+39*) | (Q9W3L1) CG2253 protein |
| Q9W417  UBIQ_DROME  Q9W418 |  | [15.3](http://molbio.princeton.edu/exchange/Attach/orb-x.html#Q9W417) |  | [15.3](http://gemini.scripps.edu/cgi-bin/SeqCov?/wfs/bfd/3/ian/fly/DrosMelano7227.FASTAC.txt&Q9W417&12+16^195+30&201+24&207+18*)  [60.5](http://gemini.scripps.edu/cgi-bin/SeqCov?/wfs/bfd/3/ian/fly/DrosMelano7227.FASTAC.txt&UBIQ_DROME&12+16^43+30&49+24&55+18*)  [8.6](http://gemini.scripps.edu/cgi-bin/SeqCov?/wfs/bfd/3/ian/fly/DrosMelano7227.FASTAC.txt&Q9W418&12+16^43+30&49+24&55+18*) | (Q9W417) CG11700 protein  (Q9VZL4) Ubiquitin  (Q9W418) CG32744 protein |
| Q9W4L2 |  | [6.9](http://molbio.princeton.edu/exchange/Attach/orb-x.html#Q9W4L2) |  | [6.9](http://gemini.scripps.edu/cgi-bin/SeqCov?/wfs/bfd/3/ian/fly/DrosMelano7227.FASTAC.txt&Q9W4L2&216+12^464+15^517+18&517+18*) | (Q9W4L2) CG2982 protein (LD40453p) |
| Q9Y0Z1 |  | [4.0](http://molbio.princeton.edu/exchange/Attach/orb-x.html#Q9Y0Z1) |  | [4.0](http://gemini.scripps.edu/cgi-bin/SeqCov?/wfs/bfd/3/ian/fly/DrosMelano7227.FASTAC.txt&Q9Y0Z1&944+32^1013+35*) | (Q9Y0Z1) BCDNA:LD23876 protein |
| Q9Y108 |  | [4.0](http://molbio.princeton.edu/exchange/Attach/orb-x.html#Q9Y108) |  | [4.0](http://gemini.scripps.edu/cgi-bin/SeqCov?/wfs/bfd/3/ian/fly/DrosMelano7227.FASTAC.txt&Q9Y108&297+18^347+14*) | (Q9Y108) BCDNA:GH11110 protein |
| RB27_DROME |  | [26.8](http://molbio.princeton.edu/exchange/Attach/orb-x.html#RB27_DROME) |  | [26.8](http://gemini.scripps.edu/cgi-bin/SeqCov?/wfs/bfd/3/ian/fly/DrosMelano7227.FASTAC.txt&RB27_DROME&9+17^49+27^98+16^303+20^332+23*) | (P48809) Heterogeneous nuclear ribonucleoprotein 27C (hnRNP 48) (HRP48.1) |
| RF2P_DROME |  | [28.0](http://molbio.princeton.edu/exchange/Attach/orb-x.html#RF2P_DROME) |  | [28.0](http://gemini.scripps.edu/cgi-bin/SeqCov?/wfs/bfd/3/ian/fly/DrosMelano7227.FASTAC.txt&RF2P_DROME&79+19^168+21^196+19^226+51^347+24^462+34&196+19^265+12^486+10*) | (P14199) Ref(2)P protein (Refractory to sigma P) |
| RL14_DROME |  | [14.5](http://molbio.princeton.edu/exchange/Attach/orb-x.html#RL14_DROME) |  | [14.5](http://gemini.scripps.edu/cgi-bin/SeqCov?/wfs/bfd/3/ian/fly/DrosMelano7227.FASTAC.txt&RL14_DROME&24+24*) | (P55841) 60S ribosomal protein L14 |
| RL19_DROME |  | [11.8](http://molbio.princeton.edu/exchange/Attach/orb-x.html#RL19_DROME) |  | [11.8](http://gemini.scripps.edu/cgi-bin/SeqCov?/wfs/bfd/3/ian/fly/DrosMelano7227.FASTAC.txt&RL19_DROME&22+17^191+7*) | (P36241) 60S ribosomal protein L19 |
| RL1X_DROME |  | [26.6](http://molbio.princeton.edu/exchange/Attach/orb-x.html#RL1X_DROME) |  | [26.6](http://gemini.scripps.edu/cgi-bin/SeqCov?/wfs/bfd/3/ian/fly/DrosMelano7227.FASTAC.txt&RL1X_DROME&32+18^99+13^121+16*) | (P41093) 60S ribosomal protein L18a |
| RL23_DROME |  | [32.9](http://molbio.princeton.edu/exchange/Attach/orb-x.html#RL23_DROME) |  | [32.9](http://gemini.scripps.edu/cgi-bin/SeqCov?/wfs/bfd/3/ian/fly/DrosMelano7227.FASTAC.txt&RL23_DROME&16+31^52+15&36+11*) | (P48159) 60S ribosomal protein L23 (L17A) |
| RL7_DROME |  | [23.8](http://molbio.princeton.edu/exchange/Attach/orb-x.html#RL7_DROME) |  | [23.8](http://gemini.scripps.edu/cgi-bin/SeqCov?/wfs/bfd/3/ian/fly/DrosMelano7227.FASTAC.txt&RL7_DROME&138+14^168+13^184+33&170+11*) | (P32100) 60S ribosomal protein L7 |
| RNT1_DROME |  | [44.2](http://molbio.princeton.edu/exchange/Attach/orb-x.html#RNT1_DROME) |  | [44.2](http://gemini.scripps.edu/cgi-bin/SeqCov?/wfs/bfd/3/ian/fly/DrosMelano7227.FASTAC.txt&RNT1_DROME&37+22^102+17^144+44^198+24^248+25^341+33^382+12^412+10^428+10^443+12^467+48^523+25^582+24^637+18^715+33^763+50^846+12^862+12^876+12^1040+13^1056+25^1098+40&167+11^198+12^248+12^341+19^412+10^443+12^467+13^493+22^531+17^715+10^729+19^848+10^1056+25&198+10^349+11^729+19&350+10*) | (Q9VYS3) Regulator of nonsense transcripts 1 homolog |
| RS12_DROME |  | [51.1](http://molbio.princeton.edu/exchange/Attach/orb-x.html#RS12_DROME) |  | [51.1](http://gemini.scripps.edu/cgi-bin/SeqCov?/wfs/bfd/3/ian/fly/DrosMelano7227.FASTAC.txt&RS12_DROME&32+16^53+17^71+15^91+10^120+13&34+14&35+13*) | (P80455) 40S ribosomal protein S12 |
| RS13_DROME |  | [18.0](http://molbio.princeton.edu/exchange/Attach/orb-x.html#RS13_DROME) |  | [18.0](http://gemini.scripps.edu/cgi-bin/SeqCov?/wfs/bfd/3/ian/fly/DrosMelano7227.FASTAC.txt&RS13_DROME&9+10^76+17*) | (Q03334) 40S ribosomal protein S13 |
| RS17_DROME |  | [43.1](http://molbio.princeton.edu/exchange/Attach/orb-x.html#RS17_DROME) |  | [43.1](http://gemini.scripps.edu/cgi-bin/SeqCov?/wfs/bfd/3/ian/fly/DrosMelano7227.FASTAC.txt&RS17_DROME&32+15^49+11^81+22^107+8&33+14^49+11*) | (P17704) 40S ribosomal protein S17 |
| RS1A_DROME |  | [49.6](http://molbio.princeton.edu/exchange/Attach/orb-x.html#RS1A_DROME) |  | [49.6](http://gemini.scripps.edu/cgi-bin/SeqCov?/wfs/bfd/3/ian/fly/DrosMelano7227.FASTAC.txt&RS1A_DROME&3+9^32+25^78+10^97+20*) | (P48149) 40S ribosomal protein S15A |
| RS26_DROME |  | [23.7](http://molbio.princeton.edu/exchange/Attach/orb-x.html#RS26_DROME) |  | [23.7](http://gemini.scripps.edu/cgi-bin/SeqCov?/wfs/bfd/3/ian/fly/DrosMelano7227.FASTAC.txt&RS26_DROME&52+15^71+12*) | (P13008) 40S ribosomal protein S26 (DS31) |
| RS3_DROME |  | [29.3](http://molbio.princeton.edu/exchange/Attach/orb-x.html#RS3_DROME) |  | [29.3](http://gemini.scripps.edu/cgi-bin/SeqCov?/wfs/bfd/3/ian/fly/DrosMelano7227.FASTAC.txt&RS3_DROME&13+8^43+14^154+22^204+13^230+15*) | (Q06559) 40S ribosomal protein S3 |
| SSRP_DROME |  | [4.4](http://molbio.princeton.edu/exchange/Attach/orb-x.html#SSRP_DROME) |  | [4.4](http://gemini.scripps.edu/cgi-bin/SeqCov?/wfs/bfd/3/ian/fly/DrosMelano7227.FASTAC.txt&SSRP_DROME&218+11^326+21*) | (Q05344) Single-strand recognition protein (SSRP) (Chorion-factor 5) |
| TBB3_DROME |  | [25.3](http://molbio.princeton.edu/exchange/Attach/orb-x.html#TBB3_DROME) |  | [25.3](http://gemini.scripps.edu/cgi-bin/SeqCov?/wfs/bfd/3/ian/fly/DrosMelano7227.FASTAC.txt&TBB3_DROME&3+17^84+26^140+21^169+12^259+24^289+15&269+14*) | (P08841) Tubulin beta-3 chain |
| U202_DROME |  | [4.1](http://molbio.princeton.edu/exchange/Attach/orb-x.html#U202_DROME) |  | [4.1](http://gemini.scripps.edu/cgi-bin/SeqCov?/wfs/bfd/3/ian/fly/DrosMelano7227.FASTAC.txt&U202_DROME&51+10^82+13^252+18*) | (Q9W3C1) Polycomb protein CG1994 (p110 protein) |
| **132** |  | **X** |  |  |  |

| ***Locus*** | ***dorsal*** | ***orb*** | ***rin*** | ***Total*** | ***Description*** |
| --- | --- | --- | --- | --- | --- |
| AAF54965 |  | [47.7](http://molbio.princeton.edu/exchange/Attach/orb-x.html#AAF54965) | [48.7](http://molbio.princeton.edu/exchange/Attach/rin-x.html#AAF54965) | [56.2](http://gemini.scripps.edu/cgi-bin/SeqCov?/wfs/bfd/3/ian/fly/DrosMelano7227.FASTAC.txt&AAF54965&2+17^38+14^63+176^322+28^384+43^482+42^528+46^583+22&2+17^38+14^63+81^179+40^324+26^391+33^482+42^538+36^591+14&38+14^75+54^190+29^332+18^400+19^482+29^516+8^538+18^564+10&38+14^85+44^192+27^341+9^482+29^543+13&42+10^87+18^106+14^199+20^341+9^483+28^545+11*) | (AAF54965) CG9412-PB --*rasputiin* |
| AAS65389 |  | [4.5](http://molbio.princeton.edu/exchange/Attach/orb-x.html#AAS65389) | [2.9](http://molbio.princeton.edu/exchange/Attach/rin-x.html#AAS65389) | [7.4](http://gemini.scripps.edu/cgi-bin/SeqCov?/wfs/bfd/3/ian/fly/DrosMelano7227.FASTAC.txt&AAS65389&227+29^532+26^566+37^1327+11*) | (AAS65389) CG18572-PB -- *rudimentary* |
| HS7E_DROME |  | [4.1](http://molbio.princeton.edu/exchange/Attach/orb-x.html#HS7E_DROME) | [14.8](http://molbio.princeton.edu/exchange/Attach/rin-x.html#HS7E_DROME) | [18.9](http://gemini.scripps.edu/cgi-bin/SeqCov?/wfs/bfd/3/ian/fly/DrosMelano7227.FASTAC.txt&HS7E_DROME&52+21^194+9^239+21^317+29^378+14^469+17^577+19*) | (P29845) *Heat shock 70 kDa protein cognate 5* |
| PABP_DROME |  | [18.8](http://molbio.princeton.edu/exchange/Attach/orb-x.html#PABP_DROME) | [24.1](http://molbio.princeton.edu/exchange/Attach/rin-x.html#PABP_DROME) | [37.8](http://gemini.scripps.edu/cgi-bin/SeqCov?/wfs/bfd/3/ian/fly/DrosMelano7227.FASTAC.txt&PABP_DROME&42+28^105+16^222+23^354+9^424+36^467+20^492+26^529+20^553+10^574+39^615+12&42+17^222+23^467+20^529+20^574+15*) | (P21187) *Polyadenylate-binding protein (Poly(A)-binding protein) (PABP)* |
| PPOL_DROME  Q7PLT6 |  | [22.1](http://molbio.princeton.edu/exchange/Attach/orb-x.html#PPOL_DROME) | [5.8](http://molbio.princeton.edu/exchange/Attach/rin-x.html#PPOL_DROME) | [26.1](http://gemini.scripps.edu/cgi-bin/SeqCov?/wfs/bfd/3/ian/fly/DrosMelano7227.FASTAC.txt&PPOL_DROME&58+17^80+19^150+42^298+14^332+10^440+42^536+12^622+13^718+27^762+19^789+13^848+13^947+18&58+17^80+19^150+17^848+13*)  [26.1](http://gemini.scripps.edu/cgi-bin/SeqCov?/wfs/bfd/3/ian/fly/DrosMelano7227.FASTAC.txt&Q7PLT6&58+17^80+19^150+42^298+14^332+10^440+42^536+12^622+13^718+27^762+19^789+13^848+13^947+18&58+17^80+19^150+17^848+13*) | (P35875) *Poly [ADP-ribose] polymerase (EC 2.4.2.30) (PARP) (ADPRT) (NAD(+) ADP-ribosyltransferase) (Poly[ADP-ribose] synthetase)*  (Q7PLT6) CG40411-PC -- *Poly-(ADP-ribose) polymerase (nucleolus and CNS)* |
| Q8INE0  Q9VF64  Q8SWR8 |  | [3.3](http://molbio.princeton.edu/exchange/Attach/orb-x.html#Q8INE0) | [17.7](http://molbio.princeton.edu/exchange/Attach/rin-x.html#Q8INE0) | [19.6](http://gemini.scripps.edu/cgi-bin/SeqCov?/wfs/bfd/3/ian/fly/DrosMelano7227.FASTAC.txt&Q8INE0&28+17^57+36^102+31^134+31^265+19^289+32^761+34&57+15^134+24&134+24*)  [22.5](http://gemini.scripps.edu/cgi-bin/SeqCov?/wfs/bfd/3/ian/fly/DrosMelano7227.FASTAC.txt&Q9VF64&28+17^57+36^102+31^134+31^265+19^289+32^761+34&57+15^134+24&134+24*)  [18.5](http://gemini.scripps.edu/cgi-bin/SeqCov?/wfs/bfd/3/ian/fly/DrosMelano7227.FASTAC.txt&Q8SWR8&89+17^118+36^163+31^195+31^326+19^350+32^822+34&118+15^195+24&195+24*) | (Q8INE0) CG5166-PC -- *Ataxin-2 (RNA binding ptn expressed in CNS)*  (Q9VF64) CG5166-PA -- *Ataxin-2*  (Q8SWR8) GH13857p (CG5166-PB) -- *Ataxin-2* |
| Q8INM7 |  | [33.4](http://molbio.princeton.edu/exchange/Attach/orb-x.html#Q8INM7) | [8.2](http://molbio.princeton.edu/exchange/Attach/rin-x.html#Q8INM7) | [33.4](http://gemini.scripps.edu/cgi-bin/SeqCov?/wfs/bfd/3/ian/fly/DrosMelano7227.FASTAC.txt&Q8INM7&35+15^61+15^89+19^117+28^190+34^229+11^242+14^296+40^365+10^410+9^616+20&35+15^128+10^190+34^296+37^365+10^616+20&39+11^128+10^192+19^296+37&39+11^303+24&303+24*) | (Q8INM7) CG6203-PE –*Fmr1* |
| Q8MKQ2  Q9V358 |  | [41.3](http://molbio.princeton.edu/exchange/Attach/orb-x.html#Q8MKQ2) | [23.7](http://molbio.princeton.edu/exchange/Attach/rin-x.html#Q8MKQ2) | [41.3](http://gemini.scripps.edu/cgi-bin/SeqCov?/wfs/bfd/3/ian/fly/DrosMelano7227.FASTAC.txt&Q8MKQ2&22+26^72+61^141+24^281+46^434+38^541+27^579+153^818+37^1056+55^1144+61^1217+76&72+61^141+24^281+46^444+28^541+27^596+123^827+28^1058+53^1146+59^1217+36^1256+35&89+44^281+46^448+24^541+27^642+77^836+19^1075+36^1147+58^1217+24&99+34^305+22^448+24^541+27^651+43^836+19^1077+19^1157+48^1217+23&120+13^449+23^543+25^652+20^681+13^1078+18^1159+22^1183+22^1217+23*)  [41.0](http://gemini.scripps.edu/cgi-bin/SeqCov?/wfs/bfd/3/ian/fly/DrosMelano7227.FASTAC.txt&Q9V358&22+26^72+61^141+24^281+46^434+38^541+27^579+153^818+37^1056+55^1144+61^1217+76&72+61^141+24^281+46^444+28^541+27^596+123^827+28^1058+53^1146+59^1217+36^1256+35&89+44^281+46^448+24^541+27^642+77^836+19^1075+36^1147+58^1217+24&99+34^305+22^448+24^541+27^651+43^836+19^1077+19^1157+48^1217+23&120+13^449+23^543+25^652+20^681+13^1078+18^1159+22^1183+22^1217+23*) | (Q8MKQ2) CG8715-PB --*lingerer*  (Q9V358) CG8715 protein --*lingerer* |
| Q95P21 |  | [41.2](http://molbio.princeton.edu/exchange/Attach/orb-x.html#Q95P21) | [10.6](http://molbio.princeton.edu/exchange/Attach/rin-x.html#Q95P21) | [41.2](http://gemini.scripps.edu/cgi-bin/SeqCov?/wfs/bfd/3/ian/fly/DrosMelano7227.FASTAC.txt&Q95P21&18+23^73+15^99+15^127+19^155+28^228+34^267+11^280+14^327+40^396+10^441+9&18+23^73+15^166+10^228+34^327+37^396+10&77+11^166+10^230+19^327+37&77+11^334+24&334+24*) | (Q95P21) *Fragile X related protein* (CG6203-PB) |
| Q9I7J5 |  | [45.3](http://molbio.princeton.edu/exchange/Attach/orb-x.html#Q9I7J5) | [46.4](http://molbio.princeton.edu/exchange/Attach/rin-x.html#Q9I7J5) | [53.9](http://gemini.scripps.edu/cgi-bin/SeqCov?/wfs/bfd/3/ian/fly/DrosMelano7227.FASTAC.txt&Q9I7J5&36+14^61+176^320+28^382+43^480+42^526+46^581+22&36+14^61+81^177+40^322+26^389+33^480+42^536+36^589+14&36+14^73+54^188+29^330+18^398+19^480+29^514+8^536+18^562+10&36+14^83+44^190+27^339+9^480+29^541+13&40+10^85+18^104+14^197+20^339+9^481+28^543+11*) | (Q9I7J5) CG9412-PA --*rasputin* |
| Q9NFU0  Q9TVY4 |  | [34.8](http://molbio.princeton.edu/exchange/Attach/orb-x.html#Q9NFU0) | [11.1](http://molbio.princeton.edu/exchange/Attach/rin-x.html#Q9NFU0) | [34.8](http://gemini.scripps.edu/cgi-bin/SeqCov?/wfs/bfd/3/ian/fly/DrosMelano7227.FASTAC.txt&Q9NFU0&18+23^73+15^99+15^127+19^155+28^228+34^267+11^280+14^337+40^406+10^451+9^657+20&18+23^73+15^166+10^228+34^337+37^406+10^657+20&77+11^166+10^230+19^337+37&77+11^344+24&344+24*)  [34.9](http://gemini.scripps.edu/cgi-bin/SeqCov?/wfs/bfd/3/ian/fly/DrosMelano7227.FASTAC.txt&Q9TVY4&18+23^73+15^99+15^127+19^155+28^228+34^267+11^280+14^334+40^403+10^448+9^654+20&18+23^73+15^166+10^228+34^334+37^403+10^654+20&77+11^166+10^230+19^334+37&77+11^341+24&341+24*) | (Q9NFU0) *Fragile X related protein* (CG6203-PA)  (Q9TVY4) *Fragile X related* (CG6203-PC) |
| Q9V631 |  | [36.1](http://molbio.princeton.edu/exchange/Attach/orb-x.html#Q9V631) | [11.4](http://molbio.princeton.edu/exchange/Attach/rin-x.html#Q9V631) | [42.3](http://gemini.scripps.edu/cgi-bin/SeqCov?/wfs/bfd/3/ian/fly/DrosMelano7227.FASTAC.txt&Q9V631&85+12^101+29^135+12^180+33^227+18^256+35^323+8^336+36^396+13&181+32^227+18^267+24^336+32*) | (Q9V631) CG8280 protein --*Elongation factor 1α48D* |
| Q9V8D6 |  | [56.1](http://molbio.princeton.edu/exchange/Attach/orb-x.html#Q9V8D6) | [36.8](http://molbio.princeton.edu/exchange/Attach/rin-x.html#Q9V8D6) | [59.7](http://gemini.scripps.edu/cgi-bin/SeqCov?/wfs/bfd/3/ian/fly/DrosMelano7227.FASTAC.txt&Q9V8D6&21+36^59+44^105+26^167+13^186+14^202+32^237+14^255+11^267+11^285+125^424+31^526+19^612+26^676+18^716+21^751+16&59+44^105+26^167+13^186+14^202+20^237+14^267+11^295+67^366+44^526+19^677+17^717+20^751+16&186+14^202+20^391+19^677+17&202+20^391+19&391+11*) | (Q9V8D6) CG5726 protein (RE28271p) |
| Q9V9M7 |  | [17.0](http://molbio.princeton.edu/exchange/Attach/orb-x.html#Q9V9M7) | [10.1](http://molbio.princeton.edu/exchange/Attach/rin-x.html#Q9V9M7) | [17.0](http://gemini.scripps.edu/cgi-bin/SeqCov?/wfs/bfd/3/ian/fly/DrosMelano7227.FASTAC.txt&Q9V9M7&22+11^64+16&64+16&64+16&64+15&64+15*) | (Q9V9M7) *Ribosomal protein L21* (RE62581p) (RH06526p) |
| Q9VLK2 |  | [6.9](http://molbio.princeton.edu/exchange/Attach/orb-x.html#Q9VLK2) | [6.0](http://molbio.princeton.edu/exchange/Attach/rin-x.html#Q9VLK2) | [12.9](http://gemini.scripps.edu/cgi-bin/SeqCov?/wfs/bfd/3/ian/fly/DrosMelano7227.FASTAC.txt&Q9VLK2&33+14^80+19^119+15^332+13^454+27&80+19^119+15*) | (Q9VLK2) CG13096 protein |
| Q9VXD5 |  | [3.6](http://molbio.princeton.edu/exchange/Attach/orb-x.html#Q9VXD5) | [1.8](http://molbio.princeton.edu/exchange/Attach/rin-x.html#Q9VXD5) | [5.4](http://gemini.scripps.edu/cgi-bin/SeqCov?/wfs/bfd/3/ian/fly/DrosMelano7227.FASTAC.txt&Q9VXD5&227+29^532+26^566+37^1327+11^1870+18*) | (Q9VXD5) CG18572 protein --*rudimentary* |
| Q9W229 |  | [20.6](http://molbio.princeton.edu/exchange/Attach/orb-x.html#Q9W229) | [20.6](http://molbio.princeton.edu/exchange/Attach/rin-x.html#Q9W229) | [20.6](http://gemini.scripps.edu/cgi-bin/SeqCov?/wfs/bfd/3/ian/fly/DrosMelano7227.FASTAC.txt&Q9W229&51+12^70+15&51+12^70+15*) | (Q9W229) CG3751 protein (RE59324p) --*Ribosomal protein S24* |
| Q9W4M7 |  | [1.7](http://molbio.princeton.edu/exchange/Attach/orb-x.html#Q9W4M7) | [23.0](http://molbio.princeton.edu/exchange/Attach/rin-x.html#Q9W4M7) | [23.0](http://gemini.scripps.edu/cgi-bin/SeqCov?/wfs/bfd/3/ian/fly/DrosMelano7227.FASTAC.txt&Q9W4M7&323+21^414+21^499+14^526+11^539+23^563+63^740+27^778+14^928+29^968+22^998+21^1170+21^1340+30^1458+21^1519+12^1545+31^1581+24^1750+34&323+21^414+21^526+11^539+23^564+62^968+22^998+21^1170+21^1458+21^1581+24^1762+22&1172+19^1458+21^1582+23&1582+23*) | (Q9W4M7) CG4857 protein |
| R13A_DROME |  | [17.1](http://molbio.princeton.edu/exchange/Attach/orb-x.html#R13A_DROME) | [12.7](http://molbio.princeton.edu/exchange/Attach/rin-x.html#R13A_DROME) | [17.1](http://gemini.scripps.edu/cgi-bin/SeqCov?/wfs/bfd/3/ian/fly/DrosMelano7227.FASTAC.txt&R13A_DROME&40+12^119+9^143+14&40+12^143+14&143+14*) | (Q9VNE9) *60S ribosomal protein L13A* |
| RL10_DROME |  | [19.3](http://molbio.princeton.edu/exchange/Attach/orb-x.html#RL10_DROME) | [13.8](http://molbio.princeton.edu/exchange/Attach/rin-x.html#RL10_DROME) | [19.7](http://gemini.scripps.edu/cgi-bin/SeqCov?/wfs/bfd/3/ian/fly/DrosMelano7227.FASTAC.txt&RL10_DROME&40+30^199+13&41+29^199+13&41+29*) | (O61231) *60S ribosomal protein L10* (QM protein homolog) (dQM) |
| RL11_DROME |  | [12.5](http://molbio.princeton.edu/exchange/Attach/orb-x.html#RL11_DROME) | [16.8](http://molbio.princeton.edu/exchange/Attach/rin-x.html#RL11_DROME) | [29.3](http://gemini.scripps.edu/cgi-bin/SeqCov?/wfs/bfd/3/ian/fly/DrosMelano7227.FASTAC.txt&RL11_DROME&25+13^44+14^124+18^175+9&175+9*) | (P46222) *60S ribosomal protein L11* |
| RS14_DROME |  | [37.1](http://molbio.princeton.edu/exchange/Attach/orb-x.html#RS14_DROME) | [28.5](http://molbio.princeton.edu/exchange/Attach/rin-x.html#RS14_DROME) | [57.6](http://gemini.scripps.edu/cgi-bin/SeqCov?/wfs/bfd/3/ian/fly/DrosMelano7227.FASTAC.txt&RS14_DROME&8+43^64+21^87+10^129+13&11+12^67+18^87+10&11+12*) | (P14130) *40S ribosomal protein S14* |
| RS2_DROME |  | [30.0](http://molbio.princeton.edu/exchange/Attach/orb-x.html#RS2_DROME) | [19.1](http://molbio.princeton.edu/exchange/Attach/rin-x.html#RS2_DROME) | [36.3](http://gemini.scripps.edu/cgi-bin/SeqCov?/wfs/bfd/3/ian/fly/DrosMelano7227.FASTAC.txt&RS2_DROME&60+32^109+17^196+15^230+33&73+19^196+15^230+11&73+14&73+14*) | (P31009) *40S ribosomal protein S2* (Strings of pearls protein) |
| **23** |  | **X** | **X** |  |  |

| ***Locus*** | ***dorsal*** | ***orb*** | ***rin*** | ***Total*** | ***Description*** |
| --- | --- | --- | --- | --- | --- |
| AAF46547  VIT2_DROME | [29.8](http://molbio.princeton.edu/exchange/Attach/dorsal-x.html#AAF46547) | [47.5](http://molbio.princeton.edu/exchange/Attach/orb-x.html#AAF46547) | [32.5](http://molbio.princeton.edu/exchange/Attach/rin-x.html#AAF46547) | [52.3](http://gemini.scripps.edu/cgi-bin/SeqCov?/wfs/bfd/3/ian/fly/DrosMelano7227.FASTAC.txt&AAF46547&48+26^79+39^128+11^145+22^219+26^253+32^319+48^382+14^404+22&48+17^79+39^128+11^145+22^219+26^319+48^382+14&79+16^97+21^128+11^145+22^219+26^319+21^382+14&79+16^102+16^128+11^145+22^219+26^319+21&80+15^103+15^145+22^219+26^319+21*)  [54.3](http://gemini.scripps.edu/cgi-bin/SeqCov?/wfs/bfd/3/ian/fly/DrosMelano7227.FASTAC.txt&VIT2_DROME&31+26^62+39^111+11^128+22^202+26^236+32^302+48^365+14^387+22&31+17^62+39^111+11^128+22^202+26^302+48^365+14&62+16^80+21^111+11^128+22^202+26^302+21^365+14&62+16^85+16^111+11^128+22^202+26^302+21&63+15^86+15^128+22^202+26^302+21*) | (AAF46547) CG2979-PA  (P02844) Vitellogenin II precursor (Yolk protein 2) |
| AAF49905  Q9VTZ0 | [22.7](http://molbio.princeton.edu/exchange/Attach/dorsal-x.html#AAF49905) | [39.9](http://molbio.princeton.edu/exchange/Attach/orb-x.html#AAF49905) | [10.3](http://molbio.princeton.edu/exchange/Attach/rin-x.html#AAF49905) | [41.9](http://gemini.scripps.edu/cgi-bin/SeqCov?/wfs/bfd/3/ian/fly/DrosMelano7227.FASTAC.txt&AAF49905&26+43^77+19^159+42^223+27^388+11^401+20^426+30^469+26^497+14^523+13^543+28&26+43^173+28^224+26^401+20^428+28^470+25^497+14^523+13^543+24&26+17^50+19^225+25^401+20^428+28^470+25^524+12&50+19^226+24^401+20^428+28^524+12&50+19^401+20^428+28^524+12*)  [40.1](http://gemini.scripps.edu/cgi-bin/SeqCov?/wfs/bfd/3/ian/fly/DrosMelano7227.FASTAC.txt&Q9VTZ0&26+43^77+19^159+42^223+27^388+11^401+20^426+30^469+26^497+14^523+13^543+28&26+43^173+28^224+26^401+20^428+28^470+25^497+14^523+13^543+24&26+17^50+19^225+25^401+20^428+28^470+25^524+12&50+19^226+24^401+20^428+28^524+12&50+19^401+20^428+28^524+12*) | (AAF49905) CG10686-PA  (Q9VTZ0) CG10686 protein |
| ACT1_DROME | [19.7](http://molbio.princeton.edu/exchange/Attach/dorsal-x.html#ACT1_DROME) | [40.4](http://molbio.princeton.edu/exchange/Attach/orb-x.html#ACT1_DROME) | [19.7](http://molbio.princeton.edu/exchange/Attach/rin-x.html#ACT1_DROME) | [46.0](http://gemini.scripps.edu/cgi-bin/SeqCov?/wfs/bfd/3/ian/fly/DrosMelano7227.FASTAC.txt&ACT1_DROME&30+11^86+29^149+30^185+8^217+23^244+12^258+28^293+21^317+11&86+29^149+30^217+23^293+21&86+11^149+30^217+23&149+26^217+23&149+22^217+23*) | (P10987) Actin-5C |
| ACT2_DROME | [19.7](http://molbio.princeton.edu/exchange/Attach/dorsal-x.html#ACT2_DROME) | [33.0](http://molbio.princeton.edu/exchange/Attach/orb-x.html#ACT2_DROME) | [19.7](http://molbio.princeton.edu/exchange/Attach/rin-x.html#ACT2_DROME) | [38.6](http://gemini.scripps.edu/cgi-bin/SeqCov?/wfs/bfd/3/ian/fly/DrosMelano7227.FASTAC.txt&ACT2_DROME&30+11^86+29^149+30^185+8^217+23^244+12^293+21^317+11&86+29^149+30^217+23^293+21&86+11^149+30^217+23&149+26^217+23&149+22^217+23*) | (P02572) Actin-42A |
| ACT3_DROME  ACT5_DROME | [19.7](http://molbio.princeton.edu/exchange/Attach/dorsal-x.html#ACT3_DROME) | [28.2](http://molbio.princeton.edu/exchange/Attach/orb-x.html#ACT3_DROME) | [19.7](http://molbio.princeton.edu/exchange/Attach/rin-x.html#ACT3_DROME) | [39.9](http://gemini.scripps.edu/cgi-bin/SeqCov?/wfs/bfd/3/ian/fly/DrosMelano7227.FASTAC.txt&ACT3_DROME&30+11^70+45^149+30^185+8^217+23^244+12^293+21&86+29^149+30^217+23^293+21&86+11^149+30^217+23&149+26^217+23&149+22*)  [39.9](http://gemini.scripps.edu/cgi-bin/SeqCov?/wfs/bfd/3/ian/fly/DrosMelano7227.FASTAC.txt&ACT5_DROME&30+11^70+45^149+30^185+8^217+23^244+12^293+21&86+29^149+30^217+23^293+21&86+11^149+30^217+23&149+26^217+23&149+22*) | (P53501) Actin 57B  (P10981) Actin-87E |
| ACT4_DROME | [14.1](http://molbio.princeton.edu/exchange/Attach/dorsal-x.html#ACT4_DROME) | [25.3](http://molbio.princeton.edu/exchange/Attach/orb-x.html#ACT4_DROME) | [14.1](http://molbio.princeton.edu/exchange/Attach/rin-x.html#ACT4_DROME) | [31.4](http://gemini.scripps.edu/cgi-bin/SeqCov?/wfs/bfd/3/ian/fly/DrosMelano7227.FASTAC.txt&ACT4_DROME&30+11^70+16^97+18^149+30^185+8^217+23^244+12&97+18^149+30^217+23&149+30^217+23&149+26^217+23&149+22*) | (P02574) Actin, larval muscle (Actin-79B) |
| ACT6_DROME | [6.1](http://molbio.princeton.edu/exchange/Attach/dorsal-x.html#ACT6_DROME) | [20.2](http://molbio.princeton.edu/exchange/Attach/orb-x.html#ACT6_DROME) | [6.1](http://molbio.princeton.edu/exchange/Attach/rin-x.html#ACT6_DROME) | [26.3](http://gemini.scripps.edu/cgi-bin/SeqCov?/wfs/bfd/3/ian/fly/DrosMelano7227.FASTAC.txt&ACT6_DROME&30+11^70+45^185+8^217+23^244+12&86+29^217+23&86+11^217+23&217+23*) | (P02575) Actin, indirect flight muscle (Actin-88F) |
| ADT_DROME  Q8IRA0 | [29.8](http://molbio.princeton.edu/exchange/Attach/dorsal-x.html#ADT_DROME) | [48.8](http://molbio.princeton.edu/exchange/Attach/orb-x.html#ADT_DROME) | [25.4](http://molbio.princeton.edu/exchange/Attach/rin-x.html#ADT_DROME) | [53.2](http://gemini.scripps.edu/cgi-bin/SeqCov?/wfs/bfd/3/ian/fly/DrosMelano7227.FASTAC.txt&ADT_DROME&4+22^36+10^83+12^114+27^154+11^168+33^246+28^282+16&4+22^36+10^85+10^114+27^190+11^254+11^282+16&4+22^85+10^114+27^190+11^254+11^282+16&4+22^119+22^282+15&282+15*)  [51.0](http://gemini.scripps.edu/cgi-bin/SeqCov?/wfs/bfd/3/ian/fly/DrosMelano7227.FASTAC.txt&Q8IRA0&17+22^49+10^96+12^127+27^167+11^181+33^259+28^295+16&17+22^49+10^98+10^127+27^203+11^267+11^295+16&17+22^98+10^127+27^203+11^267+11^295+16&17+22^132+22^295+15&295+15*) | (Q26365) ADP,ATP carrier protein (ADP/ATP translocase) (Adenine nucleotide translocator) (ANT) (Stress sensitive B protein)  (Q8IRA0) CG16944-PC |
| ATPA_DROME | [13.4](http://molbio.princeton.edu/exchange/Attach/dorsal-x.html#ATPA_DROME) | [17.2](http://molbio.princeton.edu/exchange/Attach/orb-x.html#ATPA_DROME) | [5.8](http://molbio.princeton.edu/exchange/Attach/rin-x.html#ATPA_DROME) | [23.0](http://gemini.scripps.edu/cgi-bin/SeqCov?/wfs/bfd/3/ian/fly/DrosMelano7227.FASTAC.txt&ATPA_DROME&47+11^83+40^133+16^218+12^402+14^441+22^516+12&83+40^218+12^441+22*) | (P35381) ATP synthase alpha chain, mitochondrial precursor (EC 3.6.3.14) (Protein bellwether) |
| ATPB_DROME | [23.0](http://molbio.princeton.edu/exchange/Attach/dorsal-x.html#ATPB_DROME) | [15.2](http://molbio.princeton.edu/exchange/Attach/orb-x.html#ATPB_DROME) | [6.5](http://molbio.princeton.edu/exchange/Attach/rin-x.html#ATPB_DROME) | [35.2](http://gemini.scripps.edu/cgi-bin/SeqCov?/wfs/bfd/3/ian/fly/DrosMelano7227.FASTAC.txt&ATPB_DROME&72+15^121+12^190+13^219+18^259+13^288+14^328+56^410+19^440+18&72+15^219+18^329+36&72+15*) | (Q05825) ATP synthase beta chain, mitochondrial precursor (EC 3.6.3.14) |
| CH60_DROME | [13.1](http://molbio.princeton.edu/exchange/Attach/dorsal-x.html#CH60_DROME) | [5.4](http://molbio.princeton.edu/exchange/Attach/orb-x.html#CH60_DROME) | [29.0](http://molbio.princeton.edu/exchange/Attach/rin-x.html#CH60_DROME) | [32.1](http://gemini.scripps.edu/cgi-bin/SeqCov?/wfs/bfd/3/ian/fly/DrosMelano7227.FASTAC.txt&CH60_DROME&34+21^93+25^139+10^202+13^233+13^247+40^307+23^335+14^523+25&34+21^93+25^202+13^233+13^307+23^335+14^523+25*) | (O02649) 60 kDa heat shock protein, mitochondrial precursor (Hsp60) (60 kDa chaperonin) (CPN60) (Heat shock protein 60) (HSP-60) (Mitochondrial matrix protein P1) |
| CUP_DROME | [9.7](http://molbio.princeton.edu/exchange/Attach/dorsal-x.html#CUP_DROME) | [29.5](http://molbio.princeton.edu/exchange/Attach/orb-x.html#CUP_DROME) | [8.7](http://molbio.princeton.edu/exchange/Attach/rin-x.html#CUP_DROME) | [34.0](http://gemini.scripps.edu/cgi-bin/SeqCov?/wfs/bfd/3/ian/fly/DrosMelano7227.FASTAC.txt&CUP_DROME&65+22^181+24^352+10^371+12^401+22^425+32^496+32^552+13^578+31^730+21^766+17^805+36^884+11^921+60^1057+22^1113+20&65+22^496+19^553+12^594+15^730+21^884+11^921+31^1057+22^1119+14&67+20^496+19^730+21^921+31&496+19*) | (Q9VMA3) Cup protein (Oskar ribonucleoprotein complex 147 kDa subunit) |
| FKB4_DROME | [7.6](http://molbio.princeton.edu/exchange/Attach/dorsal-x.html#FKB4_DROME) | [22.4](http://molbio.princeton.edu/exchange/Attach/orb-x.html#FKB4_DROME) | [7.6](http://molbio.princeton.edu/exchange/Attach/rin-x.html#FKB4_DROME) | [22.4](http://gemini.scripps.edu/cgi-bin/SeqCov?/wfs/bfd/3/ian/fly/DrosMelano7227.FASTAC.txt&FKB4_DROME&22+11^55+13^271+9^298+20^323+13^341+14&22+11^55+13^298+10^341+14&55+13^341+14*) | (P54397) 39 kDa FK506-binding nuclear protein (EC 5.2.1.8) (Peptidyl-prolyl cis-trans isomerase) (PPIase) (Rotamase) |
| HS7A_DROME | [6.1](http://molbio.princeton.edu/exchange/Attach/dorsal-x.html#HS7A_DROME) | [7.5](http://molbio.princeton.edu/exchange/Attach/orb-x.html#HS7A_DROME) | [6.1](http://molbio.princeton.edu/exchange/Attach/rin-x.html#HS7A_DROME) | [7.5](http://gemini.scripps.edu/cgi-bin/SeqCov?/wfs/bfd/3/ian/fly/DrosMelano7227.FASTAC.txt&HS7A_DROME&147+9^172+16^362+23&172+16^362+23&172+16^362+23&176+12^362+23&180+8^364+21*) | (P29843) Heat shock 70 kDa protein cognate 1 (Heat shock 70 kDa protein 70C) |
| HS7C_DROME | [10.4](http://molbio.princeton.edu/exchange/Attach/dorsal-x.html#HS7C_DROME) | [33.1](http://molbio.princeton.edu/exchange/Attach/orb-x.html#HS7C_DROME) | [16.3](http://molbio.princeton.edu/exchange/Attach/rin-x.html#HS7C_DROME) | [40.2](http://gemini.scripps.edu/cgi-bin/SeqCov?/wfs/bfd/3/ian/fly/DrosMelano7227.FASTAC.txt&HS7C_DROME&27+21^62+14^103+12^124+29^164+18^186+28^307+18^327+10^354+14^448+27^563+11^592+27^622+35&27+21^62+14^127+12^165+17^198+16^307+18^355+13^563+11^592+12^622+12&165+17^198+16^307+18^355+13^592+12&202+12^594+10&206+8*) | (P29844) Heat shock 70 kDa protein cognate 3 precursor (78 kDa glucose regulated protein homolog) (GRP 78) (Heat shock protein cognate 72) |
| HS7D_DROME | [29.5](http://molbio.princeton.edu/exchange/Attach/dorsal-x.html#HS7D_DROME) | [46.1](http://molbio.princeton.edu/exchange/Attach/orb-x.html#HS7D_DROME) | [39.0](http://molbio.princeton.edu/exchange/Attach/rin-x.html#HS7D_DROME) | [58.1](http://gemini.scripps.edu/cgi-bin/SeqCov?/wfs/bfd/3/ian/fly/DrosMelano7227.FASTAC.txt&HS7D_DROME&4+22^37+13^57+15^78+25^113+14^138+18^160+28^194+27^237+10^273+39^329+14^349+67^424+24^459+35^540+11^580+16&4+22^37+13^78+25^113+14^147+9^160+28^237+10^273+27^302+10^329+14^349+9^362+54^424+24^540+11&4+22^39+11^91+12^115+12^160+28^237+10^273+27^302+10^329+14^352+6^362+54^424+24&4+22^116+11^160+12^176+12^302+10^362+23^400+16^424+24&4+22^180+8^302+10^362+23^400+16^424+24*) | (P11147) Heat shock 70 kDa protein cognate 4 (Heat shock 70 kDa protein 88E) |
| KPYK_DROME | [12.9](http://molbio.princeton.edu/exchange/Attach/dorsal-x.html#KPYK_DROME) | [13.3](http://molbio.princeton.edu/exchange/Attach/orb-x.html#KPYK_DROME) | [5.4](http://molbio.princeton.edu/exchange/Attach/rin-x.html#KPYK_DROME) | [21.6](http://gemini.scripps.edu/cgi-bin/SeqCov?/wfs/bfd/3/ian/fly/DrosMelano7227.FASTAC.txt&KPYK_DROME&36+11^124+15^177+13^194+34^346+25^387+17&177+13^194+16^346+25*) | (O62619) Pyruvate kinase (EC 2.7.1.40) (PK) |
| ME31_DROME | [41.0](http://molbio.princeton.edu/exchange/Attach/dorsal-x.html#ME31_DROME) | [41.6](http://molbio.princeton.edu/exchange/Attach/orb-x.html#ME31_DROME) | [36.4](http://molbio.princeton.edu/exchange/Attach/rin-x.html#ME31_DROME) | [59.9](http://gemini.scripps.edu/cgi-bin/SeqCov?/wfs/bfd/3/ian/fly/DrosMelano7227.FASTAC.txt&ME31_DROME&17+19^57+40^109+43^212+16^231+16^255+26^283+25^348+27^389+16^413+47&17+19^57+10^77+20^125+27^212+16^231+16^269+12^293+15^358+17^389+16^424+36&17+19^57+10^81+16^231+16^389+16^424+36&389+16*) | (P23128) Putative ATP-dependent RNA helicase me31b (Maternal expression at 31B) |
| Q7PL68 | [35.4](http://molbio.princeton.edu/exchange/Attach/dorsal-x.html#Q7PL68) | [41.6](http://molbio.princeton.edu/exchange/Attach/orb-x.html#Q7PL68) | [35.4](http://molbio.princeton.edu/exchange/Attach/rin-x.html#Q7PL68) | [47.2](http://gemini.scripps.edu/cgi-bin/SeqCov?/wfs/bfd/3/ian/fly/DrosMelano7227.FASTAC.txt&Q7PL68&59+10^74+34^113+40&59+10^74+34^113+31&86+22^113+31&86+22&86+22*) | (Q7PL68) CG17489-PD.3 |
| Q7PL86  RL15_DROME | [14.2](http://molbio.princeton.edu/exchange/Attach/dorsal-x.html#Q7PL86) | [14.2](http://molbio.princeton.edu/exchange/Attach/orb-x.html#Q7PL86) | [14.2](http://molbio.princeton.edu/exchange/Attach/rin-x.html#Q7PL86) | [14.2](http://gemini.scripps.edu/cgi-bin/SeqCov?/wfs/bfd/3/ian/fly/DrosMelano7227.FASTAC.txt&Q7PL86&115+29&115+29&115+29&129+15*)  [14.2](http://gemini.scripps.edu/cgi-bin/SeqCov?/wfs/bfd/3/ian/fly/DrosMelano7227.FASTAC.txt&RL15_DROME&115+29&115+29&115+29&129+15*) | (Q7PL86) CG17420-PA  (O17445) 60S ribosomal protein L15 |
| Q8IMB0 | [17.9](http://molbio.princeton.edu/exchange/Attach/dorsal-x.html#Q8IMB0) | [29.4](http://molbio.princeton.edu/exchange/Attach/orb-x.html#Q8IMB0) | [30.3](http://molbio.princeton.edu/exchange/Attach/rin-x.html#Q8IMB0) | [41.7](http://gemini.scripps.edu/cgi-bin/SeqCov?/wfs/bfd/3/ian/fly/DrosMelano7227.FASTAC.txt&Q8IMB0&16+11^48+17^69+13^121+15^141+8^192+27&16+11^48+15^69+13^121+15&16+11^69+13^121+15&69+13&70+12*) | (Q8IMB0) CG2168-PB |
| Q8IMK2  Q9VAF1  Q8MLY8 | [16.8](http://molbio.princeton.edu/exchange/Attach/dorsal-x.html#Q8IMK2) | [14.5](http://molbio.princeton.edu/exchange/Attach/orb-x.html#Q8IMK2) | [30.7](http://molbio.princeton.edu/exchange/Attach/rin-x.html#Q8IMK2) | [38.0](http://gemini.scripps.edu/cgi-bin/SeqCov?/wfs/bfd/3/ian/fly/DrosMelano7227.FASTAC.txt&Q8IMK2&60+15^78+15^99+25^160+13&60+15^78+15^160+13*)  [32.5](http://gemini.scripps.edu/cgi-bin/SeqCov?/wfs/bfd/3/ian/fly/DrosMelano7227.FASTAC.txt&Q9VAF1&61+15^79+15^100+25^161+13&61+15^79+15^161+13*)  [32.7](http://gemini.scripps.edu/cgi-bin/SeqCov?/wfs/bfd/3/ian/fly/DrosMelano7227.FASTAC.txt&Q8MLY8&60+15^78+15^99+25^160+13&60+15^78+15^160+13*) | (Q8IMK2) CG7808-PD  (Q9VAF1) CG7808-PB (Fragment)  (Q8MLY8) RH06886p (SD17528p) (CG7808-PC) |
| Q8T3U2 | [21.7](http://molbio.princeton.edu/exchange/Attach/dorsal-x.html#Q8T3U2) | [21.7](http://molbio.princeton.edu/exchange/Attach/orb-x.html#Q8T3U2) | [21.7](http://molbio.princeton.edu/exchange/Attach/rin-x.html#Q8T3U2) | [21.7](http://gemini.scripps.edu/cgi-bin/SeqCov?/wfs/bfd/3/ian/fly/DrosMelano7227.FASTAC.txt&Q8T3U2&88+20^125+11&88+20^125+11&88+20^125+11*) | (Q8T3U2) GM14585p (CG8415 protein) |
| Q9I7D3 | [3.6](http://molbio.princeton.edu/exchange/Attach/dorsal-x.html#Q9I7D3) | [57.6](http://molbio.princeton.edu/exchange/Attach/orb-x.html#Q9I7D3) | [36.4](http://molbio.princeton.edu/exchange/Attach/rin-x.html#Q9I7D3) | [59.3](http://gemini.scripps.edu/cgi-bin/SeqCov?/wfs/bfd/3/ian/fly/DrosMelano7227.FASTAC.txt&Q9I7D3&2+31^66+57^135+31^170+7^201+20^232+59^293+14^364+123^495+37^579+153^746+22^931+16&2+31^66+54^142+24^201+20^232+59^293+14^364+69^451+36^495+25^522+10^581+151^746+22&14+19^66+25^93+27^154+12^203+18^233+11^252+28^282+9^295+12^364+21^396+34^451+36^496+24^522+10^586+28^616+57^684+48^746+22&93+18^154+12^203+18^268+12^364+21^396+20^451+36^507+13^590+24^651+22^685+47&100+11^203+18^268+12^396+20^453+34^508+12^594+20^652+21^685+47*) | (Q9I7D3) CG18811 protein |
| Q9V3B2 | [17.2](http://molbio.princeton.edu/exchange/Attach/dorsal-x.html#Q9V3B2) | [23.6](http://molbio.princeton.edu/exchange/Attach/orb-x.html#Q9V3B2) | [9.9](http://molbio.princeton.edu/exchange/Attach/rin-x.html#Q9V3B2) | [34.7](http://gemini.scripps.edu/cgi-bin/SeqCov?/wfs/bfd/3/ian/fly/DrosMelano7227.FASTAC.txt&Q9V3B2&207+31^253+10^291+47^345+31^381+37^440+19&291+14^345+31^440+19&345+31*) | (Q9V3B2) GBP protein |
| Q9V3C3 | [14.1](http://molbio.princeton.edu/exchange/Attach/dorsal-x.html#Q9V3C3) | [26.5](http://molbio.princeton.edu/exchange/Attach/orb-x.html#Q9V3C3) | [14.6](http://molbio.princeton.edu/exchange/Attach/rin-x.html#Q9V3C3) | [39.1](http://gemini.scripps.edu/cgi-bin/SeqCov?/wfs/bfd/3/ian/fly/DrosMelano7227.FASTAC.txt&Q9V3C3&23+20^113+40^171+37^254+21^335+21^424+27^463+21^485+15^527+14^565+17^597+15^613+36^675+16^752+13&23+20^113+23^189+19^527+14^675+16&113+21^675+16&113+21*) | (Q9V3C3) TER94 protein |
| Q9V6C4 | [7.9](http://molbio.princeton.edu/exchange/Attach/dorsal-x.html#Q9V6C4) | [3.7](http://molbio.princeton.edu/exchange/Attach/orb-x.html#Q9V6C4) | [8.4](http://molbio.princeton.edu/exchange/Attach/rin-x.html#Q9V6C4) | [15.6](http://gemini.scripps.edu/cgi-bin/SeqCov?/wfs/bfd/3/ian/fly/DrosMelano7227.FASTAC.txt&Q9V6C4&251+14^324+36^483+15^545+12^566+19^590+15^655+28&483+15^545+12&545+12*) | (Q9V6C4) CG8545 protein |
| Q9V8V3 | [22.1](http://molbio.princeton.edu/exchange/Attach/dorsal-x.html#Q9V8V3) | [49.8](http://molbio.princeton.edu/exchange/Attach/orb-x.html#Q9V8V3) | [20.8](http://molbio.princeton.edu/exchange/Attach/rin-x.html#Q9V8V3) | [53.1](http://gemini.scripps.edu/cgi-bin/SeqCov?/wfs/bfd/3/ian/fly/DrosMelano7227.FASTAC.txt&Q9V8V3&29+39^72+59^132+32^172+12^226+25^262+24^292+15^319+9^372+17^390+10&29+39^93+38^132+32^272+14^319+9^372+17^390+10&29+27^132+32^372+17^390+10&132+32^377+12&143+21*) | (Q9V8V3) Beta tubulin 56D protein |
| Q9VAX7 | [5.2](http://molbio.princeton.edu/exchange/Attach/dorsal-x.html#Q9VAX7) | [14.8](http://molbio.princeton.edu/exchange/Attach/orb-x.html#Q9VAX7) | [5.2](http://molbio.princeton.edu/exchange/Attach/rin-x.html#Q9VAX7) | [14.8](http://gemini.scripps.edu/cgi-bin/SeqCov?/wfs/bfd/3/ian/fly/DrosMelano7227.FASTAC.txt&Q9VAX7&78+26^318+24^428+17^446+10&428+17^446+10&428+17^446+10&433+12*) | (Q9VAX7) CG4869 protein |
| Q9VEN7 | [5.5](http://molbio.princeton.edu/exchange/Attach/dorsal-x.html#Q9VEN7) | [4.5](http://molbio.princeton.edu/exchange/Attach/orb-x.html#Q9VEN7) | [7.2](http://molbio.princeton.edu/exchange/Attach/rin-x.html#Q9VEN7) | [14.6](http://gemini.scripps.edu/cgi-bin/SeqCov?/wfs/bfd/3/ian/fly/DrosMelano7227.FASTAC.txt&Q9VEN7&147+15^316+21^346+11^361+21^514+17&516+15*) | (Q9VEN7) CG3983-PB (CG3983-PA) |
| Q9VFE4 | [14.3](http://molbio.princeton.edu/exchange/Attach/dorsal-x.html#Q9VFE4) | [12.6](http://molbio.princeton.edu/exchange/Attach/orb-x.html#Q9VFE4) | [14.3](http://molbio.princeton.edu/exchange/Attach/rin-x.html#Q9VFE4) | [21.3](http://gemini.scripps.edu/cgi-bin/SeqCov?/wfs/bfd/3/ian/fly/DrosMelano7227.FASTAC.txt&Q9VFE4&49+20^172+14^194+15&49+20^173+13&173+13&173+13*) | (Q9VFE4) CG7014 protein (RE17836p) |
| Q9VJ19 | [27.9](http://molbio.princeton.edu/exchange/Attach/dorsal-x.html#Q9VJ19) | [43.2](http://molbio.princeton.edu/exchange/Attach/orb-x.html#Q9VJ19) | [27.9](http://molbio.princeton.edu/exchange/Attach/rin-x.html#Q9VJ19) | [43.2](http://gemini.scripps.edu/cgi-bin/SeqCov?/wfs/bfd/3/ian/fly/DrosMelano7227.FASTAC.txt&Q9VJ19&45+13^69+19^91+16&45+12^69+19&45+12^69+19&45+12*) | (Q9VJ19) CG10652 protein (RE25263p) |
| Q9VN21 | [8.8](http://molbio.princeton.edu/exchange/Attach/dorsal-x.html#Q9VN21) | [10.8](http://molbio.princeton.edu/exchange/Attach/orb-x.html#Q9VN21) | [6.2](http://molbio.princeton.edu/exchange/Attach/rin-x.html#Q9VN21) | [17.1](http://gemini.scripps.edu/cgi-bin/SeqCov?/wfs/bfd/3/ian/fly/DrosMelano7227.FASTAC.txt&Q9VN21&70+13^222+16^445+27^500+37&445+27^516+21*) | (Q9VN21) CG14648 protein (LD30155P) |
| Q9VS34 | [25.0](http://molbio.princeton.edu/exchange/Attach/dorsal-x.html#Q9VS34) | [25.0](http://molbio.princeton.edu/exchange/Attach/orb-x.html#Q9VS34) | [25.0](http://molbio.princeton.edu/exchange/Attach/rin-x.html#Q9VS34) | [25.0](http://gemini.scripps.edu/cgi-bin/SeqCov?/wfs/bfd/3/ian/fly/DrosMelano7227.FASTAC.txt&Q9VS34&72+20^98+13^116+14&72+20^98+13^116+14&72+20^98+13^116+14*) | (Q9VS34) CG8615 protein (Ribosomal protein L18) (RH01814p) |
| Q9VV75 | [10.0](http://molbio.princeton.edu/exchange/Attach/dorsal-x.html#Q9VV75) | [9.3](http://molbio.princeton.edu/exchange/Attach/orb-x.html#Q9VV75) | [8.6](http://molbio.princeton.edu/exchange/Attach/rin-x.html#Q9VV75) | [20.7](http://gemini.scripps.edu/cgi-bin/SeqCov?/wfs/bfd/3/ian/fly/DrosMelano7227.FASTAC.txt&Q9VV75&209+28^256+16^292+22^391+25&256+16&256+16&261+11*) | (Q9VV75) CG4169 protein (AT02348p) |
| Q9VYU3 | [10.4](http://molbio.princeton.edu/exchange/Attach/dorsal-x.html#Q9VYU3) | [32.8](http://molbio.princeton.edu/exchange/Attach/orb-x.html#Q9VYU3) | [14.5](http://molbio.princeton.edu/exchange/Attach/rin-x.html#Q9VYU3) | [39.9](http://gemini.scripps.edu/cgi-bin/SeqCov?/wfs/bfd/3/ian/fly/DrosMelano7227.FASTAC.txt&Q9VYU3&27+21^62+14^103+12^124+29^164+18^186+28^307+18^327+10^354+14^448+27^563+11^594+25^622+35&27+21^62+14^127+12^165+17^198+16^307+18^355+13^563+11^622+12&165+17^198+16^307+18^355+13&202+12&206+8*) | (Q9VYU3) Hsc70-3 protein |
| Q9W1B9 | [27.9](http://molbio.princeton.edu/exchange/Attach/dorsal-x.html#Q9W1B9) | [39.4](http://molbio.princeton.edu/exchange/Attach/orb-x.html#Q9W1B9) | [39.4](http://molbio.princeton.edu/exchange/Attach/rin-x.html#Q9W1B9) | [39.4](http://gemini.scripps.edu/cgi-bin/SeqCov?/wfs/bfd/3/ian/fly/DrosMelano7227.FASTAC.txt&Q9W1B9&17+15^100+15^131+35&17+15^100+15^131+35&17+15^100+15^131+16&100+15&100+15*) | (Q9W1B9) RE28824p (CG3195-PA) (CG3195-PB) |
| Q9W1V3 | [11.3](http://molbio.princeton.edu/exchange/Attach/dorsal-x.html#Q9W1V3) | [18.6](http://molbio.princeton.edu/exchange/Attach/orb-x.html#Q9W1V3) | [15.1](http://molbio.princeton.edu/exchange/Attach/rin-x.html#Q9W1V3) | [34.0](http://gemini.scripps.edu/cgi-bin/SeqCov?/wfs/bfd/3/ian/fly/DrosMelano7227.FASTAC.txt&Q9W1V3&169+52^250+31^291+19^330+15&270+11^291+19^330+14&291+19*) | (Q9W1V3) FIB protein |
| Q9W5R8 | [28.4](http://molbio.princeton.edu/exchange/Attach/dorsal-x.html#Q9W5R8) | [46.8](http://molbio.princeton.edu/exchange/Attach/orb-x.html#Q9W5R8) | [21.1](http://molbio.princeton.edu/exchange/Attach/rin-x.html#Q9W5R8) | [50.2](http://gemini.scripps.edu/cgi-bin/SeqCov?/wfs/bfd/3/ian/fly/DrosMelano7227.FASTAC.txt&Q9W5R8&59+10^74+34^113+40^159+20^197+25^233+10^282+11&59+10^74+34^113+31^165+14^197+24^233+10^282+11&86+22^113+31^165+14^200+21^233+9&86+22^210+11&86+22*) | (Q9W5R8) RE33114p (CG17489-PA.3) |
| RL3_DROME | [9.9](http://molbio.princeton.edu/exchange/Attach/dorsal-x.html#RL3_DROME) | [24.1](http://molbio.princeton.edu/exchange/Attach/orb-x.html#RL3_DROME) | [24.6](http://molbio.princeton.edu/exchange/Attach/rin-x.html#RL3_DROME) | [35.2](http://gemini.scripps.edu/cgi-bin/SeqCov?/wfs/bfd/3/ian/fly/DrosMelano7227.FASTAC.txt&RL3_DROME&39+11^70+45^177+16^203+21^250+11^312+22^396+20&39+11^100+15^203+21^250+11^396+20&39+11^203+21^396+20&203+21^396+20&396+20*) | (O16797) 60S ribosomal protein L3 |
| RL4_DROME | [10.1](http://molbio.princeton.edu/exchange/Attach/dorsal-x.html#RL4_DROME) | [16.7](http://molbio.princeton.edu/exchange/Attach/orb-x.html#RL4_DROME) | [12.8](http://molbio.princeton.edu/exchange/Attach/rin-x.html#RL4_DROME) | [26.8](http://gemini.scripps.edu/cgi-bin/SeqCov?/wfs/bfd/3/ian/fly/DrosMelano7227.FASTAC.txt&RL4_DROME&33+16^53+22^125+39^226+12^252+20&33+16^53+22^125+39^252+20&53+22*) | (P09180) 60S ribosomal protein L4 (L1) |
| RL9_DROME | [44.2](http://molbio.princeton.edu/exchange/Attach/dorsal-x.html#RL9_DROME) | [68.9](http://molbio.princeton.edu/exchange/Attach/orb-x.html#RL9_DROME) | [45.3](http://molbio.princeton.edu/exchange/Attach/rin-x.html#RL9_DROME) | [83.2](http://gemini.scripps.edu/cgi-bin/SeqCov?/wfs/bfd/3/ian/fly/DrosMelano7227.FASTAC.txt&RL9_DROME&3+15^24+66^92+22^123+44^172+11&37+26^70+20^92+22^123+44^173+10&40+12^70+20^92+22^123+44^173+10&40+12^70+20^92+22^123+44^173+10&40+11^92+22^124+43*) | (P50882) 60S ribosomal protein L9 |
| RLA0_DROME | [54.6](http://molbio.princeton.edu/exchange/Attach/dorsal-x.html#RLA0_DROME) | [67.8](http://molbio.princeton.edu/exchange/Attach/orb-x.html#RLA0_DROME) | [58.7](http://molbio.princeton.edu/exchange/Attach/rin-x.html#RLA0_DROME) | [74.1](http://gemini.scripps.edu/cgi-bin/SeqCov?/wfs/bfd/3/ian/fly/DrosMelano7227.FASTAC.txt&RLA0_DROME&11+28^45+33^84+9^113+100^215+46^275+19&17+22^49+14^67+11^84+9^113+34^150+63^215+46^275+19&17+22^49+9^67+11^114+33^150+63^215+46^275+19&17+22^49+9^67+11^120+27^150+17^184+29^215+25^249+12^275+19&27+12^49+9^122+13^150+17^188+25^215+25^250+11^280+14*) | (P19889) 60S acidic ribosomal protein P0 (DNA-(apurinic or apyrimidinic site) lyase) (EC 4.2.99.18) (Apurinic-apyrimidinic endonuclease) |
| RLA1_DROME | [70.5](http://molbio.princeton.edu/exchange/Attach/dorsal-x.html#RLA1_DROME) | [70.5](http://molbio.princeton.edu/exchange/Attach/orb-x.html#RLA1_DROME) | [63.4](http://molbio.princeton.edu/exchange/Attach/rin-x.html#RLA1_DROME) | [70.5](http://gemini.scripps.edu/cgi-bin/SeqCov?/wfs/bfd/3/ian/fly/DrosMelano7227.FASTAC.txt&RLA1_DROME&5+23^34+56&5+23^34+56&5+23^34+16^58+32&5+23^39+11^58+32&5+23^40+10^58+32*) | (P08570) 60S acidic ribosomal protein P1 (RP21C) (Acidic ribosomal protein RPA2) |
| RLA2_DROME | [28.3](http://molbio.princeton.edu/exchange/Attach/dorsal-x.html#RLA2_DROME) | [23.0](http://molbio.princeton.edu/exchange/Attach/orb-x.html#RLA2_DROME) | [28.3](http://molbio.princeton.edu/exchange/Attach/rin-x.html#RLA2_DROME) | [28.3](http://gemini.scripps.edu/cgi-bin/SeqCov?/wfs/bfd/3/ian/fly/DrosMelano7227.FASTAC.txt&RLA2_DROME&3+13^20+19&3+13^20+19&3+13^26+13&3+13^26+13&3+13^26+13*) | (P05389) 60S acidic ribosomal protein P2 (Acidic ribosomal protein RPA1) |
| RS3A_DROME | [14.6](http://molbio.princeton.edu/exchange/Attach/dorsal-x.html#RS3A_DROME) | [24.0](http://molbio.princeton.edu/exchange/Attach/orb-x.html#RS3A_DROME) | [14.6](http://molbio.princeton.edu/exchange/Attach/rin-x.html#RS3A_DROME) | [24.0](http://gemini.scripps.edu/cgi-bin/SeqCov?/wfs/bfd/3/ian/fly/DrosMelano7227.FASTAC.txt&RS3A_DROME&65+11^97+17^118+13^170+15^190+8&65+11^97+15^118+13^170+15&65+11^118+13^170+15&118+13&119+12*) | (P55830) 40S ribosomal protein S3a (C3 protein) |
| RS4_DROME | [14.2](http://molbio.princeton.edu/exchange/Attach/dorsal-x.html#RS4_DROME) | [29.1](http://molbio.princeton.edu/exchange/Attach/orb-x.html#RS4_DROME) | [16.1](http://molbio.princeton.edu/exchange/Attach/rin-x.html#RS4_DROME) | [35.6](http://gemini.scripps.edu/cgi-bin/SeqCov?/wfs/bfd/3/ian/fly/DrosMelano7227.FASTAC.txt&RS4_DROME&78+17^135+11^149+20^175+13^199+13^222+19&137+9^149+20^175+13^199+13^222+19&149+20^201+11^222+9&149+20^201+11^222+9*) | (P41042) 40S ribosomal protein S4 |
| RS5_DROME | [14.5](http://molbio.princeton.edu/exchange/Attach/dorsal-x.html#RS5_DROME) | [6.1](http://molbio.princeton.edu/exchange/Attach/orb-x.html#RS5_DROME) | [14.5](http://molbio.princeton.edu/exchange/Attach/rin-x.html#RS5_DROME) | [14.9](http://gemini.scripps.edu/cgi-bin/SeqCov?/wfs/bfd/3/ian/fly/DrosMelano7227.FASTAC.txt&RS5_DROME&47+20^170+14&47+20^171+13&171+13&171+13*) | (Q24186) 40S ribosomal protein S5 |
| TBA1_DROME  TBA3_DROME | [19.3](http://molbio.princeton.edu/exchange/Attach/dorsal-x.html#TBA1_DROME) | [22.9](http://molbio.princeton.edu/exchange/Attach/orb-x.html#TBA1_DROME) | [17.6](http://molbio.princeton.edu/exchange/Attach/rin-x.html#TBA1_DROME) | [39.6](http://gemini.scripps.edu/cgi-bin/SeqCov?/wfs/bfd/3/ian/fly/DrosMelano7227.FASTAC.txt&TBA1_DROME&41+20^65+15^85+12^113+9^230+75^327+10^340+13^374+17^395+7&41+20^65+15^230+51&244+21*)  [39.6](http://gemini.scripps.edu/cgi-bin/SeqCov?/wfs/bfd/3/ian/fly/DrosMelano7227.FASTAC.txt&TBA3_DROME&41+20^65+15^85+12^113+9^230+75^327+10^340+13^374+17^395+7&41+20^65+15^230+51&244+21*) | (P06603) Tubulin alpha-1 chain  (P06605) Tubulin alpha-3 chain |
| TBA2_DROME | [14.9](http://molbio.princeton.edu/exchange/Attach/dorsal-x.html#TBA2_DROME) | [14.5](http://molbio.princeton.edu/exchange/Attach/orb-x.html#TBA2_DROME) | [7.8](http://molbio.princeton.edu/exchange/Attach/rin-x.html#TBA2_DROME) | [21.4](http://gemini.scripps.edu/cgi-bin/SeqCov?/wfs/bfd/3/ian/fly/DrosMelano7227.FASTAC.txt&TBA2_DROME&65+15^85+12^230+35^327+10^374+17^395+7&65+15^230+35&244+21*) | (P06604) Tubulin alpha-2 chain |
| TBB1_DROME | [22.6](http://molbio.princeton.edu/exchange/Attach/dorsal-x.html#TBB1_DROME) | [54.6](http://molbio.princeton.edu/exchange/Attach/orb-x.html#TBB1_DROME) | [21.3](http://molbio.princeton.edu/exchange/Attach/rin-x.html#TBB1_DROME) | [57.9](http://gemini.scripps.edu/cgi-bin/SeqCov?/wfs/bfd/3/ian/fly/DrosMelano7227.FASTAC.txt&TBB1_DROME&3+56^63+59^123+32^163+12^217+25^253+24^283+15^310+9^363+17^381+10&20+39^84+38^123+32^263+14^310+9^363+17^381+10&20+27^123+32^363+17^381+10&123+32^368+12&134+21*) | (Q24560) Tubulin beta-1 chain |
| TBB2_DROME | [7.6](http://molbio.princeton.edu/exchange/Attach/dorsal-x.html#TBB2_DROME) | [27.8](http://molbio.princeton.edu/exchange/Attach/orb-x.html#TBB2_DROME) | [9.6](http://molbio.princeton.edu/exchange/Attach/rin-x.html#TBB2_DROME) | [27.8](http://gemini.scripps.edu/cgi-bin/SeqCov?/wfs/bfd/3/ian/fly/DrosMelano7227.FASTAC.txt&TBB2_DROME&84+38^134+21^253+24^283+15^310+9^363+17&104+18^138+17^263+14^310+9^363+17&138+17^363+17&368+12*) | (P08840) Tubulin beta-2 chain |
| TBG2_DROME | [12.0](http://molbio.princeton.edu/exchange/Attach/dorsal-x.html#TBG2_DROME) | [7.7](http://molbio.princeton.edu/exchange/Attach/orb-x.html#TBG2_DROME) | [19.0](http://molbio.princeton.edu/exchange/Attach/rin-x.html#TBG2_DROME) | [26.7](http://gemini.scripps.edu/cgi-bin/SeqCov?/wfs/bfd/3/ian/fly/DrosMelano7227.FASTAC.txt&TBG2_DROME&85+29^125+32^198+15^374+17^427+29&85+11^198+15^427+29*) | (P42271) Tubulin gamma-2 chain (Gamma-2 tubulin) |
| VIT1_DROME | [18.2](http://molbio.princeton.edu/exchange/Attach/dorsal-x.html#VIT1_DROME) | [50.8](http://molbio.princeton.edu/exchange/Attach/orb-x.html#VIT1_DROME) | [26.7](http://molbio.princeton.edu/exchange/Attach/rin-x.html#VIT1_DROME) | [50.8](http://gemini.scripps.edu/cgi-bin/SeqCov?/wfs/bfd/3/ian/fly/DrosMelano7227.FASTAC.txt&VIT1_DROME&25+33^66+38^119+23^199+14^219+8^234+37^301+48^386+22&35+23^66+38^119+23^199+14^234+37^301+21^386+22&66+38^119+23^199+14^245+26^301+21^386+22&75+24^119+23^201+12^260+11^301+21&76+11^119+23*) | (P02843) Vitellogenin I precursor (Yolk protein 1) |
| VIT3_DROME | [16.9](http://molbio.princeton.edu/exchange/Attach/dorsal-x.html#VIT3_DROME) | [46.0](http://molbio.princeton.edu/exchange/Attach/orb-x.html#VIT3_DROME) | [16.9](http://molbio.princeton.edu/exchange/Attach/rin-x.html#VIT3_DROME) | [48.8](http://gemini.scripps.edu/cgi-bin/SeqCov?/wfs/bfd/3/ian/fly/DrosMelano7227.FASTAC.txt&VIT3_DROME&33+20^76+20^114+19^144+16^190+68^281+31^327+12^368+7^376+12&33+20^76+20^114+19^190+19^210+48^291+21^376+12&84+12^114+19^190+19^210+40^291+21&190+19^210+23^235+15^291+14*) | (P06607) Vitellogenin III precursor (Yolk protein 3) |
| **55** | **X** | **X** | **X** |  |  |

| ***Locus*** | ***dorsal*** | ***orb*** | ***rin*** | ***Total*** | ***Description*** |
| --- | --- | --- | --- | --- | --- |
| AAF54559 | [1.4](http://molbio.princeton.edu/exchange/Attach/dorsal-x.html#AAF54559) |  | [1.4](http://molbio.princeton.edu/exchange/Attach/rin-x.html#AAF54559) | [1.4](http://gemini.scripps.edu/cgi-bin/SeqCov?/wfs/bfd/3/ian/fly/DrosMelano7227.FASTAC.txt&AAF54559&685+20^1123+20&685+20^1123+20*) | (AAF54559) CG14692-PA |
| ENO_DROME  Q9VQ38 | [12.9](http://molbio.princeton.edu/exchange/Attach/dorsal-x.html#ENO_DROME) |  | [9.2](http://molbio.princeton.edu/exchange/Attach/rin-x.html#ENO_DROME) | [17.1](http://gemini.scripps.edu/cgi-bin/SeqCov?/wfs/bfd/3/ian/fly/DrosMelano7227.FASTAC.txt&ENO_DROME&16+35^80+17^374+22&374+22*)  [17.1](http://gemini.scripps.edu/cgi-bin/SeqCov?/wfs/bfd/3/ian/fly/DrosMelano7227.FASTAC.txt&Q9VQ38&16+35^80+17^374+22&374+22*) | (P15007) Enolase (EC 4.2.1.11) (2-phosphoglycerate dehydratase) (2-phospho-D-glycerate hydro-lyase)  (Q9VQ38) CG17654 protein (EC 4.2.1.11) (Enolase) (2-phosphoglycerate dehydratase) (2-phospho-D-glycerate hydro-lyase) |
| IF6_DROME | [15.5](http://molbio.princeton.edu/exchange/Attach/dorsal-x.html#IF6_DROME) |  | [15.9](http://molbio.princeton.edu/exchange/Attach/rin-x.html#IF6_DROME) | [25.7](http://gemini.scripps.edu/cgi-bin/SeqCov?/wfs/bfd/3/ian/fly/DrosMelano7227.FASTAC.txt&IF6_DROME&5+14^140+49&5+14*) | (P56538) Probable eukaryotic translation initiation factor 6 (eIF-6) |
| VTU1_DROME | [25.5](http://molbio.princeton.edu/exchange/Attach/dorsal-x.html#VTU1_DROME) |  | [25.5](http://molbio.princeton.edu/exchange/Attach/rin-x.html#VTU1_DROME) | [25.5](http://gemini.scripps.edu/cgi-bin/SeqCov?/wfs/bfd/3/ian/fly/DrosMelano7227.FASTAC.txt&VTU1_DROME&19+36&19+36&21+34&21+34*) | (P11449) Vitelline membrane protein Vm26Aa precursor (Protein TU-2) (Protein SV17.5) |
| **4** | **X** |  | **X** |  |  |

| ***Locus*** | ***dorsal*** | ***orb*** | ***rin*** | ***Total*** | ***Description*** |
| --- | --- | --- | --- | --- | --- |
| AAF49317 | [7.6](http://molbio.princeton.edu/exchange/Attach/dorsal-x.html#AAF49317) | [10.4](http://molbio.princeton.edu/exchange/Attach/orb-x.html#AAF49317) |  | [12.9](http://gemini.scripps.edu/cgi-bin/SeqCov?/wfs/bfd/3/ian/fly/DrosMelano7227.FASTAC.txt&AAF49317&10+13^145+14^283+13^451+25&10+13^451+25*) | (AAF49317) CG6143-PA |
| AAF59403 | [3.5](http://molbio.princeton.edu/exchange/Attach/dorsal-x.html#AAF59403) | [5.4](http://molbio.princeton.edu/exchange/Attach/orb-x.html#AAF59403) |  | [8.9](http://gemini.scripps.edu/cgi-bin/SeqCov?/wfs/bfd/3/ian/fly/DrosMelano7227.FASTAC.txt&AAF59403&28+24^244+21^278+20^344+12^699+26^938+17*) | (AAF59403) CG10811-PA |
| AAM68717 | [14.8](http://molbio.princeton.edu/exchange/Attach/dorsal-x.html#AAM68717) | [15.5](http://molbio.princeton.edu/exchange/Attach/orb-x.html#AAM68717) |  | [30.3](http://gemini.scripps.edu/cgi-bin/SeqCov?/wfs/bfd/3/ian/fly/DrosMelano7227.FASTAC.txt&AAM68717&75+42^169+38^302+20*) | (AAM68717) CG8996-PA (CG8996-PB) |
| AAN09050  RSP4_DROME | [16.6](http://molbio.princeton.edu/exchange/Attach/dorsal-x.html#AAN09050) | [28.1](http://molbio.princeton.edu/exchange/Attach/orb-x.html#AAN09050) |  | [28.1](http://gemini.scripps.edu/cgi-bin/SeqCov?/wfs/bfd/3/ian/fly/DrosMelano7227.FASTAC.txt&AAN09050&107+17^164+35^269+36&107+17^164+35*)  [32.6](http://gemini.scripps.edu/cgi-bin/SeqCov?/wfs/bfd/3/ian/fly/DrosMelano7227.FASTAC.txt&RSP4_DROME&64+17^121+35^226+36&64+17^121+35*) | (AAN09050) CG14792-PD  (P38979) 40S ribosomal protein SA (p40) (Stubarista protein) (Laminin receptor homolog) (K14) |
| AAN14138  Q8IMM4 | [2.8](http://molbio.princeton.edu/exchange/Attach/dorsal-x.html#AAN14138) | [6.8](http://molbio.princeton.edu/exchange/Attach/orb-x.html#AAN14138) |  | [7.9](http://gemini.scripps.edu/cgi-bin/SeqCov?/wfs/bfd/3/ian/fly/DrosMelano7227.FASTAC.txt&AAN14138&134+22^238+23^267+10^503+16^1029+40&238+23*)  [7.9](http://gemini.scripps.edu/cgi-bin/SeqCov?/wfs/bfd/3/ian/fly/DrosMelano7227.FASTAC.txt&Q8IMM4&134+22^238+23^267+10^503+16^1029+40&238+23*) | (AAN14138) CG14066-PC (Fragment)  (Q8IMM4) CG14066-PC |
| AAS64975  PEP_DROME | [5.5](http://molbio.princeton.edu/exchange/Attach/dorsal-x.html#AAS64975) | [10.8](http://molbio.princeton.edu/exchange/Attach/orb-x.html#AAS64975) |  | [12.7](http://gemini.scripps.edu/cgi-bin/SeqCov?/wfs/bfd/3/ian/fly/DrosMelano7227.FASTAC.txt&AAS64975&53+23^201+13^336+14^474+13^642+25&201+13^642+25*)  [12.3](http://gemini.scripps.edu/cgi-bin/SeqCov?/wfs/bfd/3/ian/fly/DrosMelano7227.FASTAC.txt&PEP_DROME&76+23^224+13^359+14^497+13^665+25&224+13^665+25*) | (AAS64975) CG6143-PC  (P41073) Zinc finger protein on ecdysone puffs |
| EF2_DROME | [4.0](http://molbio.princeton.edu/exchange/Attach/dorsal-x.html#EF2_DROME) | [8.2](http://molbio.princeton.edu/exchange/Attach/orb-x.html#EF2_DROME) |  | [12.2](http://gemini.scripps.edu/cgi-bin/SeqCov?/wfs/bfd/3/ian/fly/DrosMelano7227.FASTAC.txt&EF2_DROME&166+18^339+13^442+25^545+13^753+34*) | (P13060) Elongation factor 2 (EF-2) |
| HS83_DROME | [7.9](http://molbio.princeton.edu/exchange/Attach/dorsal-x.html#HS83_DROME) | [14.2](http://molbio.princeton.edu/exchange/Attach/orb-x.html#HS83_DROME) |  | [22.2](http://gemini.scripps.edu/cgi-bin/SeqCov?/wfs/bfd/3/ian/fly/DrosMelano7227.FASTAC.txt&HS83_DROME&73+28^142+20^198+10^285+28^353+33^450+19^577+21*) | (P02828) Heat shock protein 83 (HSP 82) |
| MYSN_DROME | [2.7](http://molbio.princeton.edu/exchange/Attach/dorsal-x.html#MYSN_DROME) | [4.4](http://molbio.princeton.edu/exchange/Attach/orb-x.html#MYSN_DROME) |  | [7.1](http://gemini.scripps.edu/cgi-bin/SeqCov?/wfs/bfd/3/ian/fly/DrosMelano7227.FASTAC.txt&MYSN_DROME&252+33^300+21^433+14^1353+21^1469+16^1590+17^1782+21*) | (Q99323) Myosin heavy chain, non-muscle (Zipper protein) (Myosin II) |
| NO60_DROME | [5.5](http://molbio.princeton.edu/exchange/Attach/dorsal-x.html#NO60_DROME) | [12.4](http://molbio.princeton.edu/exchange/Attach/orb-x.html#NO60_DROME) |  | [17.9](http://gemini.scripps.edu/cgi-bin/SeqCov?/wfs/bfd/3/ian/fly/DrosMelano7227.FASTAC.txt&NO60_DROME&42+14^87+22^176+14^210+16^283+13^409+12&95+14^283+13&95+14*) | (O44081) Nucleolar protein AT band 60B (Minifly protein) |
| NOG1_DROME | [6.3](http://molbio.princeton.edu/exchange/Attach/dorsal-x.html#NOG1_DROME) | [2.1](http://molbio.princeton.edu/exchange/Attach/orb-x.html#NOG1_DROME) |  | [8.4](http://gemini.scripps.edu/cgi-bin/SeqCov?/wfs/bfd/3/ian/fly/DrosMelano7227.FASTAC.txt&NOG1_DROME&277+14^315+19^527+22&277+14*) | (Q9V411) Probable nucleolar GTP-binding protein 1 |
| O61380 | [2.8](http://molbio.princeton.edu/exchange/Attach/dorsal-x.html#O61380) | [7.0](http://molbio.princeton.edu/exchange/Attach/orb-x.html#O61380) |  | [9.8](http://gemini.scripps.edu/cgi-bin/SeqCov?/wfs/bfd/3/ian/fly/DrosMelano7227.FASTAC.txt&O61380&113+17^311+26^349+24^565+21^599+20^665+12^1020+26^1259+17*) | (O61380) EIF-4G protein |
| Q7PL67 | [16.1](http://molbio.princeton.edu/exchange/Attach/dorsal-x.html#Q7PL67) | [43.8](http://molbio.princeton.edu/exchange/Attach/orb-x.html#Q7PL67) |  | [43.8](http://gemini.scripps.edu/cgi-bin/SeqCov?/wfs/bfd/3/ian/fly/DrosMelano7227.FASTAC.txt&Q7PL67&3+14^35+25^71+10^120+11&3+14^35+24^71+10^120+11&38+21^71+9&48+11*) | (Q7PL67) CG17489-PC.3 |
| Q8IPM3 | [14.2](http://molbio.princeton.edu/exchange/Attach/dorsal-x.html#Q8IPM3) | [17.5](http://molbio.princeton.edu/exchange/Attach/orb-x.html#Q8IPM3) |  | [23.7](http://gemini.scripps.edu/cgi-bin/SeqCov?/wfs/bfd/3/ian/fly/DrosMelano7227.FASTAC.txt&Q8IPM3&15+16^238+27^293+37&238+27*) | (Q8IPM3) CG14648-PB |
| Q9VAX8 | [5.3](http://molbio.princeton.edu/exchange/Attach/dorsal-x.html#Q9VAX8) | [3.1](http://molbio.princeton.edu/exchange/Attach/orb-x.html#Q9VAX8) |  | [8.4](http://gemini.scripps.edu/cgi-bin/SeqCov?/wfs/bfd/3/ian/fly/DrosMelano7227.FASTAC.txt&Q9VAX8&499+13^658+19^717+17^876+16^918+17*) | (Q9VAX8) CG4849 protein (LD28793p) |
| Q9VH07 | [10.7](http://molbio.princeton.edu/exchange/Attach/dorsal-x.html#Q9VH07) | [8.3](http://molbio.princeton.edu/exchange/Attach/orb-x.html#Q9VH07) |  | [19.1](http://gemini.scripps.edu/cgi-bin/SeqCov?/wfs/bfd/3/ian/fly/DrosMelano7227.FASTAC.txt&Q9VH07&77+14^129+24^206+20^379+15^405+14*) | (Q9VH07) Putative pontin protein (LD08555p) |
| Q9W0W8 | [2.6](http://molbio.princeton.edu/exchange/Attach/dorsal-x.html#Q9W0W8) | [2.7](http://molbio.princeton.edu/exchange/Attach/orb-x.html#Q9W0W8) |  | [5.4](http://gemini.scripps.edu/cgi-bin/SeqCov?/wfs/bfd/3/ian/fly/DrosMelano7227.FASTAC.txt&Q9W0W8&339+21^472+14^1392+21^1508+16^1629+17^1821+21*) | (Q9W0W8) ZIP protein |
| RFA1_DROME | [7.3](http://molbio.princeton.edu/exchange/Attach/dorsal-x.html#RFA1_DROME) | [10.0](http://molbio.princeton.edu/exchange/Attach/orb-x.html#RFA1_DROME) |  | [17.2](http://gemini.scripps.edu/cgi-bin/SeqCov?/wfs/bfd/3/ian/fly/DrosMelano7227.FASTAC.txt&RFA1_DROME&2+12^96+17^145+34^227+15^435+14^572+12&146+33*) | (Q24492) Replication protein A 70 kDa DNA-binding subunit (RP-A) (RF-A) (Replication factor-A protein 1) (Single-stranded DNA-binding protein) (DmRPA1) |
| RL22_DROME | [14.7](http://molbio.princeton.edu/exchange/Attach/dorsal-x.html#RL22_DROME) | [19.1](http://molbio.princeton.edu/exchange/Attach/orb-x.html#RL22_DROME) |  | [26.8](http://gemini.scripps.edu/cgi-bin/SeqCov?/wfs/bfd/3/ian/fly/DrosMelano7227.FASTAC.txt&RL22_DROME&65+23^138+24^193+21^225+12&138+24^193+21&138+24&140+22&141+21*) | (P50887) 60S ribosomal protein L22 |
| RS6_DROME | [12.9](http://molbio.princeton.edu/exchange/Attach/dorsal-x.html#RS6_DROME) | [14.5](http://molbio.princeton.edu/exchange/Attach/orb-x.html#RS6_DROME) |  | [21.4](http://gemini.scripps.edu/cgi-bin/SeqCov?/wfs/bfd/3/ian/fly/DrosMelano7227.FASTAC.txt&RS6_DROME&3+21^32+15^99+17&3+21^32+15*) | (P29327) 40S ribosomal protein S6 |
| TBA4_DROME | [12.1](http://molbio.princeton.edu/exchange/Attach/dorsal-x.html#TBA4_DROME) | [11.3](http://molbio.princeton.edu/exchange/Attach/orb-x.html#TBA4_DROME) |  | [23.4](http://gemini.scripps.edu/cgi-bin/SeqCov?/wfs/bfd/3/ian/fly/DrosMelano7227.FASTAC.txt&TBA4_DROME&40+22^241+51^350+14^442+21&351+13&351+13*) | (P06606) Tubulin alpha-4 chain |
| **21** | **X** | **X** |  |  |  |

| ***Locus*** | ***dorsal*** | ***orb*** | ***rin*** | ***Total*** | ***Description*** |
| --- | --- | --- | --- | --- | --- |
| AAF46288 | [14.7](http://molbio.princeton.edu/exchange/Attach/dorsal-x.html#AAF46288) |  |  | [14.7](http://gemini.scripps.edu/cgi-bin/SeqCov?/wfs/bfd/3/ian/fly/DrosMelano7227.FASTAC.txt&AAF46288&1+15^68+17*) | (AAF46288) CG10944-PA |
| AAF46300  Q9W3M5 | [6.6](http://molbio.princeton.edu/exchange/Attach/dorsal-x.html#AAF46300) |  |  | [6.6](http://gemini.scripps.edu/cgi-bin/SeqCov?/wfs/bfd/3/ian/fly/DrosMelano7227.FASTAC.txt&AAF46300&113+38^689+12*)  [6.8](http://gemini.scripps.edu/cgi-bin/SeqCov?/wfs/bfd/3/ian/fly/DrosMelano7227.FASTAC.txt&Q9W3M5&113+38^689+12*) | (AAF46300) CG1530-PA  (Q9W3M5) CG1530 protein |
| AAF50194  AAN11936 | [9.8](http://molbio.princeton.edu/exchange/Attach/dorsal-x.html#AAF50194) |  |  | [9.8](http://gemini.scripps.edu/cgi-bin/SeqCov?/wfs/bfd/3/ian/fly/DrosMelano7227.FASTAC.txt&AAF50194&23+12^179+20^237+14^358+18^793+22&793+22*)  [9.7](http://gemini.scripps.edu/cgi-bin/SeqCov?/wfs/bfd/3/ian/fly/DrosMelano7227.FASTAC.txt&AAN11936&33+12^189+20^247+14^368+18^803+22&803+22*) | (AAF50194) CG6718-PA  (AAN11936) CG6718-PB |
| AAF50542  Q9VS85  AAN12042  AAF50543 | [8.5](http://molbio.princeton.edu/exchange/Attach/dorsal-x.html#AAF50542) |  |  | [8.5](http://gemini.scripps.edu/cgi-bin/SeqCov?/wfs/bfd/3/ian/fly/DrosMelano7227.FASTAC.txt&AAF50542&79+10^155+20^231+13^352+27*)  [8.5](http://gemini.scripps.edu/cgi-bin/SeqCov?/wfs/bfd/3/ian/fly/DrosMelano7227.FASTAC.txt&Q9VS85&79+10^155+20^231+13^352+27*)  [8.9](http://gemini.scripps.edu/cgi-bin/SeqCov?/wfs/bfd/3/ian/fly/DrosMelano7227.FASTAC.txt&AAN12042&79+10^155+20^231+13^352+27*)  [8.4](http://gemini.scripps.edu/cgi-bin/SeqCov?/wfs/bfd/3/ian/fly/DrosMelano7227.FASTAC.txt&AAF50543&86+10^162+20^238+13^359+27*) | (AAF50542) CG8532-PB  (Q9VS85) Lqf protein  (AAN12042) CG8532-PC (Fragment)  (AAF50543) CG8532-PA |
| AAF51478 | [7.8](http://molbio.princeton.edu/exchange/Attach/dorsal-x.html#AAF51478) |  |  | [7.8](http://gemini.scripps.edu/cgi-bin/SeqCov?/wfs/bfd/3/ian/fly/DrosMelano7227.FASTAC.txt&AAF51478&7+15^28+28^322+20^465+22^1304+19&28+28*) | (AAF51478) CG2807-PA |
| AAF56042  Q9VCW3 | [2.8](http://molbio.princeton.edu/exchange/Attach/dorsal-x.html#AAF56042) |  |  | [2.8](http://gemini.scripps.edu/cgi-bin/SeqCov?/wfs/bfd/3/ian/fly/DrosMelano7227.FASTAC.txt&AAF56042&911+17^1021+17*)  [2.9](http://gemini.scripps.edu/cgi-bin/SeqCov?/wfs/bfd/3/ian/fly/DrosMelano7227.FASTAC.txt&Q9VCW3&865+17^975+17*) | (AAF56042) CG6958-PA  (Q9VCW3) CG6958 protein |
| AAF56430  Q9VBU7 | [2.8](http://molbio.princeton.edu/exchange/Attach/dorsal-x.html#AAF56430) |  |  | [2.8](http://gemini.scripps.edu/cgi-bin/SeqCov?/wfs/bfd/3/ian/fly/DrosMelano7227.FASTAC.txt&AAF56430&119+15^152+19^1401+18^1697+23*)  [2.8](http://gemini.scripps.edu/cgi-bin/SeqCov?/wfs/bfd/3/ian/fly/DrosMelano7227.FASTAC.txt&Q9VBU7&83+15^116+19^1365+18^1661+23*) | (AAF56430) CG11856-PA  (Q9VBU7) CG11856 protein |
| AAF57941  EF1B_DROME | [14.2](http://molbio.princeton.edu/exchange/Attach/dorsal-x.html#AAF57941) |  |  | [14.2](http://gemini.scripps.edu/cgi-bin/SeqCov?/wfs/bfd/3/ian/fly/DrosMelano7227.FASTAC.txt&AAF57941&53+19^243+18*)  [16.7](http://gemini.scripps.edu/cgi-bin/SeqCov?/wfs/bfd/3/ian/fly/DrosMelano7227.FASTAC.txt&EF1B_DROME&13+19^203+18*) | (AAF57941) CG6341-PA  (O96827) Probable elongation factor 1-beta (EF-1-beta) |
| AAM68880 | [11.9](http://molbio.princeton.edu/exchange/Attach/dorsal-x.html#AAM68880) |  |  | [11.9](http://gemini.scripps.edu/cgi-bin/SeqCov?/wfs/bfd/3/ian/fly/DrosMelano7227.FASTAC.txt&AAM68880&126+16^315+26*) | (AAM68880) CG8722-PA (CG8722-PB) |
| AAN11893  Q9VTD6  Q8IQF2  AAS65039 | [7.9](http://molbio.princeton.edu/exchange/Attach/dorsal-x.html#AAN11893) |  |  | [7.9](http://gemini.scripps.edu/cgi-bin/SeqCov?/wfs/bfd/3/ian/fly/DrosMelano7227.FASTAC.txt&AAN11893&137+20^325+18*)  [7.6](http://gemini.scripps.edu/cgi-bin/SeqCov?/wfs/bfd/3/ian/fly/DrosMelano7227.FASTAC.txt&Q9VTD6&157+20^345+18*)  [8.4](http://gemini.scripps.edu/cgi-bin/SeqCov?/wfs/bfd/3/ian/fly/DrosMelano7227.FASTAC.txt&Q8IQF2&110+20^298+18*)  [7.8](http://gemini.scripps.edu/cgi-bin/SeqCov?/wfs/bfd/3/ian/fly/DrosMelano7227.FASTAC.txt&AAS65039&145+20^333+18*) | (AAN11893) CG6327-PC  (Q9VTD6) CG6327 protein  (Q8IQF2) CG6327-PC  (AAS65039) CG6327-PD |
| AAS64718 | [12.3](http://molbio.princeton.edu/exchange/Attach/dorsal-x.html#AAS64718) |  |  | [12.3](http://gemini.scripps.edu/cgi-bin/SeqCov?/wfs/bfd/3/ian/fly/DrosMelano7227.FASTAC.txt&AAS64718&182+23^258+27^360+16^518+20&182+23*) | (AAS64718) CG10637-PC |
| AAS64882 | [13.2](http://molbio.princeton.edu/exchange/Attach/dorsal-x.html#AAS64882) |  |  | [13.2](http://gemini.scripps.edu/cgi-bin/SeqCov?/wfs/bfd/3/ian/fly/DrosMelano7227.FASTAC.txt&AAS64882&124+33*) | (AAS64882) CG12128-PB |
| AAS64955 | [7.6](http://molbio.princeton.edu/exchange/Attach/dorsal-x.html#AAS64955) |  |  | [7.6](http://gemini.scripps.edu/cgi-bin/SeqCov?/wfs/bfd/3/ian/fly/DrosMelano7227.FASTAC.txt&AAS64955&260+13^305+13^429+29^800+18*) | (AAS64955) CG11505-PC |
| ATC1_DROME | [3.7](http://molbio.princeton.edu/exchange/Attach/dorsal-x.html#ATC1_DROME) |  |  | [3.7](http://gemini.scripps.edu/cgi-bin/SeqCov?/wfs/bfd/3/ian/fly/DrosMelano7227.FASTAC.txt&ATC1_DROME&437+15^729+23*) | (P22700) Calcium-transporting ATPase sarcoplasmic/endoplasmic reticulum type (EC 3.6.3.8) (Calcium pump) |
| CACT_DROME | [22.2](http://molbio.princeton.edu/exchange/Attach/dorsal-x.html#CACT_DROME) |  |  | [22.2](http://gemini.scripps.edu/cgi-bin/SeqCov?/wfs/bfd/3/ian/fly/DrosMelano7227.FASTAC.txt&CACT_DROME&64+36^168+18^365+15^398+42*) | (Q03017) Developmental protein cactus |
| EF1G_DROME | [9.5](http://molbio.princeton.edu/exchange/Attach/dorsal-x.html#EF1G_DROME) |  |  | [9.5](http://gemini.scripps.edu/cgi-bin/SeqCov?/wfs/bfd/3/ian/fly/DrosMelano7227.FASTAC.txt&EF1G_DROME&18+13^55+28*) | (Q9NJH0) Elongation factor 1-gamma (EF-1-gamma) (eEF-1B gamma) |
| HTS_DROME | [3.2](http://molbio.princeton.edu/exchange/Attach/dorsal-x.html#HTS_DROME) |  |  | [3.2](http://gemini.scripps.edu/cgi-bin/SeqCov?/wfs/bfd/3/ian/fly/DrosMelano7227.FASTAC.txt&HTS_DROME&129+18^174+19*) | (Q02645) Hu-li tai shao protein (Adducin-like protein) |
| HYD_DROME | [4.0](http://molbio.princeton.edu/exchange/Attach/dorsal-x.html#HYD_DROME) |  |  | [4.0](http://gemini.scripps.edu/cgi-bin/SeqCov?/wfs/bfd/3/ian/fly/DrosMelano7227.FASTAC.txt&HYD_DROME&179+15^695+27^758+20^925+13^1831+15^2131+14^2230+12*) | (P51592) Ubiquitin--protein ligase hyd (EC 6.3.2.-) (Hyperplastic discs protein) |
| IMA_DROME | [6.7](http://molbio.princeton.edu/exchange/Attach/dorsal-x.html#IMA_DROME) |  |  | [6.7](http://gemini.scripps.edu/cgi-bin/SeqCov?/wfs/bfd/3/ian/fly/DrosMelano7227.FASTAC.txt&IMA_DROME&294+35&305+24&305+24*) | (P52295) Importin alpha subunit (Karyopherin alpha subunit) (Pendulin) |
| IMB_DROME | [12.8](http://molbio.princeton.edu/exchange/Attach/dorsal-x.html#IMB_DROME) |  |  | [12.8](http://gemini.scripps.edu/cgi-bin/SeqCov?/wfs/bfd/3/ian/fly/DrosMelano7227.FASTAC.txt&IMB_DROME&45+16^154+15^198+15^666+20^810+47*) | (O18388) Importin beta subunit (Karyopherin beta-4 subunit) (Protein ketel) |
| NADE_DROME | [10.5](http://molbio.princeton.edu/exchange/Attach/dorsal-x.html#NADE_DROME) |  |  | [10.5](http://gemini.scripps.edu/cgi-bin/SeqCov?/wfs/bfd/3/ian/fly/DrosMelano7227.FASTAC.txt&NADE_DROME&5+21^167+16^258+22^347+24*) | (Q9VYA0) Putative glutamine-dependent NAD(+) synthetase (EC 6.3.5.1) (NAD(+) synthase [glutamine-hydrolyzing]) |
| PORI_DROME | [22.7](http://molbio.princeton.edu/exchange/Attach/dorsal-x.html#PORI_DROME) |  |  | [22.7](http://gemini.scripps.edu/cgi-bin/SeqCov?/wfs/bfd/3/ian/fly/DrosMelano7227.FASTAC.txt&PORI_DROME&74+22^134+42&74+22*) | (Q94920) Voltage-dependent anion-selective channel (Porin protein) |
| Q8IPU3  Q9VPB3 | [9.5](http://molbio.princeton.edu/exchange/Attach/dorsal-x.html#Q8IPU3) |  |  | [9.5](http://gemini.scripps.edu/cgi-bin/SeqCov?/wfs/bfd/3/ian/fly/DrosMelano7227.FASTAC.txt&Q8IPU3&8+16^94+17*)  [10.8](http://gemini.scripps.edu/cgi-bin/SeqCov?/wfs/bfd/3/ian/fly/DrosMelano7227.FASTAC.txt&Q9VPB3&8+16^51+17*) | (Q8IPU3) CG4365-PC  (Q9VPB3) CG4365 protein (LD26447p) |
| Q8IR99  Q9VZ69 | [7.7](http://molbio.princeton.edu/exchange/Attach/dorsal-x.html#Q8IR99) |  |  | [7.7](http://gemini.scripps.edu/cgi-bin/SeqCov?/wfs/bfd/3/ian/fly/DrosMelano7227.FASTAC.txt&Q8IR99&89+14^149+14^198+16*)  [7.8](http://gemini.scripps.edu/cgi-bin/SeqCov?/wfs/bfd/3/ian/fly/DrosMelano7227.FASTAC.txt&Q9VZ69&82+14^142+14^191+16*) | (Q8IR99) CG1691-PD  (Q9VZ69) CG1691 protein (IGF-II mRNA-binding protein) (SD07045p) |
| Q8MRM4  Q9VPU2 | [12.3](http://molbio.princeton.edu/exchange/Attach/dorsal-x.html#Q8MRM4) |  |  | [12.3](http://gemini.scripps.edu/cgi-bin/SeqCov?/wfs/bfd/3/ian/fly/DrosMelano7227.FASTAC.txt&Q8MRM4&196+21^306+34*)  [10.3](http://gemini.scripps.edu/cgi-bin/SeqCov?/wfs/bfd/3/ian/fly/DrosMelano7227.FASTAC.txt&Q9VPU2&196+21^306+34*) | (Q8MRM4) GH16240p (CG3365-PC)  (Q9VPU2) Drongo protein |
| Q8T4C4 | [5.3](http://molbio.princeton.edu/exchange/Attach/dorsal-x.html#Q8T4C4) |  |  | [5.3](http://gemini.scripps.edu/cgi-bin/SeqCov?/wfs/bfd/3/ian/fly/DrosMelano7227.FASTAC.txt&Q8T4C4&355+14^477+19*) | (Q8T4C4) AT04467p (CG5389-PA) |
| Q95TP2 | [6.0](http://molbio.princeton.edu/exchange/Attach/dorsal-x.html#Q95TP2) |  |  | [6.0](http://gemini.scripps.edu/cgi-bin/SeqCov?/wfs/bfd/3/ian/fly/DrosMelano7227.FASTAC.txt&Q95TP2&160+34^216+23*) | (Q95TP2) LD33980p (CG10637-PB) |
| Q9I7T7  Q9VZS2 | [11.7](http://molbio.princeton.edu/exchange/Attach/dorsal-x.html#Q9I7T7) |  |  | [11.7](http://gemini.scripps.edu/cgi-bin/SeqCov?/wfs/bfd/3/ian/fly/DrosMelano7227.FASTAC.txt&Q9I7T7&270+13^315+13^439+29^810+18^1131+27^1202+25^1364+19^1448+35&1202+25^1461+22*)  [11.8](http://gemini.scripps.edu/cgi-bin/SeqCov?/wfs/bfd/3/ian/fly/DrosMelano7227.FASTAC.txt&Q9VZS2&260+13^305+13^429+29^800+18^1121+27^1192+25^1354+19^1438+35&1192+25^1451+22*) | (Q9I7T7) CG11505 protein  (Q9VZS2) CG11505 protein |
| Q9V346  Q9V347 | [1.4](http://molbio.princeton.edu/exchange/Attach/dorsal-x.html#Q9V346) |  |  | [1.4](http://gemini.scripps.edu/cgi-bin/SeqCov?/wfs/bfd/3/ian/fly/DrosMelano7227.FASTAC.txt&Q9V346&1230+15^2307+17*)  [1.3](http://gemini.scripps.edu/cgi-bin/SeqCov?/wfs/bfd/3/ian/fly/DrosMelano7227.FASTAC.txt&Q9V347&1389+15^2466+17*) | (Q9V346) CG11198 protein (GH12002p)  (Q9V347) CG11198 protein |
| Q9V369 | [10.1](http://molbio.princeton.edu/exchange/Attach/dorsal-x.html#Q9V369) |  |  | [10.1](http://gemini.scripps.edu/cgi-bin/SeqCov?/wfs/bfd/3/ian/fly/DrosMelano7227.FASTAC.txt&Q9V369&149+23^179+18^499+16&159+13*) | (Q9V369) CG2158 protein (LD27030p) |
| Q9V455 | [19.8](http://molbio.princeton.edu/exchange/Attach/dorsal-x.html#Q9V455) |  |  | [19.8](http://gemini.scripps.edu/cgi-bin/SeqCov?/wfs/bfd/3/ian/fly/DrosMelano7227.FASTAC.txt&Q9V455&47+22^99+21^302+33^382+15^460+11&306+29*) | (Q9V455) Karyopherin-ALPHA3 protein (LD13917p) |
| Q9V5E9 | [10.1](http://molbio.princeton.edu/exchange/Attach/dorsal-x.html#Q9V5E9) |  |  | [10.1](http://gemini.scripps.edu/cgi-bin/SeqCov?/wfs/bfd/3/ian/fly/DrosMelano7227.FASTAC.txt&Q9V5E9&108+16^359+33*) | (Q9V5E9) CG12128 protein |
| Q9V5R0 | [8.3](http://molbio.princeton.edu/exchange/Attach/dorsal-x.html#Q9V5R0) |  |  | [8.3](http://gemini.scripps.edu/cgi-bin/SeqCov?/wfs/bfd/3/ian/fly/DrosMelano7227.FASTAC.txt&Q9V5R0&309+13^329+25*) | (Q9V5R0) CG6751 protein |
| Q9V725 | [19.0](http://molbio.princeton.edu/exchange/Attach/dorsal-x.html#Q9V725) |  |  | [19.0](http://gemini.scripps.edu/cgi-bin/SeqCov?/wfs/bfd/3/ian/fly/DrosMelano7227.FASTAC.txt&Q9V725&30+24^118+28^228+10^496+45&30+24^130+16*) | (Q9V725) CG10109 protein (RE53130p) (LOBE) |
| Q9V8D2 | [5.4](http://molbio.princeton.edu/exchange/Attach/dorsal-x.html#Q9V8D2) |  |  | [5.4](http://gemini.scripps.edu/cgi-bin/SeqCov?/wfs/bfd/3/ian/fly/DrosMelano7227.FASTAC.txt&Q9V8D2&184+22^285+14*) | (Q9V8D2) CG5733 protein |
| Q9VCY1 | [8.1](http://molbio.princeton.edu/exchange/Attach/dorsal-x.html#Q9VCY1) |  |  | [8.1](http://gemini.scripps.edu/cgi-bin/SeqCov?/wfs/bfd/3/ian/fly/DrosMelano7227.FASTAC.txt&Q9VCY1&93+14^196+15*) | (Q9VCY1) CG6937 protein |
| Q9VD07 | [4.0](http://molbio.princeton.edu/exchange/Attach/dorsal-x.html#Q9VD07) |  |  | [4.0](http://gemini.scripps.edu/cgi-bin/SeqCov?/wfs/bfd/3/ian/fly/DrosMelano7227.FASTAC.txt&Q9VD07&49+22^78+21^248+17^1086+22*) | (Q9VD07) CG12499 protein |
| Q9VEJ0 | [41.9](http://molbio.princeton.edu/exchange/Attach/dorsal-x.html#Q9VEJ0) |  |  | [41.9](http://gemini.scripps.edu/cgi-bin/SeqCov?/wfs/bfd/3/ian/fly/DrosMelano7227.FASTAC.txt&Q9VEJ0&51+13^76+20^98+18^144+13^195+34&53+11^98+17^144+12&54+10^145+11&145+11&145+11*) | (Q9VEJ0) CG5826 protein (Thioredoxin peroxidase 3) (SD08737p) |
| Q9VEP9 | [4.2](http://molbio.princeton.edu/exchange/Attach/dorsal-x.html#Q9VEP9) |  |  | [4.2](http://gemini.scripps.edu/cgi-bin/SeqCov?/wfs/bfd/3/ian/fly/DrosMelano7227.FASTAC.txt&Q9VEP9&458+18^482+15*) | (Q9VEP9) CG16941 protein (GH03554P) |
| Q9VJ30 | [9.6](http://molbio.princeton.edu/exchange/Attach/dorsal-x.html#Q9VJ30) |  |  | [9.6](http://gemini.scripps.edu/cgi-bin/SeqCov?/wfs/bfd/3/ian/fly/DrosMelano7227.FASTAC.txt&Q9VJ30&182+23^258+27^360+16^518+20^698+34^754+23&182+23*) | (Q9VJ30) CG10637 protein |
| Q9VK45 | [17.7](http://molbio.princeton.edu/exchange/Attach/dorsal-x.html#Q9VK45) |  |  | [17.7](http://gemini.scripps.edu/cgi-bin/SeqCov?/wfs/bfd/3/ian/fly/DrosMelano7227.FASTAC.txt&Q9VK45&25+10^77+12^100+16^128+10^224+18^252+13^289+20^459+16^486+20^534+64^635+14^708+11^814+16^1287+19^1375+15^1450+16^1479+19^1536+17^1850+17^1940+18^1998+11^2205+18^2307+12^2320+10^2439+25&540+26&540+26*) | (Q9VK45) CG5092 protein |
| Q9VKQ3 | [7.9](http://molbio.princeton.edu/exchange/Attach/dorsal-x.html#Q9VKQ3) |  |  | [7.9](http://gemini.scripps.edu/cgi-bin/SeqCov?/wfs/bfd/3/ian/fly/DrosMelano7227.FASTAC.txt&Q9VKQ3&51+18^220+15*) | (Q9VKQ3) CG6724 protein (GM29372p) |
| Q9VKW4 | [7.8](http://molbio.princeton.edu/exchange/Attach/dorsal-x.html#Q9VKW4) |  |  | [7.8](http://gemini.scripps.edu/cgi-bin/SeqCov?/wfs/bfd/3/ian/fly/DrosMelano7227.FASTAC.txt&Q9VKW4&76+23^384+13^457+28^1029+18*) | (Q9VKW4) CG5300 protein (LD03769p) |
| Q9VPR5 | [6.6](http://molbio.princeton.edu/exchange/Attach/dorsal-x.html#Q9VPR5) |  |  | [6.6](http://gemini.scripps.edu/cgi-bin/SeqCov?/wfs/bfd/3/ian/fly/DrosMelano7227.FASTAC.txt&Q9VPR5&37+28^331+20^474+22^1313+19&37+28*) | (Q9VPR5) CG2807 protein |
| Q9VPU0 | [9.0](http://molbio.princeton.edu/exchange/Attach/dorsal-x.html#Q9VPU0) |  |  | [9.0](http://gemini.scripps.edu/cgi-bin/SeqCov?/wfs/bfd/3/ian/fly/DrosMelano7227.FASTAC.txt&Q9VPU0&46+19^105+22*) | (Q9VPU0) CG3862 protein |
| Q9VPU3 | [11.8](http://molbio.princeton.edu/exchange/Attach/dorsal-x.html#Q9VPU3) |  |  | [11.8](http://gemini.scripps.edu/cgi-bin/SeqCov?/wfs/bfd/3/ian/fly/DrosMelano7227.FASTAC.txt&Q9VPU3&68+16^266+21^376+34*) | (Q9VPU3) Drongo protein |
| Q9VT60 | [8.8](http://molbio.princeton.edu/exchange/Attach/dorsal-x.html#Q9VT60) |  |  | [8.8](http://gemini.scripps.edu/cgi-bin/SeqCov?/wfs/bfd/3/ian/fly/DrosMelano7227.FASTAC.txt&Q9VT60&142+20^200+14^321+18^756+22&756+22*) | (Q9VT60) CG6718 protein |
| Q9VXE6 | [7.4](http://molbio.princeton.edu/exchange/Attach/dorsal-x.html#Q9VXE6) |  |  | [7.4](http://gemini.scripps.edu/cgi-bin/SeqCov?/wfs/bfd/3/ian/fly/DrosMelano7227.FASTAC.txt&Q9VXE6&521+23^953+30^1180+25^1316+31^1507+32*) | (Q9VXE6) CG4453 protein |
| Q9VYW3 | [28.6](http://molbio.princeton.edu/exchange/Attach/dorsal-x.html#Q9VYW3) |  |  | [28.6](http://gemini.scripps.edu/cgi-bin/SeqCov?/wfs/bfd/3/ian/fly/DrosMelano7227.FASTAC.txt&Q9VYW3&29+18^79+14*) | (Q9VYW3) CG15220 protein (RH55360p) |
| Q9W2F2 | [2.3](http://molbio.princeton.edu/exchange/Attach/dorsal-x.html#Q9W2F2) |  |  | [2.3](http://gemini.scripps.edu/cgi-bin/SeqCov?/wfs/bfd/3/ian/fly/DrosMelano7227.FASTAC.txt&Q9W2F2&1150+17^1526+19*) | (Q9W2F2) CG10080 protein |
| Q9W2T1 | [2.0](http://molbio.princeton.edu/exchange/Attach/dorsal-x.html#Q9W2T1) |  |  | [2.0](http://gemini.scripps.edu/cgi-bin/SeqCov?/wfs/bfd/3/ian/fly/DrosMelano7227.FASTAC.txt&Q9W2T1&1126+25^1518+17*) | (Q9W2T1) CG9817 protein |
| Q9W2Y5 | [2.0](http://molbio.princeton.edu/exchange/Attach/dorsal-x.html#Q9W2Y5) |  |  | [2.0](http://gemini.scripps.edu/cgi-bin/SeqCov?/wfs/bfd/3/ian/fly/DrosMelano7227.FASTAC.txt&Q9W2Y5&1590+17^1642+20*) | (Q9W2Y5) CG32685 protein |
| Q9W328 | [23.0](http://molbio.princeton.edu/exchange/Attach/dorsal-x.html#Q9W328) |  |  | [23.0](http://gemini.scripps.edu/cgi-bin/SeqCov?/wfs/bfd/3/ian/fly/DrosMelano7227.FASTAC.txt&Q9W328&34+12^160+19^228+15^270+12^300+14&162+17*) | (Q9W328) CG3004 protein (LD23129p) |
| Q9W3M3 | [10.7](http://molbio.princeton.edu/exchange/Attach/dorsal-x.html#Q9W3M3) |  |  | [10.7](http://gemini.scripps.edu/cgi-bin/SeqCov?/wfs/bfd/3/ian/fly/DrosMelano7227.FASTAC.txt&Q9W3M3&57+13^78+24^336+44^763+34^983+10^1357+15^1413+17&78+24*) | (Q9W3M3) CG1531 protein |
| Q9W437 | [18.8](http://molbio.princeton.edu/exchange/Attach/dorsal-x.html#Q9W437) |  |  | [18.8](http://gemini.scripps.edu/cgi-bin/SeqCov?/wfs/bfd/3/ian/fly/DrosMelano7227.FASTAC.txt&Q9W437&9+30^257+15^387+31^694+13^794+17^821+19^900+28^1293+45^1388+36^1460+14^1524+37^1601+21&794+17^900+28*) | (Q9W437) CG4320 protein |
| Q9XYZ5 | [10.3](http://molbio.princeton.edu/exchange/Attach/dorsal-x.html#Q9XYZ5) |  |  | [10.3](http://gemini.scripps.edu/cgi-bin/SeqCov?/wfs/bfd/3/ian/fly/DrosMelano7227.FASTAC.txt&Q9XYZ5&532+19^696+13^723+16^822+24^957+24^1083+21*) | (Q9XYZ5) DDB1 protein |
| YU20_DROME | [4.2](http://molbio.princeton.edu/exchange/Attach/dorsal-x.html#YU20_DROME) |  |  | [4.2](http://gemini.scripps.edu/cgi-bin/SeqCov?/wfs/bfd/3/ian/fly/DrosMelano7227.FASTAC.txt&YU20_DROME&616+13^741+19*) | (Q9VIF0) UPF0120 protein CG9246 |
| **57** | **X** |  |  |  |  |

Criteria used to score proteins:

| | true | Use criteria | | --- | --- | | 2.0 | Minimum +1 XCorr | | 3.0 | Minimum +2 XCorr | | 3.5 | Minimum +3 XCorr | | 0.1 | Minimum DeltCN | | 1 | Minimum charge state | | 3 | Maximum charge state | | 0.0 | Minimum ion proportion | | 1000 | Maximum Sp rank | | -1.0 | Minimum Sp score | | Include | Modified peptide inclusion | | Any | Tryptic status requirement | | true | Multiple, ambiguous IDs allowed | | Ignore | Peptide validation handling | | XCorr | Purge duplicate peptides by protein | | false | Include only loci with unique peptide | | false | Remove subset proteins | | Ignore | Locus validation handling | | 0 | Minimum modified peptides per locus | | 10 | Minimum redundancy for low coverage loci | | 2 | Minimum peptides per locus | |
| --- | --- | --- | --- | --- | --- | --- | --- | --- | --- | --- | --- | --- | --- | --- | --- | --- | --- | --- | --- | --- | --- | --- | --- | --- | --- | --- | --- | --- | --- | --- | --- | --- | --- | --- | --- | --- | --- | --- | --- | --- | --- | --- |

| ***Redundant Count*** | ***Nonredundant Count*** | ***Percent*** | ***dorsal x*** | ***orb x*** | ***rin x*** |
| --- | --- | --- | --- | --- | --- |
| [63](http://molbio.princeton.edu/exchange/Attach/read.asp?obj=00000000306A64AFE42DD111817700A0244886C30700415DA34905A2D011814A00A0244886C3000000074F8900005642616551FFB140B0086023731A558000000259E4960000&att=ATT-0-CEFB6D512F8372468FED0004925D5E91-Contrast.htm#7) | [55](http://molbio.princeton.edu/exchange/Attach/read.asp?obj=00000000306A64AFE42DD111817700A0244886C30700415DA34905A2D011814A00A0244886C3000000074F8900005642616551FFB140B0086023731A558000000259E4960000&att=ATT-0-CEFB6D512F8372468FED0004925D5E91-Contrast.htm#7) | 17.5% | X | X | X |
| [28](http://molbio.princeton.edu/exchange/Attach/read.asp?obj=00000000306A64AFE42DD111817700A0244886C30700415DA34905A2D011814A00A0244886C3000000074F8900005642616551FFB140B0086023731A558000000259E4960000&att=ATT-0-CEFB6D512F8372468FED0004925D5E91-Contrast.htm#6) | [23](http://molbio.princeton.edu/exchange/Attach/read.asp?obj=00000000306A64AFE42DD111817700A0244886C30700415DA34905A2D011814A00A0244886C3000000074F8900005642616551FFB140B0086023731A558000000259E4960000&att=ATT-0-CEFB6D512F8372468FED0004925D5E91-Contrast.htm#6) | 7.3% |  | X | X |
| [5](http://molbio.princeton.edu/exchange/Attach/read.asp?obj=00000000306A64AFE42DD111817700A0244886C30700415DA34905A2D011814A00A0244886C3000000074F8900005642616551FFB140B0086023731A558000000259E4960000&att=ATT-0-CEFB6D512F8372468FED0004925D5E91-Contrast.htm#5) | [4](http://molbio.princeton.edu/exchange/Attach/read.asp?obj=00000000306A64AFE42DD111817700A0244886C30700415DA34905A2D011814A00A0244886C3000000074F8900005642616551FFB140B0086023731A558000000259E4960000&att=ATT-0-CEFB6D512F8372468FED0004925D5E91-Contrast.htm#5) | 1.3% | X |  | X |
| [24](http://molbio.princeton.edu/exchange/Attach/read.asp?obj=00000000306A64AFE42DD111817700A0244886C30700415DA34905A2D011814A00A0244886C3000000074F8900005642616551FFB140B0086023731A558000000259E4960000&att=ATT-0-CEFB6D512F8372468FED0004925D5E91-Contrast.htm#3) | [21](http://molbio.princeton.edu/exchange/Attach/read.asp?obj=00000000306A64AFE42DD111817700A0244886C30700415DA34905A2D011814A00A0244886C3000000074F8900005642616551FFB140B0086023731A558000000259E4960000&att=ATT-0-CEFB6D512F8372468FED0004925D5E91-Contrast.htm#3) | 6.7% | X | X |  |
| [33](http://molbio.princeton.edu/exchange/Attach/read.asp?obj=00000000306A64AFE42DD111817700A0244886C30700415DA34905A2D011814A00A0244886C3000000074F8900005642616551FFB140B0086023731A558000000259E4960000&att=ATT-0-CEFB6D512F8372468FED0004925D5E91-Contrast.htm#4) | [22](http://molbio.princeton.edu/exchange/Attach/read.asp?obj=00000000306A64AFE42DD111817700A0244886C30700415DA34905A2D011814A00A0244886C3000000074F8900005642616551FFB140B0086023731A558000000259E4960000&att=ATT-0-CEFB6D512F8372468FED0004925D5E91-Contrast.htm#4) | 7.0% |  |  | X |
| [176](http://molbio.princeton.edu/exchange/Attach/read.asp?obj=00000000306A64AFE42DD111817700A0244886C30700415DA34905A2D011814A00A0244886C3000000074F8900005642616551FFB140B0086023731A558000000259E4960000&att=ATT-0-CEFB6D512F8372468FED0004925D5E91-Contrast.htm#2) | [132](http://molbio.princeton.edu/exchange/Attach/read.asp?obj=00000000306A64AFE42DD111817700A0244886C30700415DA34905A2D011814A00A0244886C3000000074F8900005642616551FFB140B0086023731A558000000259E4960000&att=ATT-0-CEFB6D512F8372468FED0004925D5E91-Contrast.htm#2) | 42.0% |  | X |  |
| [73](http://molbio.princeton.edu/exchange/Attach/read.asp?obj=00000000306A64AFE42DD111817700A0244886C30700415DA34905A2D011814A00A0244886C3000000074F8900005642616551FFB140B0086023731A558000000259E4960000&att=ATT-0-CEFB6D512F8372468FED0004925D5E91-Contrast.htm#1) | [57](http://molbio.princeton.edu/exchange/Attach/read.asp?obj=00000000306A64AFE42DD111817700A0244886C30700415DA34905A2D011814A00A0244886C3000000074F8900005642616551FFB140B0086023731A558000000259E4960000&att=ATT-0-CEFB6D512F8372468FED0004925D5E91-Contrast.htm#1) | 18.2% | X |  |  |
| 402 | 314 |  | 137 | 231 | 104 |

***Percentage of the row sample proteins which are found in the column sample.***

| ***Sample*** | ***Proteins*** | ***dorsal-x*** | ***orb-x*** | ***rin-x*** |
| --- | --- | --- | --- | --- |
| dorsal-x | 137 | 100.0% | 55.5% | 43.1% |
| orb-x | 231 | 32.9% | 100.0% | 33.8% |
| rin-x | 104 | 56.7% | 75.0% | 100.0% |
