# Supplementary material for: Rasputin Functions as a Positive Regulator of Orb in Drosophila Oogenesis
Source: PLoS One. 2013 Sep 12;8(9):e72864. doi: 10.1371/journal.pone.0072864 (PMC3771913; doi:10.1371/journal.pone.0072864)
Supplement: Table S3 — Potentially relevant proteins present in Orb, Rin and also Dorsal Immunoprecipitates. In addition to ribosomal proteins, a number of these proteins have been found associated with Rin homologs in other species or have been implicated in translation regulation. (DOC) [file pone.0072864.s008.doc]

**Table S3. Potentially relevant proteins present in Orb, Rin and also Dorsal Immunoprecipitates.** I

| CG18811 | Caprin |
| --- | --- |
| CG17489 | 60S ribosomal protein L15 |
| CG4869 | B-tubulin |
| CG2168 | ribosomal protein S3A |
| CG7808 | ribosomal protein S8 |
| CG4869 | B-tubulin 97E |
| CG8415 | ribosomal protein S23 |
| CG9227 | B-tubulin 56D |
| CG5519 | Ubiquitin ligase Prp19 |
| CG2331 | TERA94 cytoskelton |
| CG7014 | ribosomal protein S5b |
| CG10652 | ribosomal protein L30 |
| CG14648 | Lost AP axis |
| CG8615 | ribosomal protein L18 |
| CG3195 | ribosomal protein L12 |
| CG9888 | Fibrillarin mRNA binding |
| CG5502 | ribosomal protein S23 |
| CG6141 | ribosomal protein L9 |
| CG5920 | ribosomal protein S5 |
| CG10686 | trailer hitch oocyte DV axis |
| CG11181 | cup translational regulator |
| CG17489 | ribosomal protein L15 |
